# Supplementary material for: How can we engineer electronic transitions through twisting and stacking in TMDC bilayers and heterostructures? a first-principles approach
Source: Nanoscale Adv. 2025 Feb 13;7(7):2047–56. doi: 10.1039/d5na00112a (PMC11833679; doi:10.1039/d5na00112a)
Supplement: NA-007-D5NA00112A-s001 [file NA-007-D5NA00112A-s001.pdf]

Supplementary Information (SI) for Nanoscale Advances.
This journal is © The Royal Society of Chemistry 2025

Supporting Information

How Can We Engineer Electronic Transitions Through Twisting and Stacking in TMDC Bilayers and Heterostructures? A First-Principles Approach

Yu-Hsiu Lin,[1] William P. Comaskey,[2,3] and Jose L. Mendoza-Cortes[4,1,*]

[1] *Department of Chemical Engineering & Materials Science, Michigan State University, East Lansing, Michigan 48824, United States.*
[2] *Department of Health Physics, University of Nevada, Las Vegas, Las Vegas, NV 89154, USA*
[3] *National High Magnetic Field Laboratory, Tallahassee, FL 32310, USA*
[4] *Department of Physics and Astronomy, Michigan State University, East Lansing, Michigan 48824, United States.*

(Dated: February 1, 2025)

* jmendoza@msu.edu

CONTENTS

List of Figures 5

I. Optimized Geometries of relevant bilayers 16
1. 1T-MoS_2/1T-MoS_2-0 16
2. 1T-MoS_2/1T-MoS_2-27.8 17
3. 1T-MoS_2/1T-MoS_2-38.2 18
4. 1T-MoS_2/1T-MoS_2-60 19
5. 1T-MoS_2/1T-MoS_2-92.2 20
6. 1T-MoS_2/1T-MoS_2-98.2 21
7. 1T-MoS_2/1T-MoS_2-120 22
8. 1H-MoS_2/1H-MoS_2-0 23
9. 1H-MoS_2/1H-MoS_2-27.8 24
10. 1H-MoS_2/1H-MoS_2-38.2 25
11. 1H-MoS_2/1H-MoS_2-60 26
12. 1H-MoS_2/1H-MoS_2-98.2 27
13. 1H-MoS_2/1H-MoS_2-120 28
14. $MoSe_2$/$MoSe_2$-0 29
15. $MoSe_2$/$MoSe_2$-27.8 30
16. $MoSe_2$/$MoSe_2$-32.2 31
17. $MoSe_2$/$MoSe_2$-60 32
18. $MoSe_2$/$MoSe_2$-92.2 33
19. $MoSe_2$/$MoSe_2$-120 34
20. $MoTe_2$/$MoTe_2$-0 35
21. $MoTe_2$/$MoTe_2$-17.9 36
22. $MoTe_2$/$MoTe_2$-27.8 37
23. $MoTe_2$/$MoTe_2$-32.2 38
24. $MoTe_2$/$MoTe_2$-60 39
25. $MoTe_2$/$MoTe_2$-87.8 40
26. $MoTe_2$/$MoTe_2$-92.2 41
27. $MoTe_2$/$MoTe_2$-120 42
28. WS_2/WS_2-0 43
29. WS_2/WS_2-17.9 44
30. WS_2/WS_2-27.8 45
31. WS_2/WS_2-32.2 46
32. WS_2/WS_2-42.1 47
33. WS_2/WS_2-60 48
34. WS_2/WS_2-77.9 49
35. WS_2/WS_2-87.8 50
36. WS_2/WS_2-102.1 51
37. WS_2/WS_2-120 52
38. WSe_2/WSe_2-0 53
39. WSe_2/WSe_2-17.9 54
40. WSe_2/WSe_2-27.8 55
41. WSe_2/WSe_2-32.2 56
42. WSe_2/WSe_2-42.1 57
43. WSe_2/WSe_2-60 58
44. WSe_2/WSe_2-77.9 59
45. WSe_2/WSe_2-87.8 60
46. WSe_2/WSe_2-102.1 61
47. WSe_2/WSe_2-120 62
48. WTe_2/WTe_2-0 63
49. WTe_2/WTe_2-17.9 64
50. WTe_2/WTe_2-27.8 65
51. WTe_2/WTe_2-32.2 66
52. WTe_2/WTe_2-42.1 67
53. WTe_2/WTe_2-60 68

54. WTe_2/WTe_2-77.9 69
55. WTe_2/WTe_2-87.8 70
56. WTe_2/WTe_2-92.2 71
57. WTe_2/WTe_2-102.1 72
58. WTe_2/WTe_2-120 73
59. 1T-$MoS_2/MoSe_2$-0 74
60. 1T-$MoS_2/MoSe_2$-60 75
61. 1H-$MoS_2/MoSe_2$-0 76
62. 1H-$MoS_2/MoSe_2$-60 77
63. 1T-$MoS_2/MoTe_2$-0 78
64. 1T-$MoS_2/MoTe_2$-60 79
65. 1H-$MoS_2/MoTe_2$-0 80
66. 1H-$MoS_2/MoTe_2$-60 81
67. 1T-MoS_2/WS_2-0 82
68. 1T-MoS_2/WS_2-60 83
69. 1H-MoS_2/WS_2-0 84
70. 1H-MoS_2/WS_2-60 85
71. 1T-MoS_2/WSe_2-0 86
72. 1T-MoS_2/WSe_2-60 87
73. 1H-MoS_2/WSe_2-0 88
74. 1H-MoS_2/WSe_2-60 89
75. 1T-MoS_2/WTe_2-0 90
76. 1T-MoS_2/WTe_2-60 91
77. 1H-MoS_2/WTe_2-0 92
78. 1H-MoS_2/WTe_2-60 93
79. $MoSe_2/MoTe_2$-0 94
80. $MoSe_2/MoTe_2$-60 95
81. $MoSe_2/WS_2$-0 96
82. $MoSe_2/WS_2$-60 97
83. $MoSe_2/WSe_2$-0 98
84. $MoSe_2/WSe_2$-60 99
85. $MoSe_2/WTe_2$-0 100
86. $MoSe_2/WTe_2$-60 101
87. $MoTe_2/WS_2$-0 102
88. $MoTe_2/WS_2$-60 103
89. $MoTe_2/WSe_2$-0 104
90. $MoTe_2/WSe_2$-60 105
91. $MoTe_2/WTe_2$-0 106
92. $MoTe_2/WTe_2$-60 108
93. WSe_2/WS_2-0 109
94. WSe_2/WS_2-60 110
95. WTe_2/WS_2-0 111
96. WTe_2/WS_2-60 112
97. WTe_2/WSe_2-0 113
98. WTe_2/WSe_2-60 114
99. 1T-MoS_2/1T-MoS_2-AA 115
100. 1T-MoS_2/1T-MoS_2-AB 116
101. 1H-MoS_2/1H-MoS_2-AA 117
102. 1H-MoS_2/1H-MoS_2-AB 118
103. $MoSe_2/MoSe_2$-AA 119
104. $MoSe_2/MoSe_2$-AB 120
105. $MoTe_2/MoTe_2$-AA 121
106. $MoTe_2/MoTe_2$-AB 122
107. WS_2/WS_2-AA 123
108. WS_2/WS_2-AB 124
109. WSe_2/WSe_2-AA 125
110. WSe_2/WSe_2-AB 126
111. WTe_2/WTe_2-AA 127

112. WTe_2/WTe_2-AB 128
113. 1T-MoS_2 129
114. 1H-MoS_2 130
115. $MoSe_2$ 131
116. $MoTe_2$ 132
117. WS_2 133
118. WSe_2 134
119. WTe_2 135

References 136

LIST OF FIGURES

1 Binding energy are shown for 15 bilayer TMDCs heterostructures with two types of stacking, either 0 or 60 degrees of shifting. 6
2 The E_g of 15 bilayer TMDCs heterostructures compared with two types of stacking, either 0 or 60 degrees of shifting. Hollow signs represent indirect E_g while filled sign stands for direct E_g. Compared to this plot where 1T-MoS_2 was applied, 1H-MoS_2 was applied in Figure 2. 7
3 The E_g are shown of 6 fully optimized TMDCs in forms of monolayer, A-A stacking bilayer, and A-B stacking bilayer. The MoS_2 in this plot belongs to 1T instead of 1H. Hollow signs represent indirect E_g while filled signs stand for direct E_g. 7
4 The overall fluctuation of strain according to the twisted angles of 1H bilayers are shown for the cases of (a) MoS_2, (b) $MoSe_2$, (c) $MoTe_2$, (d) WS_2, (e) WSe_2, and (f) WTe_2. 8
5 The zoom-in fluctuation of strain below 0.001 according to the twisted angles of 1H bilayers in the cases of (a) MoS_2, (b) $MoSe_2$, (c) $MoTe_2$, (d) WS_2, (e) WSe_2, and (f) WTe_2. The low-strain twisted TMDCs bilayers being symmetrized and further computed in this work are dotted in the plots. 9
6 (a) The overall fluctuation of strain according to the twisted angles of bilayers in the cases of 1T-MoS_2. (b) The zoom-in fluctuation of strain below 0.001 according to the twisted angles of bilayers in the cases of 1T-MoS_2. The low-strain twisted TMDCs bilayers being symmetrized and further computed in this work are dotted in the plots. 9
7 The schematic geometries and band structures of bilayer 1H-MoS_2 with the twisted angles of (a) 0° , (b) 27.8° , (c) 38.2° , (d) 60° , and (e) 98.2° . These include the top view and the side view of geometries (bottom left). 10
8 The schematic geometries and band structures of bilayer 1T-MoS_2 with the twisted angles of (a) 0° , (b) 27.8° , (c) 38.2° , (d) 60° , and (e) 92.2° . These include the top view and the side view of geometries (bottom left). 11
9 The schematic geometries and band structures of bilayer $MoSe_2$ with the twisted angles of (a) 0° , (b) 27.8° , (c) 32.2° , and (d) 60° . These include the top view and the side view of geometries (bottom left). 11
10 The schematic geometries and band structures of bilayer $MoTe_2$ with the twisted angles of (a) 0° , (b) 17.9° , (c) 27.8° , (d) 32.2° , and (e) 60° . These include the top view and the side view of geometries (bottom left). 12
11 The schematic geometries and band structures of bilayer WS_2 with the twisted angles of (a) 0° , (b) 17.9° , (c) 27.8° , (d) 32.2° , (e) 42.1° , and (f) 60° . These include the top view and the side view of geometries (bottom left). 13
12 The schematic geometries and band structures of bilayer WTe_2 with the twisted angles of (a) 0° , (b) 17.9° , (c) 27.8° , (d) 32.2° , (e) 42.1° , and (f) 60° . These include the top view and the side view of geometries (bottom left). 14
13 The schematic geometries of bilayer (a) $MoSe_2/WS_2$ at a twisted angle of 6.1° , and (b) WTe_2/MoS_2 at a twisted angle of 3.0° . These include the top view (top) and the side view (bottom) of geometries. 14
14 The orbital projecting density of states of bilayer $MoTe_2/WSe_2$ at a stacking angle of (a) 0° and (b) 60° . The corresponding influential orbitals of each element are shown. 15

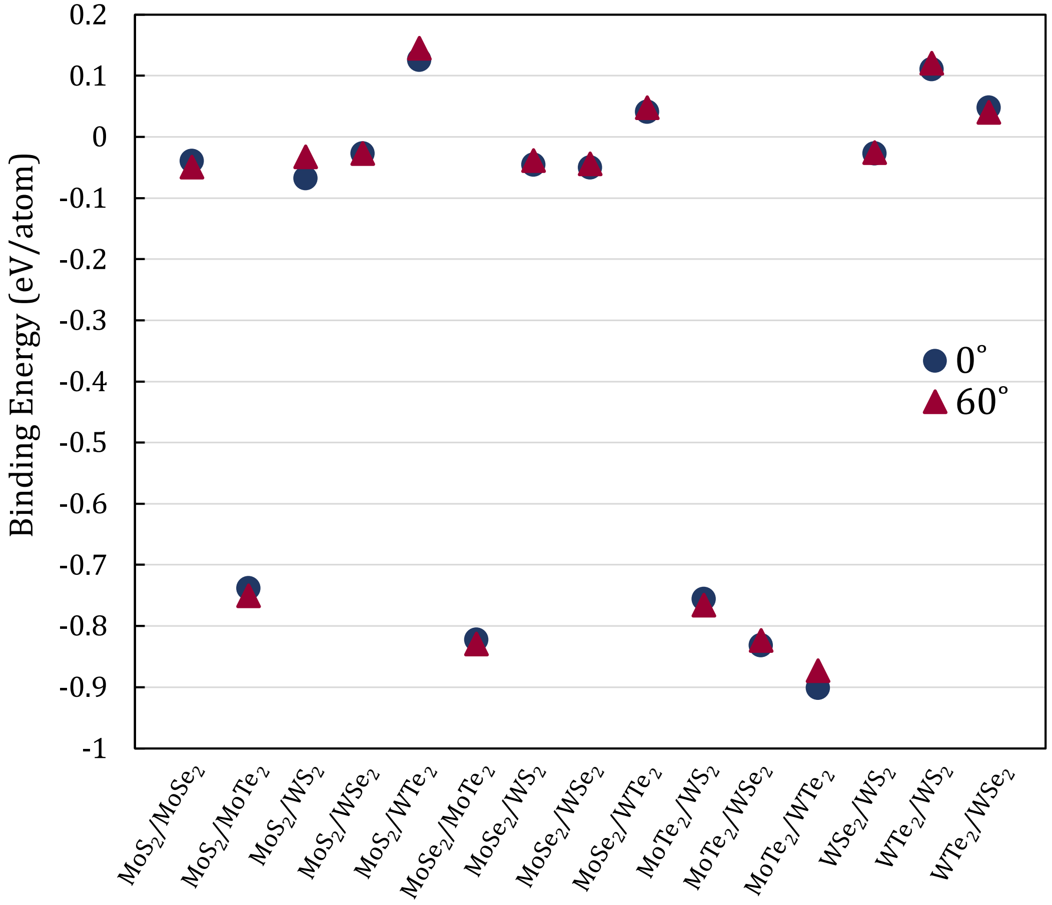

Figure S1. Binding energy are shown for 15 bilayer TMDCs heterostructures with two types of stacking, either 0 or 60 degrees of shifting.

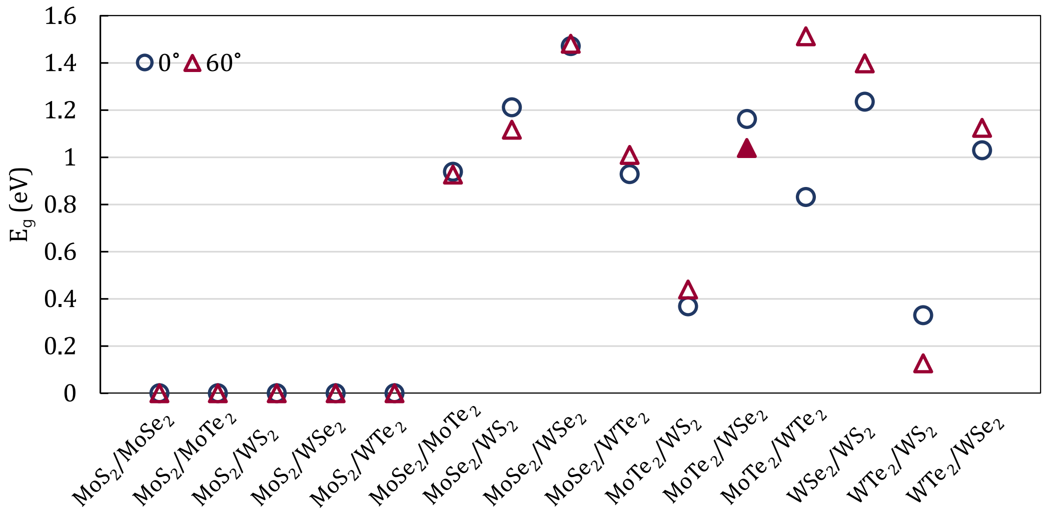

Figure S2. The E_g of 15 bilayer TMDCs heterostructures compared with two types of stacking, either 0 or 60 degrees of shifting. Hollow signs represent indirect E_g while filled sign stands for direct E_g. Compared to this plot where 1T-MoS_2 was applied, 1H-MoS_2 was applied in Figure 2.

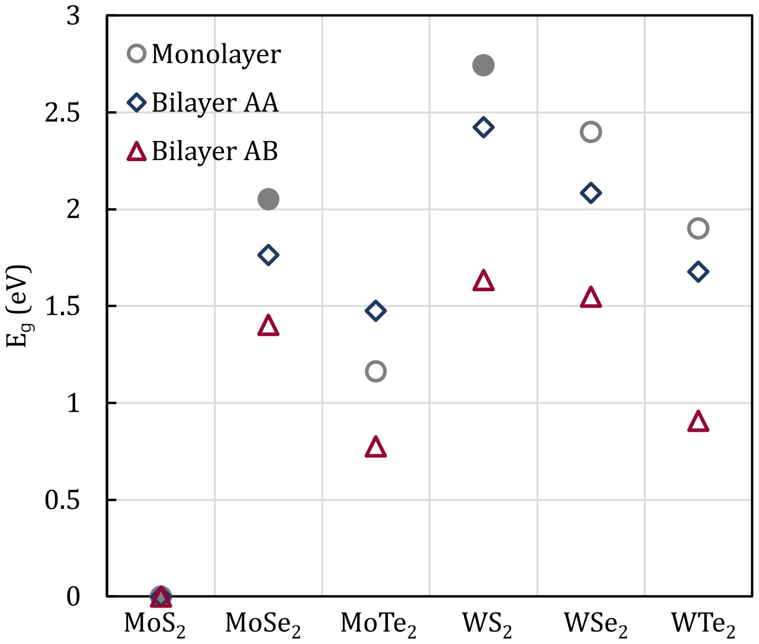

Figure S3. The E_g are shown of 6 fully optimized TMDCs in forms of monolayer, A-A stacking bilayer, and A-B stacking bilayer. The MoS_2 in this plot belongs to 1T instead of 1H. Hollow signs represent indirect E_g while filled signs stand for direct E_g.

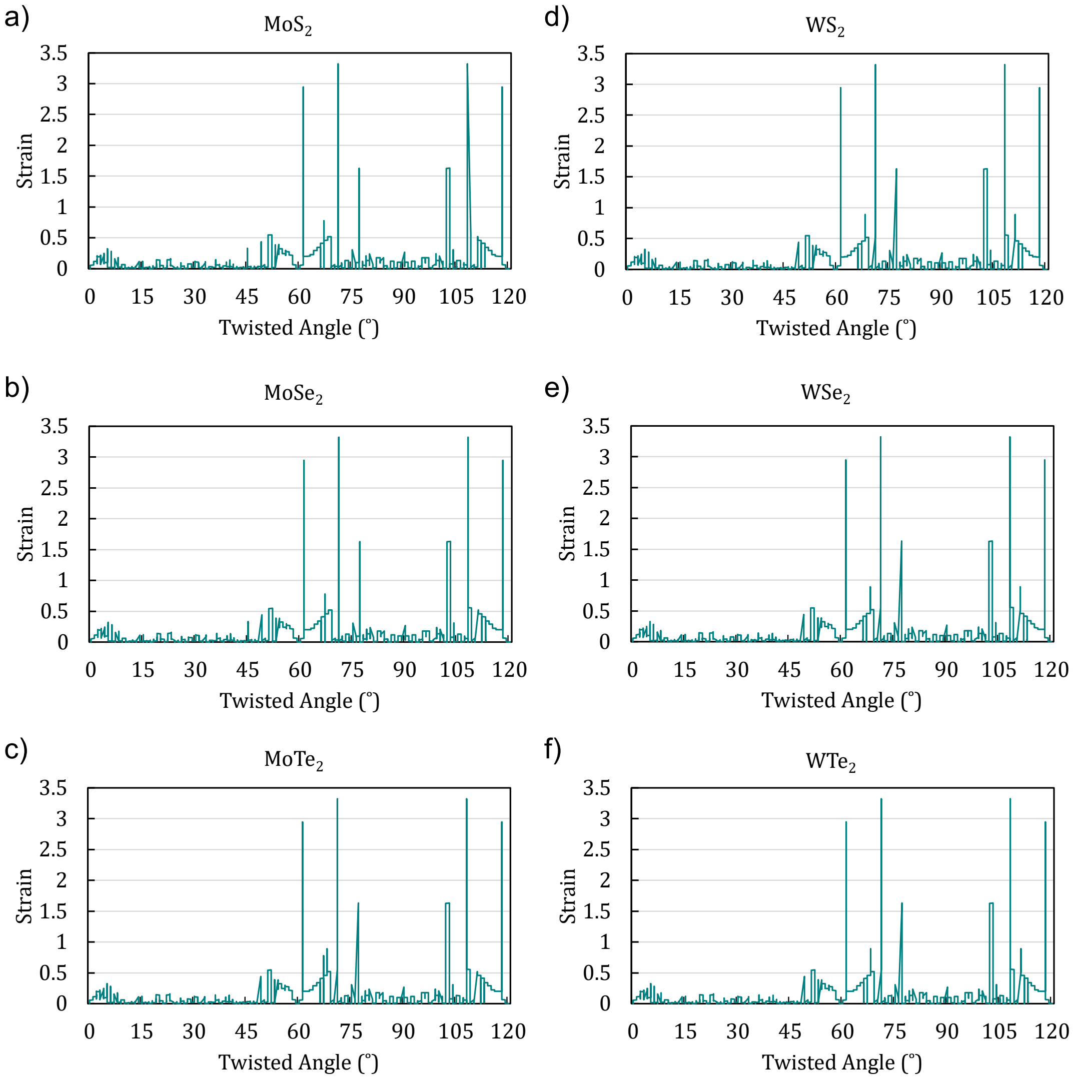

Figure S4. The overall fluctuation of strain according to the twisted angles of 1H bilayers are shown for the cases of (a) MoS_2, (b) $MoSe_2$, (c) $MoTe_2$, (d) WS_2, (e) WSe_2, and (f) WTe_2.

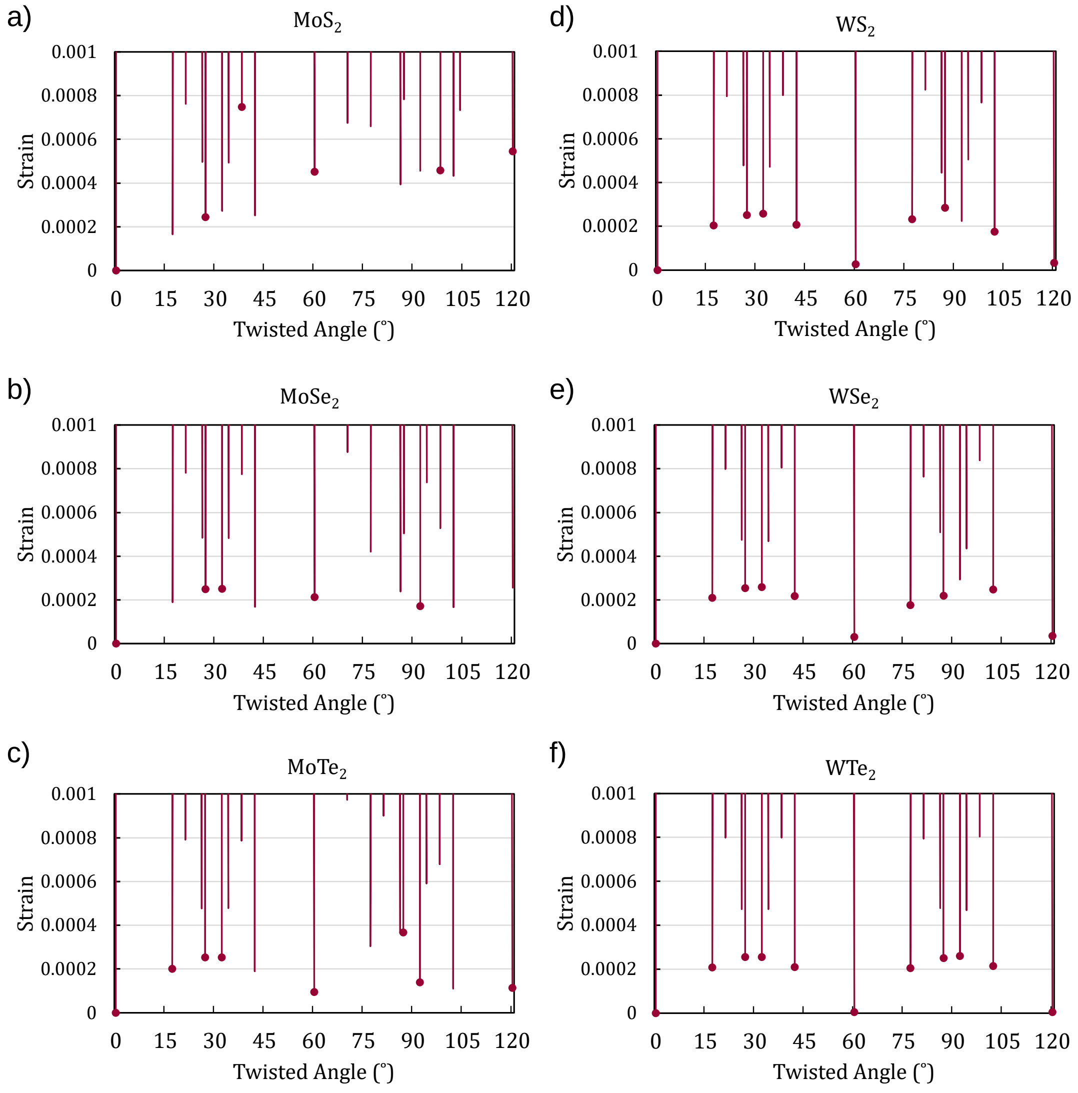

Figure S5. The zoom-in fluctuation of strain below 0.001 according to the twisted angles of 1H bilayers in the cases of (a) MoS_2, (b) $MoSe_2$, (c) $MoTe_2$, (d) WS_2, (e) WSe_2, and (f) WTe_2. The low-strain twisted TMDCs bilayers being symmetrized and further computed in this work are dotted in the plots.

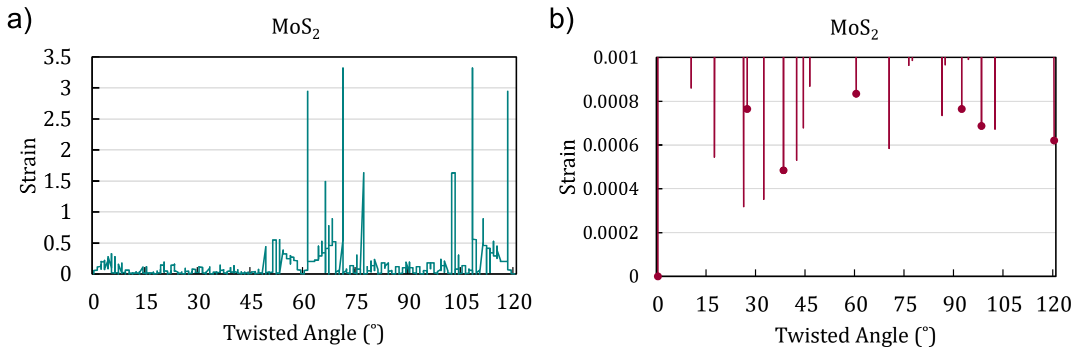

Figure S6. (a) The overall fluctuation of strain according to the twisted angles of bilayers in the cases of 1T-MoS_2. (b) The zoom-in fluctuation of strain below 0.001 according to the twisted angles of bilayers in the cases of 1T-MoS_2. The low-strain twisted TMDCs bilayers being symmetrized and further computed in this work are dotted in the plots.

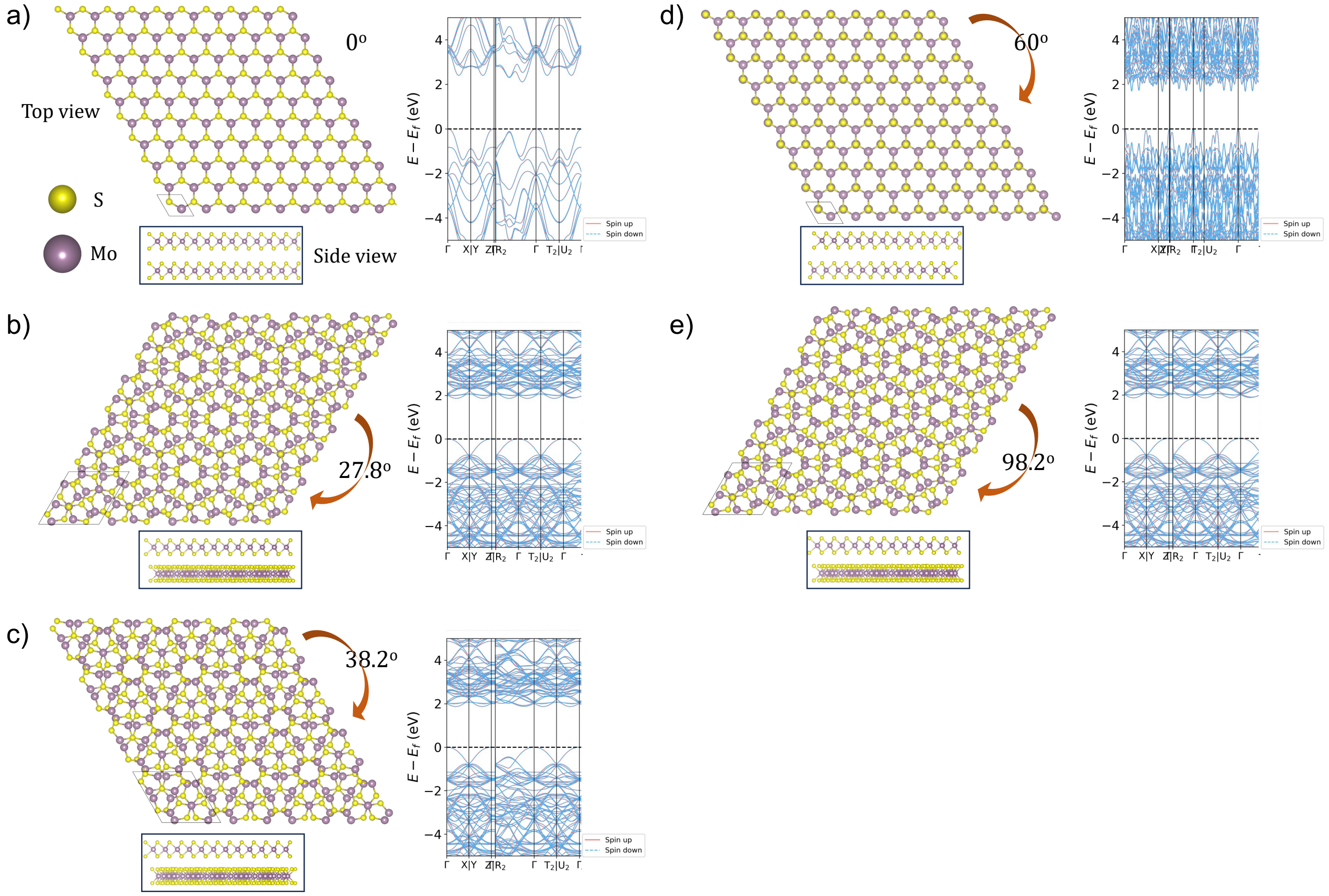

Figure S7. The schematic geometries and band structures of bilayer 1H-MoS_2 with the twisted angles of (a) 0° , (b) 27.8° , (c) 38.2° , (d) 60° , and (e) 98.2° . These include the top view and the side view of geometries (bottom left).

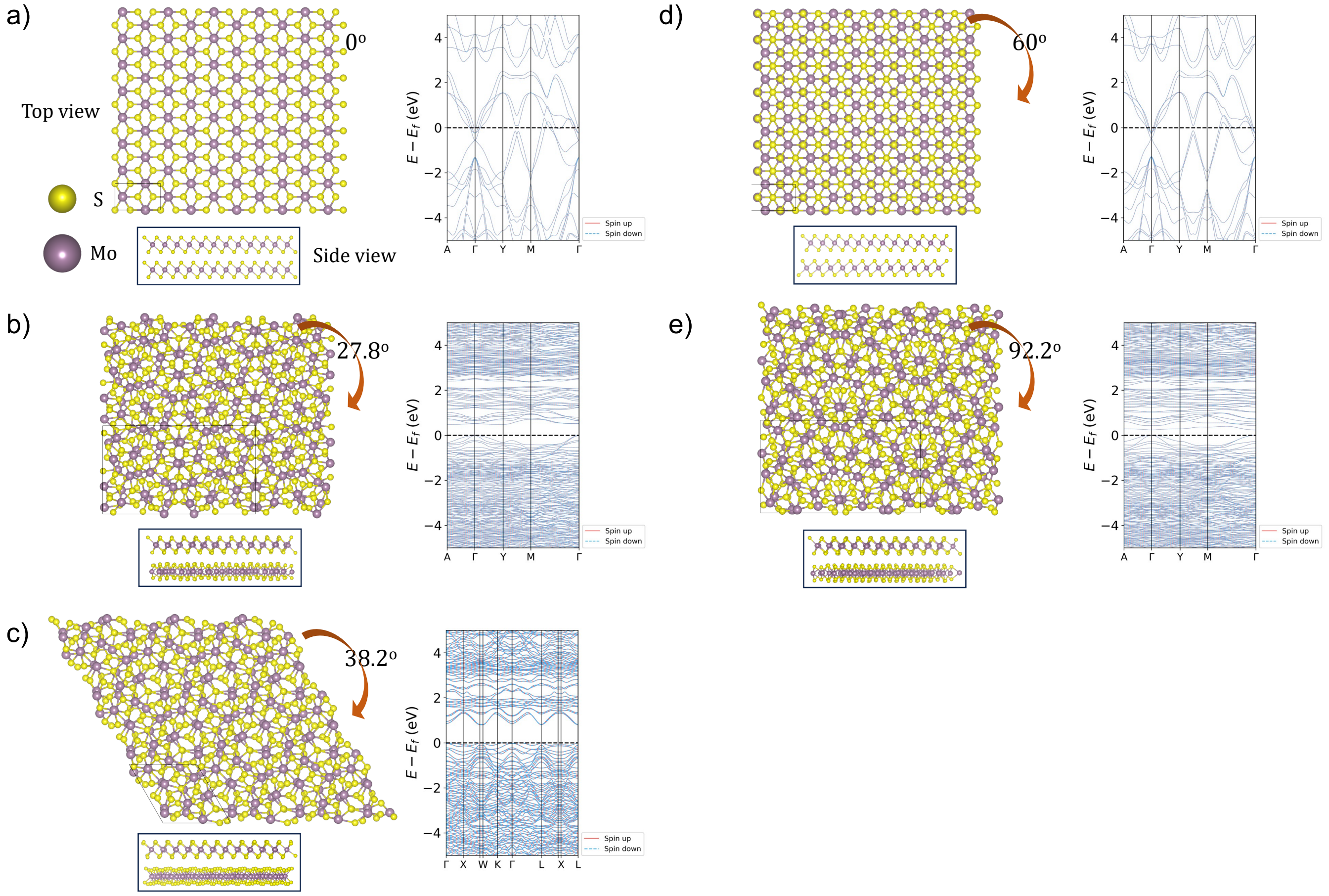

Figure S8. The schematic geometries and band structures of bilayer 1T-MoS_2 with the twisted angles of (a) 0° , (b) 27.8° , (c) 38.2° , (d) 60° , and (e) 92.2° . These include the top view and the side view of geometries (bottom left).

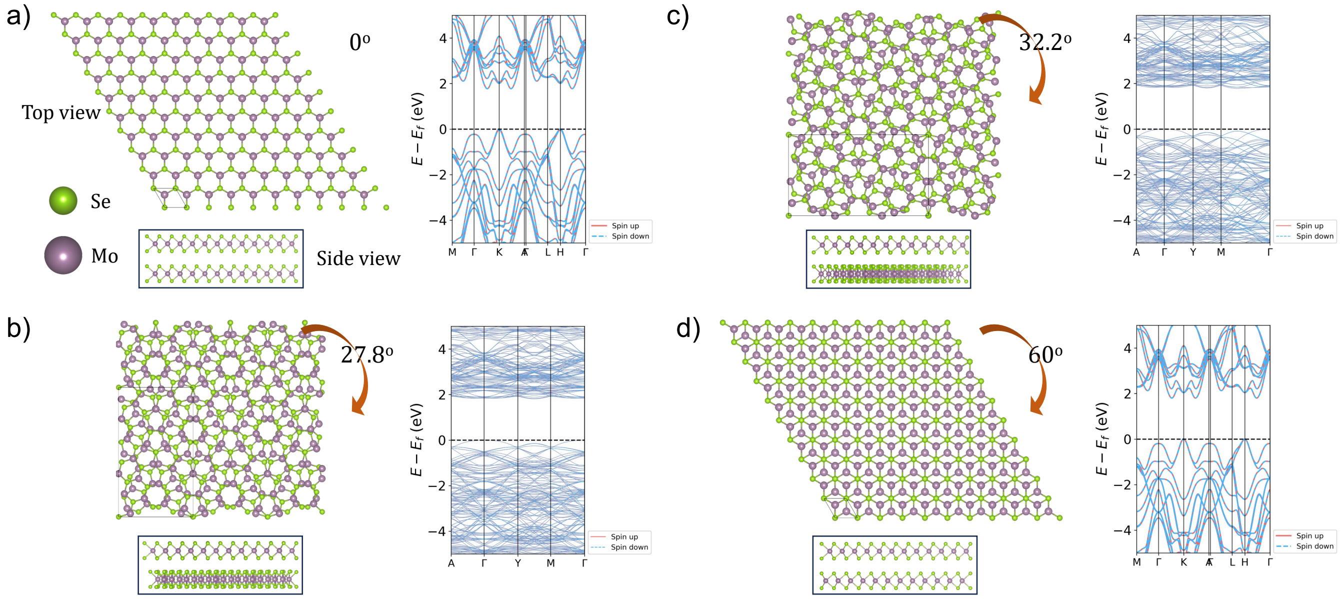

Figure S9. The schematic geometries and band structures of bilayer $MoSe_2$ with the twisted angles of (a) 0° , (b) 27.8° , (c) 32.2° , and (d) 60° . These include the top view and the side view of geometries (bottom left).

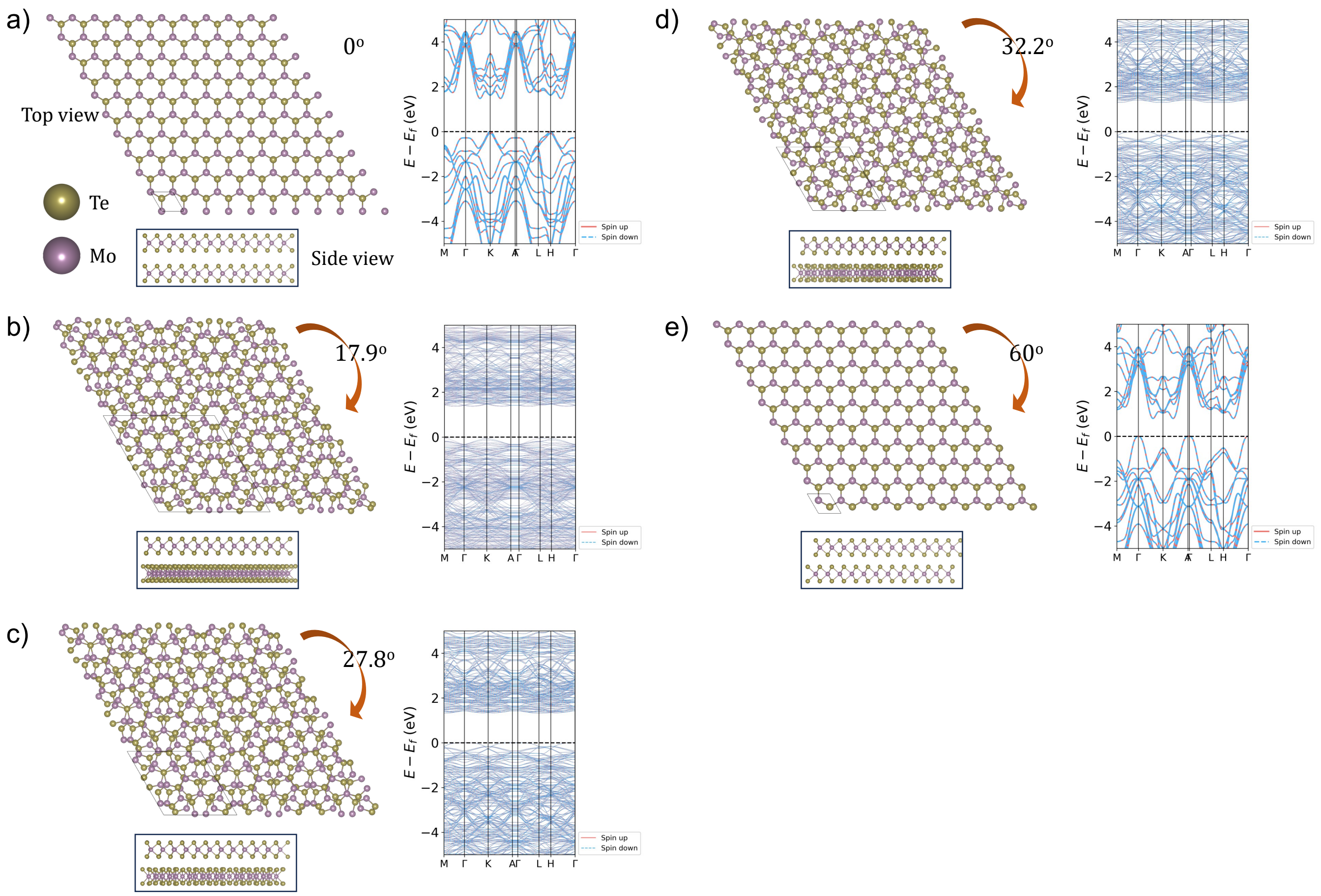

Figure S10. The schematic geometries and band structures of bilayer $MoTe_2$ with the twisted angles of (a) 0° , (b) 17.9° , (c) 27.8° , (d) 32.2° , and (e) 60° . These include the top view and the side view of geometries (bottom left).

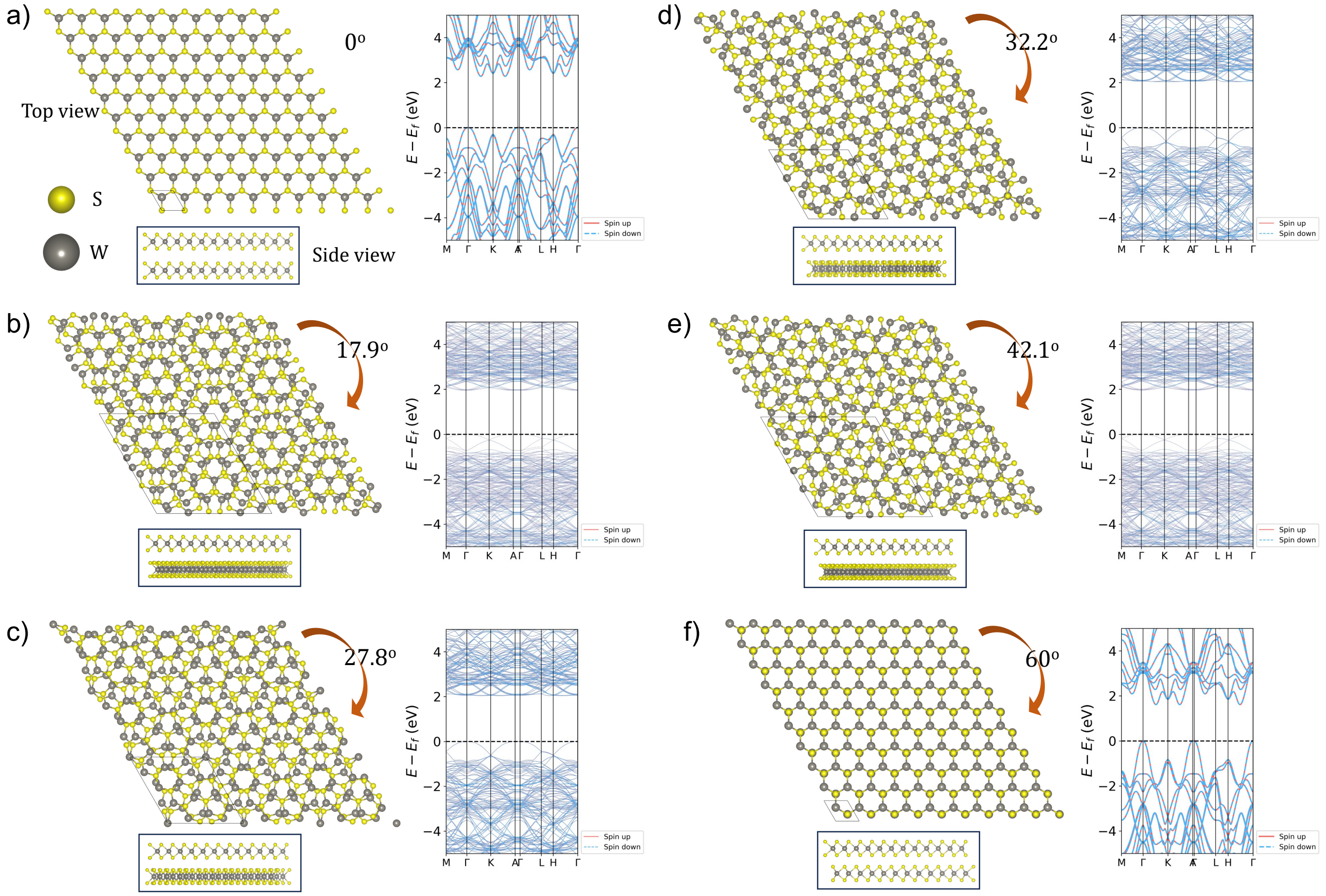

Figure S11. The schematic geometries and band structures of bilayer WS_2 with the twisted angles of (a) 0° , (b) 17.9° , (c) 27.8° , (d) 32.2° , (e) 42.1° , and (f) 60° . These include the top view and the side view of geometries (bottom left).

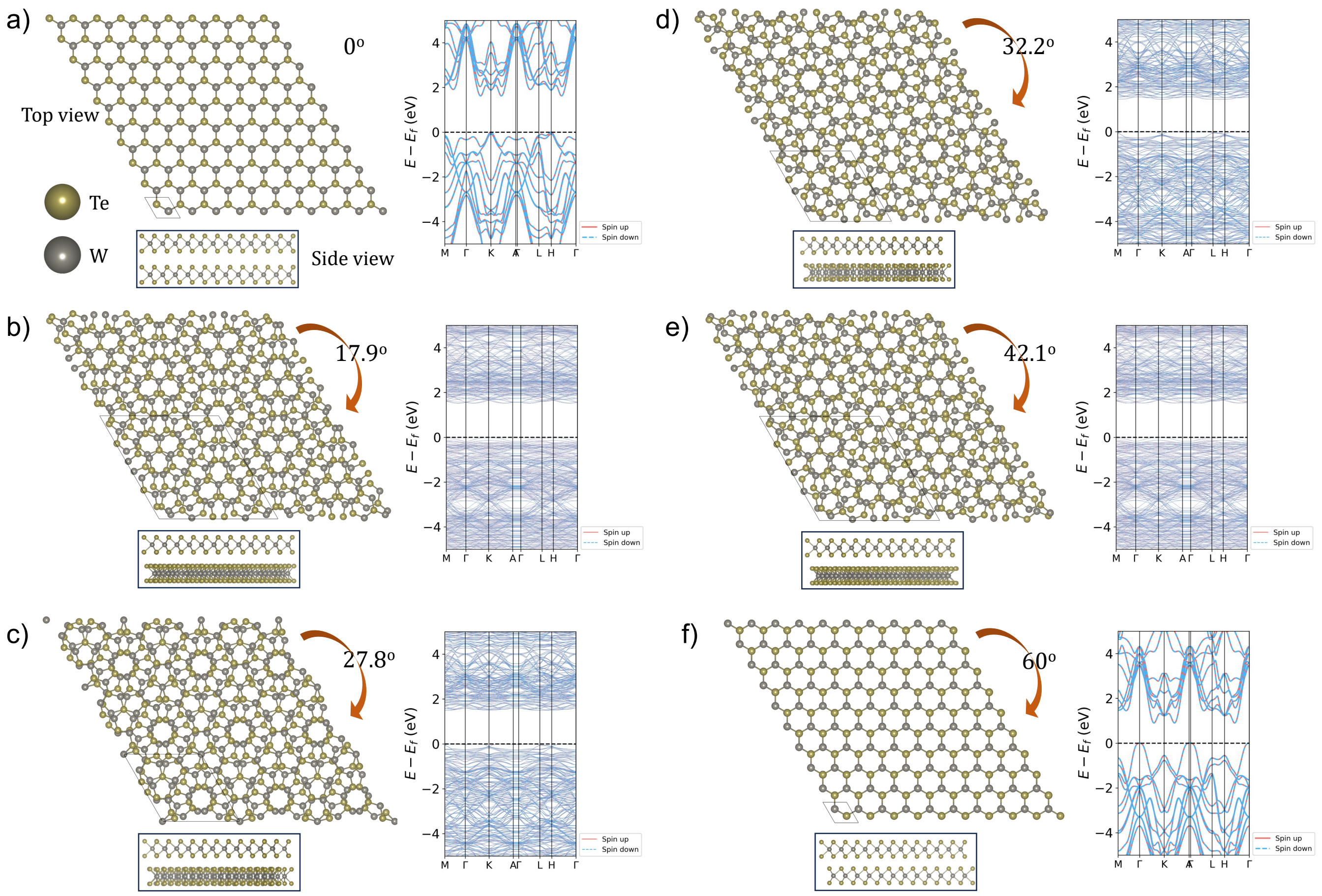

Figure S12. The schematic geometries and band structures of bilayer WTe_2 with the twisted angles of (a) 0° , (b) 17.9° , (c) 27.8° , (d) 32.2° , (e) 42.1° , and (f) 60° . These include the top view and the side view of geometries (bottom left).

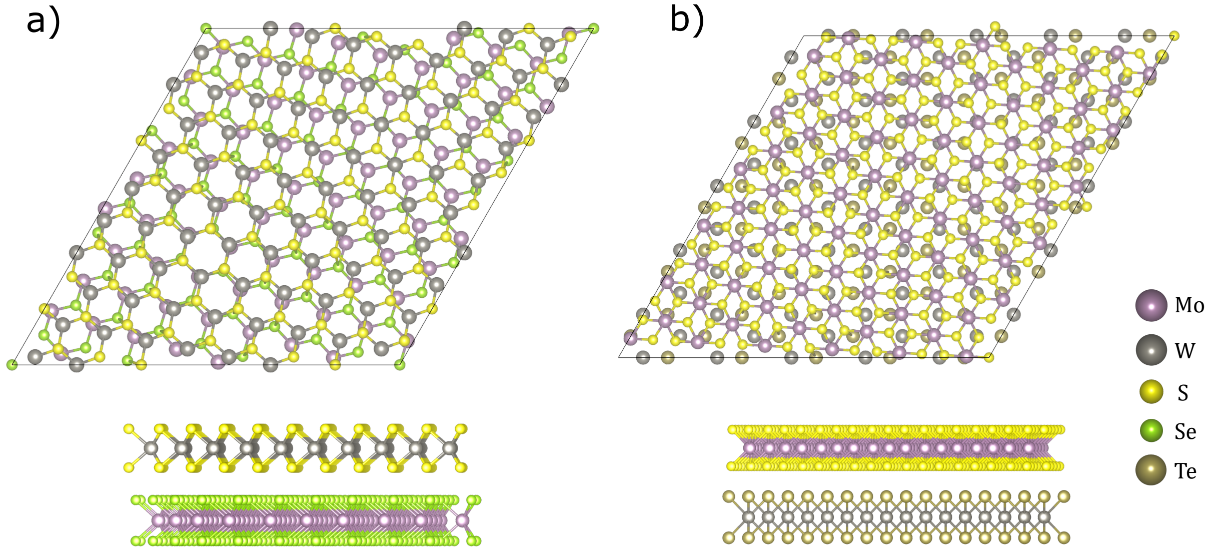

Figure S13. The schematic geometries of bilayer (a) $MoSe_2/WS_2$ at a twisted angle of 6.1° , and (b) WTe_2/MoS_2 at a twisted angle of 3.0° . These include the top view (top) and the side view (bottom) of geometries.

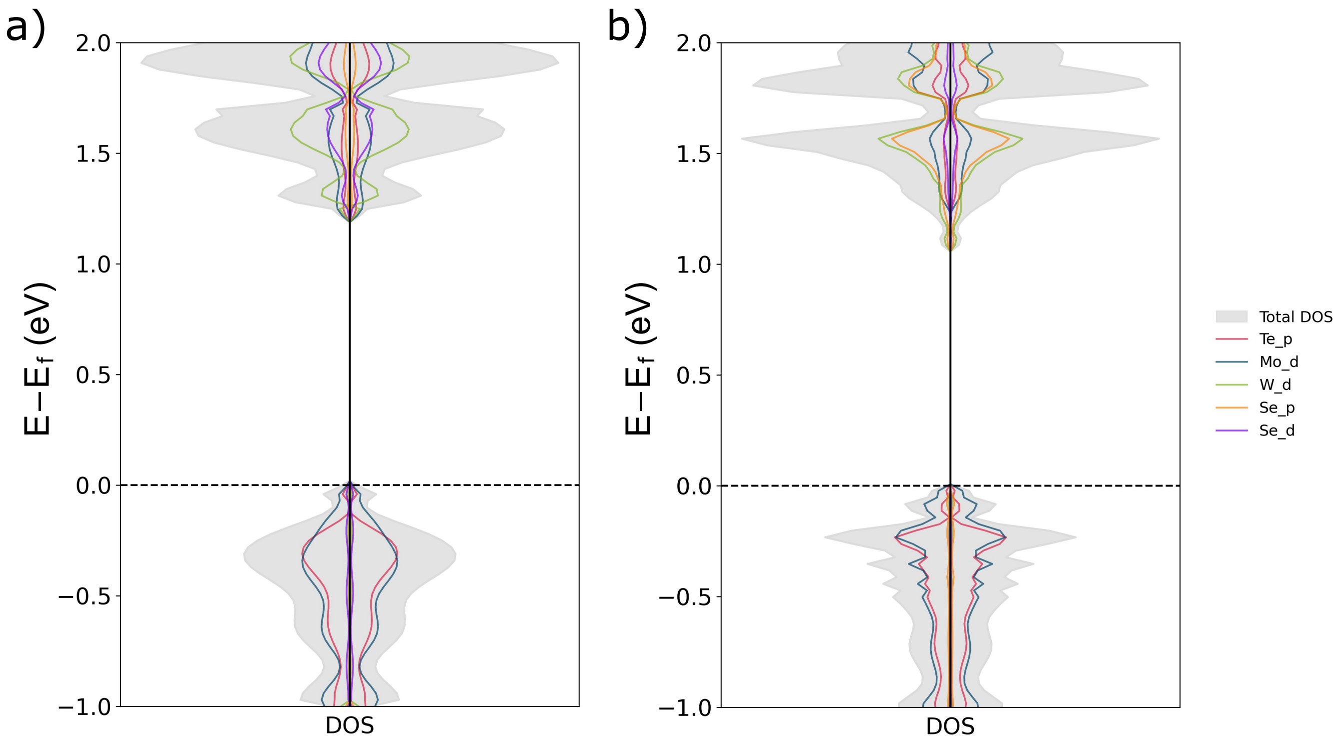

Figure S14. The orbital projecting density of states of bilayer $MoTe_2/WSe_2$ at a stacking angle of (a) 0° and (b) 60° . The corresponding influential orbitals of each element are shown.

I. OPTIMIZED GEOMETRIES OF RELEVANT BILAYERS

1. 1T-MoS_2/1T-MoS_2-0

```
_cell_length_a                       5.352046
_cell_length_b                       3.090760
_cell_length_c                       40.000000
_cell_angle_alpha                    90.000000
_cell_angle_beta                     90.000000
_cell_angle_gamma                    90.000000
_cell_volume                         661.675631
_space_group_name_H-M_alt            'C m'
_space_group_IT_number               8

loop_
_space_group_symop_operation_xyz
   'x, y, z'
   'x, -y, z'
   'x+1/2, y+1/2, z'
   'x+1/2, -y+1/2, z'

loop_
   _atom_site_label
   _atom_site_occupancy
   _atom_site_fract_x
   _atom_site_fract_y
   _atom_site_fract_z
   _atom_site_adp_type
   _atom_site_B_iso_or_equiv
   _atom_site_type_symbol
   Mo1        1.0  -0.3328953866529000 0.0000000000000000 0.2913286022584   Biso  1.000000 Mo
   S1         1.0  0.0005439707940378 0.0000000000000000 0.3305239829714   Biso  1.000000 S
   S2         1.0  0.3338460841092000 -0.0000000000000001 0.2517302238301   Biso  1.000000 S
   Mo2        1.0  -0.3337772564703000 0.0000000000000000 0.4271304508988   Biso  1.000000 Mo
   S3         1.0  -0.0004958675048382 0.0000000000000000 0.4667224247912   Biso  1.000000 S
   S4         1.0  0.3327884557248000 0.0000000000000000 0.3879443152501   Biso  1.000000 S
```

2. 1T-MoS_2/1T-MoS_2-27.8

```
_cell_length_a                       19.302418
_cell_length_b                       11.140800
_cell_length_c                       40.000000
_cell_angle_alpha                    90.000000
_cell_angle_beta                     90.000000
_cell_angle_gamma                    90.000000
_cell_volume                         8601.775397
_space_group_name_H-M_alt            'C 2'
_space_group_IT_number               5

loop_
_space_group_symop_operation_xyz
   'x, y, z'
   '-x, y, -z'
   'x+1/2, y+1/2, z'
   '-x+1/2, y+1/2, -z'

loop_
   _atom_site_label
   _atom_site_occupancy
   _atom_site_fract_x
   _atom_site_fract_y
   _atom_site_fract_z
   _atom_site_adp_type
   _atom_site_B_iso_or_equiv
   _atom_site_type_symbol
   Mo1        1.0 0.26769003994510 -0.03492261502805 -0.42725501294640 Biso  1.000000 Mo
   Mo2        1.0 0.12283849436530 0.13233190331450 -0.42583159080840 Biso  1.000000 Mo
   Mo3        1.0 -0.22473530305390 -0.27552135456860 -0.42696244406340 Biso  1.000000 Mo
   Mo4        1.0 0.17821188596560 0.43189301395210 -0.42715717210860 Biso  1.000000 Mo
   Mo5        1.0 0.07079545754586 -0.38034619048050 -0.42696108630340 Biso  1.000000 Mo
   Mo6        1.0 -0.03963481793324 -0.19460376820040 -0.42503083259570 Biso  1.000000 Mo
   Mo7        1.0 -0.18907005767560 -0.02903630205396 -0.42472780809750 Biso  1.000000 Mo
   Mo8        1.0 0.20208130999150 -0.33549941968600 -0.42372753536730 Biso  1.000000 Mo
   Mo9        1.0 0.09207429586662 -0.13873489741280 -0.42366030712930 Biso  1.000000 Mo
   Mo10       1.0 -0.01166056820141 0.07380310958695 -0.42549492129520 Biso  1.000000 Mo
   Mo11       1.0 -0.12239460757940 0.27014751015540 -0.42638530916840 Biso  1.000000 Mo
   Mo12       1.0 0.00990061037005 0.31222263968630 -0.42362417244640 Biso  1.000000 Mo
   Mo13       1.0 -0.09801889488629 -0.49888779698010 -0.42357730318970 Biso  1.000000 Mo
   S1         1.0 0.31717162387790 0.07380244774436 -0.38532498223200 Biso  1.000000 S
   S2         1.0 0.19427535839880 0.26177847355840 -0.38752759927690 Biso  1.000000 S
   S3         1.0 -0.14499496868840 -0.17156458866590 -0.38908650359200 Biso  1.000000 S
   S4         1.0 -0.26341492621590 0.02780708914095 -0.38210095765110 Biso  1.000000 S
   S5         1.0 0.12416632869350 -0.27741263299400 -0.38219536171500 Biso  1.000000 S
   S6         1.0 0.00974653808535 -0.08320965272561 -0.38303987182000 Biso  1.000000 S
   S7         1.0 -0.11272149769620 0.09798680799691 -0.39062752489670 Biso  1.000000 S
   S8         1.0 0.27648140158040 -0.20305691727650 -0.39112295983270 Biso  1.000000 S
   S9         1.0 0.16722000877910 -0.01063921274083 -0.38944568158430 Biso  1.000000 S
   S10        1.0 0.04532207681493 0.17760166579330 -0.38236583484270 Biso  1.000000 S
   S11        1.0 -0.06853968733088 0.36939281000430 -0.38097621736280 Biso  1.000000 S
   S12        1.0 0.08173292890466 0.44921496942480 -0.39022542731880 Biso  1.000000 S
   S13        1.0 -0.02840166185163 -0.36096446613860 -0.38959179087260 Biso  1.000000 S
   S14        1.0 -0.15550312547380 0.40574155419160 -0.46857024771520 Biso  1.000000 S
   S15        1.0 0.22051516743180 0.09895800010551 -0.46563509462500 Biso  1.000000 S
   S16        1.0 -0.11892417510460 -0.31962881156840 -0.45725688178610 Biso  1.000000 S
   S17        1.0 -0.24258381256080 -0.13023106629890 -0.46864959511400 Biso  1.000000 S
   S18        1.0 0.14890281933660 -0.43263864013580 -0.46927407656720 Biso  1.000000 S
   S19        1.0 0.03703861071719 -0.24151316058860 -0.46774598529570 Biso  1.000000 S
   S20        1.0 -0.08727666730831 -0.05379680757444 -0.46039446576100 Biso  1.000000 S
   S21        1.0 -0.19719010857370 0.14204901123310 -0.45946903911440 Biso  1.000000 S
   S22        1.0 0.19036298071410 -0.16492077599710 -0.45968487265160 Biso  1.000000 S
   S23        1.0 0.06924148450767 0.01906285085128 -0.46589697438010 Biso  1.000000 S
   S24        1.0 -0.04556620405995 0.20907112236030 -0.46782838038070 Biso  1.000000 S
   S25        1.0 0.10664483701100 0.29832735223910 -0.46102158575200 Biso  1.000000 S
   S26        1.0 -0.00330704194502 0.48578674577580 -0.46010599495480 Biso  1.000000 S
```

3. 1T-MoS_2/1T-MoS_2-38.2

```
_cell_length_a                         8.175121
_cell_length_b                         8.176699
_cell_length_c                         40.000000
_cell_angle_alpha                      90.000000
_cell_angle_beta                       90.000000
_cell_angle_gamma                      119.988647
_cell_volume                           2315.861020
_space_group_name_H-M_alt              'P 1'
_space_group_IT_number                 1

loop_
_space_group_symop_operation_xyz
   'x, y, z'

loop_
   _atom_site_label
   _atom_site_occupancy
   _atom_site_fract_x
   _atom_site_fract_y
   _atom_site_fract_z
   _atom_site_adp_type
   _atom_site_B_iso_or_equiv
   _atom_site_type_symbol
   Mo1        1.0 -0.16822817983360 0.44018999161590 0.28568077163270  Biso  1.000000 Mo
   Mo2        1.0 0.00127653873115 -0.13409055758370 0.28406241236090  Biso  1.000000 Mo
   Mo3        1.0 0.14514147109670 0.24530359810160 0.28681056604310  Biso  1.000000 Mo
   Mo4        1.0 0.32929437482980 -0.31383205167870 0.28520026339880  Biso  1.000000 Mo
   Mo5        1.0 0.43902846768930 0.17649897127550 0.28363830765790  Biso  1.000000 Mo
   Mo6        1.0 -0.41260260718880 -0.42984799624610 0.28430613443290  Biso  1.000000 Mo
   Mo7        1.0 -0.27076805001470 -0.04665608240057 0.28684144945180  Biso  1.000000 Mo
   S1         1.0 -0.18549872979610 -0.38119863205200 0.32678250337460  Biso  1.000000 S
   S2         1.0 -0.03262233712827 0.03618486227771 0.32814356498470  Biso  1.000000 S
   S3         1.0 0.09022272143023 0.46697953746460 0.32027403164260  Biso  1.000000 S
   S4         1.0 0.25183456258720 -0.10721589480040 0.31801209266270  Biso  1.000000 S
   S5         1.0 0.39130829821620 0.33731155166260 0.32610498259490  Biso  1.000000 S
   S6         1.0 -0.46149283788260 -0.25302189207380 0.32836081229020  Biso  1.000000 S
   S7         1.0 -0.33122323489580 0.17176598967640 0.31918366763110  Biso  1.000000 S
   S8         1.0 0.06724695027031 -0.34436272519000 0.24973270053810  Biso  1.000000 S
   S9         1.0 0.19456181918180 0.08250035816543 0.24328136573620  Biso  1.000000 S
   S10        1.0 0.33987717940430 -0.49648488405950 0.24446973059490  Biso  1.000000 S
   S11        1.0 0.49264161418470 -0.05000032355244 0.25056997205260  Biso  1.000000 S
   S12        1.0 -0.37314453795370 0.38043071873410 0.24159678625930  Biso  1.000000 S
   S13        1.0 -0.22829252032030 -0.20462509977980 0.24238375351000  Biso  1.000000 S
   S14        1.0 -0.09181198736999 0.23227240365860 0.25159186605280  Biso  1.000000 S
   Mo8        1.0 0.35311819092690 -0.33705114559560 0.43323799136470  Biso  1.000000 Mo
   Mo9        1.0 -0.15653656765360 -0.22720449381840 0.43484250089740  Biso  1.000000 Mo
   Mo10       1.0 -0.46732823853020 0.33469987262830 0.43438781309350  Biso  1.000000 Mo
   Mo11       1.0 -0.08762324807338 0.47907748985340 0.43165180529190  Biso  1.000000 Mo
   Mo12       1.0 0.10724026156730 0.16556096523290 0.43277508318050  Biso  1.000000 Mo
   Mo13       1.0 0.23702285997600 -0.07902916379658 0.43415656265440  Biso  1.000000 Mo
   Mo14       1.0 -0.37972377788110 0.06296652005898 0.43163315680550  Biso  1.000000 Mo
   S15        1.0 0.17046642700590 -0.32645170159360 0.47398412521260  Biso  1.000000 S
   S16        1.0 -0.38311577139360 -0.17365061242670 0.46790286805390  Biso  1.000000 S
   S17        1.0 0.32262237564240 0.40097112296030 0.46872047662110  Biso  1.000000 S
   S18        1.0 -0.25061820342510 -0.47177099416540 0.47516905017030  Biso  1.000000 S
   S19        1.0 -0.10069091233760 0.24199751452570 0.46685103244760  Biso  1.000000 S
   S20        1.0 0.04738977128052 -0.03934083473567 0.47687066655480  Biso  1.000000 S
   S21        1.0 0.46226481904570 0.10535521728490 0.47609131592930  Biso  1.000000 S
   S22        1.0 0.41403409008560 -0.12772127611070 0.39009835855720  Biso  1.000000 S
   S23        1.0 -0.16136643594030 0.00243592517707 0.39930384905750  Biso  1.000000 S
   S24        1.0 -0.44020117711850 -0.41454575241870 0.40045795624330  Biso  1.000000 S
   S25        1.0 0.00411172837586 -0.27492469007660 0.39235461302190  Biso  1.000000 S
   S26        1.0 0.13399976546720 0.42398913281870 0.39815575471630  Biso  1.000000 S
   S27        1.0 0.28568019503620 0.14799807155470 0.39166052214230  Biso  1.000000 S
   S28        1.0 -0.29669512729420 0.30107698942770 0.39032676308100  Biso  1.000000 S
```

4. 1T-MoS_2/1T-MoS_2-60

```
_cell_length_a                      5.353871
_cell_length_b                      3.089693
_cell_length_c                      40.000000
_cell_angle_alpha                   90.000000
_cell_angle_beta                    90.000000
_cell_angle_gamma                   90.000000
_cell_volume                        661.672607
_space_group_name_H-M_alt           'C m'
_space_group_IT_number              8

loop_
_space_group_symop_operation_xyz
   'x, y, z'
   'x, -y, z'
   'x+1/2, y+1/2, z'
   'x+1/2, -y+1/2, z'

loop_
   _atom_site_label
   _atom_site_occupancy
   _atom_site_fract_x
   _atom_site_fract_y
   _atom_site_fract_z
   _atom_site_adp_type
   _atom_site_B_iso_or_equiv
   _atom_site_type_symbol
   Mo1        1.0    0.16658900351830000 -0.50000000000000000 0.28979883014050000    Biso  1.000000 Mo
   S1         1.0    -0.00015240494588770 0.00000000000000000 0.32916019830630000    Biso  1.000000 S
   S2         1.0    -0.16664223180140000 -0.50000000000000000 0.25029190352030000    Biso  1.000000 S
   Mo2        1.0    0.33328673799720000 0.00000000000000001 0.42869059243750000    Biso  1.000000 Mo
   S3         1.0    -0.00003407942968994 0.00000000000000000 0.46819419787490000    Biso  1.000000 S
   S4         1.0    0.16645297466140000 -0.50000000000000000 0.38924427772050000    Biso  1.000000 S
```

5. 1T-MoS_2/1T-MoS_2-92.2

```
_cell_length_a                         19.296396
_cell_length_b                         11.144260
_cell_length_c                         40.000000
_cell_angle_alpha                      90.000000
_cell_angle_beta                       90.000000
_cell_angle_gamma                      90.000000
_cell_volume                           8601.762591
_space_group_name_H-M_alt              'C 2'
_space_group_IT_number                 5

loop_
_space_group_symop_operation_xyz
   'x, y, z'
   '-x, y, -z'
   'x+1/2, y+1/2, z'
   '-x+1/2, y+1/2, -z'

loop_
   _atom_site_label
   _atom_site_occupancy
   _atom_site_fract_x
   _atom_site_fract_y
   _atom_site_fract_z
   _atom_site_adp_type
   _atom_site_B_iso_or_equiv
   _atom_site_type_symbol
   Mo1        1.0 -0.08703042530211 0.17925637314640 -0.42330818144230  Biso  1.000000 Mo
   Mo2        1.0 0.06100994868191 0.10952776523930 -0.42359940514620  Biso  1.000000 Mo
   Mo3        1.0 0.18385752326480 0.35191631210400 -0.42500552246180  Biso  1.000000 Mo
   Mo4        1.0 -0.19886615617020 0.03241150268002 -0.42422821104680  Biso  1.000000 Mo
   Mo5        1.0 0.23067562308740 0.03335538016184 -0.42520568591650  Biso  1.000000 Mo
   Mo6        1.0 -0.15980698270640 -0.29089987695150 -0.42456252991510  Biso  1.000000 Mo
   Mo7        1.0 -0.04274027394310 -0.04164336682599 -0.42688657500830  Biso  1.000000 Mo
   Mo8        1.0 -0.11695802018220 0.48589218663390 -0.42532335420250  Biso  1.000000 Mo
   Mo9        1.0 -0.01037654508472 -0.35818027759870 -0.42380609878500  Biso  1.000000 Mo
   Mo10       1.0 0.10990079782980 -0.11025015188760 -0.42721743130160  Biso  1.000000 Mo
   Mo11       1.0 0.03367754183591 0.42211757885470 -0.42685606443660  Biso  1.000000 Mo
   Mo12       1.0 0.13908885916330 -0.42667799072840 -0.42335373671270  Biso  1.000000 Mo
   Mo13       1.0 -0.22405581163660 0.28840561659760 -0.42425171356010  Biso  1.000000 Mo
   S1         1.0 -0.02015336881475 0.08599668854522 -0.38122356864600  Biso  1.000000 S
   S2         1.0 0.13742018532740 0.01756925057903 -0.38428907170280  Biso  1.000000 S
   S3         1.0 0.24714736393800 0.20819074942650 -0.38849004835900  Biso  1.000000 S
   S4         1.0 -0.13463263150290 -0.11145806606130 -0.39126651875080  Biso  1.000000 S
   S5         1.0 0.29361593246080 -0.06214731484464 -0.38363947773970  Biso  1.000000 S
   S6         1.0 -0.09462920309263 -0.37341159604160 -0.38184842238100  Biso  1.000000 S
   S7         1.0 0.02002100215413 -0.18005828023760 -0.39146830944710  Biso  1.000000 S
   S8         1.0 -0.05455626092182 0.35336894255510 -0.38936567135410  Biso  1.000000 S
   S9         1.0 0.05673308557474 -0.44553325833670 -0.38153082878470  Biso  1.000000 S
   S10        1.0 0.17241031610760 -0.24972666519510 -0.39193053849430  Biso  1.000000 S
   S11        1.0 0.09448977748624 0.28281090781640 -0.38985031106020  Biso  1.000000 S
   S12        1.0 -0.28820049730540 -0.01550639715714 -0.38287940792850  Biso  1.000000 S
   S13        1.0 -0.17202128471440 0.16928601655140 -0.38179851605320  Biso  1.000000 S
   S14        1.0 -0.11427245708630 0.04699403995219 -0.46710369712070  Biso  1.000000 S
   S15        1.0 0.04232291701704 -0.02499130961462 -0.46860721210000  Biso  1.000000 S
   S16        1.0 0.16078421557430 0.17048695639170 -0.45771438973670  Biso  1.000000 S
   S17        1.0 -0.22202671418410 -0.14584577395160 -0.45961997513320  Biso  1.000000 S
   S18        1.0 0.20162106058020 -0.10903484674090 -0.46518014524700  Biso  1.000000 S
   S19        1.0 -0.18559034615970 -0.42388915007110 -0.46720260512380  Biso  1.000000 S
   S20        1.0 -0.07116092955136 -0.21929279047380 -0.46033835030000  Biso  1.000000 S
   S21        1.0 -0.14102293152840 0.31364722542030 -0.46357377839720  Biso  1.000000 S
   S22        1.0 -0.03445454317301 -0.48970569577910 -0.46794107651800  Biso  1.000000 S
   S23        1.0 0.08138233037122 -0.29057283605790 -0.46032147034620  Biso  1.000000 S
   S24        1.0 0.00586793324932 0.24390683762670 -0.46022064666230  Biso  1.000000 S
   S25        1.0 0.12090414212180 0.43852242184920 -0.46782231574000  Biso  1.000000 S
   S26        1.0 -0.26565437206990 0.13412289242400 -0.46184889190770  Biso  1.000000 S
```

6. 1T-MoS_2/1T-MoS_2-98.2

```
_cell_length_a                          8.175148
_cell_length_b                          8.176700
_cell_length_c                          40.000000
_cell_angle_alpha                       90.000000
_cell_angle_beta                        90.000000
_cell_angle_gamma                       119.988846
_cell_volume                            2315.864228
_space_group_name_H-M_alt               'P 1'
_space_group_IT_number                  1

loop_
_space_group_symop_operation_xyz
   'x, y, z'

loop_
   _atom_site_label
   _atom_site_occupancy
   _atom_site_fract_x
   _atom_site_fract_y
   _atom_site_fract_z
   _atom_site_adp_type
   _atom_site_B_iso_or_equiv
   _atom_site_type_symbol
   Mo1         1.0  0.38645626798170 0.44421422166150 -0.28526111097870  Biso  1.000000 Mo
   Mo2         1.0  0.28745638245020 0.08298944265645 -0.28345238258180  Biso  1.000000 Mo
   Mo3         1.0  -0.44426507275270 -0.04780944178867 -0.28619240684110  Biso  1.000000 Mo
   Mo4         1.0  -0.20027754064850 -0.29936764344260 -0.28489261489550  Biso  1.000000 Mo
   Mo5         1.0  -0.32511150993880 0.32896545770890 -0.28488193886290  Biso  1.000000 Mo
   Mo6         1.0  0.00300813171298 0.21007037651790 -0.28352799959490  Biso  1.000000 Mo
   Mo7         1.0  0.06449908736993 -0.42923098523340 -0.28683407303200  Biso  1.000000 Mo
   S1          1.0  0.44923701630380 0.28414952702620 -0.32748584063900  Biso  1.000000 S
   S2          1.0  0.28823849058630 -0.15303582171130 -0.31897462566580  Biso  1.000000 S
   S3          1.0  -0.41355709387650 -0.27996593777330 -0.32104788869680  Biso  1.000000 S
   S4          1.0  -0.14074987773990 -0.44560257598040 -0.32841145463330  Biso  1.000000 S
   S5          1.0  -0.25595161152330 0.15154139640410 -0.32564536447760  Biso  1.000000 S
   S6          1.0  0.01070309545070 -0.01564505623813 -0.31914586038480  Biso  1.000000 S
   S7          1.0  0.16930405000450 0.42346329244280 -0.32592278327760  Biso  1.000000 S
   S8          1.0  0.20707007216370 0.23499580828790 -0.24163693171750  Biso  1.000000 S
   S9          1.0  0.06615043732555 -0.19889400692850 -0.25014518919790  Biso  1.000000 S
   S10         1.0  0.34973151568850 -0.33174070096440 -0.25262764352310  Biso  1.000000 S
   S11         1.0  -0.37495486959530 -0.49168648606580 -0.24259722475170  Biso  1.000000 S
   S12         1.0  0.49441511832430 0.09525332835835 -0.24214327810900  Biso  1.000000 S
   S13         1.0  -0.22498299935340 -0.06578054498349 -0.25043276362100  Biso  1.000000 S
   S14         1.0  -0.08841200447077 0.36464664955920 -0.24393902188650  Biso  1.000000 S
   Mo8         1.0  -0.11418266430330 -0.47293162852240 -0.43432406078490  Biso  1.000000 Mo
   Mo9         1.0  -0.23806074433410 -0.22215083008600 -0.43220371181300  Biso  1.000000 Mo
   Mo10        1.0  -0.40733533623600 0.11368172676520 -0.43344179021480  Biso  1.000000 Mo
   Mo11        1.0  0.49659123491400 0.37948804150540 -0.43523602921440  Biso  1.000000 Mo
   Mo12        1.0  0.27440907262340 -0.33174664473330 -0.43139868822770  Biso  1.000000 Mo
   Mo13        1.0  0.21160355121550 -0.03242137606565 -0.43469344648500  Biso  1.000000 Mo
   Mo14        1.0  0.00954452207710 0.27701277203830 -0.43307673337270  Biso  1.000000 Mo
   S15         1.0  0.12335403259410 -0.27629035508040 -0.47463631047950  Biso  1.000000 S
   S16         1.0  -0.01620291808228 0.01737451377843 -0.46752031453350  Biso  1.000000 S
   S17         1.0  -0.16437432622600 0.29322768957480 -0.47570462319150  Biso  1.000000 S
   S18         1.0  -0.29777709094840 -0.42408249991860 -0.47663797179090  Biso  1.000000 S
   S19         1.0  -0.44137917633410 -0.14392611467760 -0.46614669747270  Biso  1.000000 S
   S20         1.0  0.41492277657470 0.14616521440440 -0.47690034400350  Biso  1.000000 S
   S21         1.0  0.27453389093290 0.44011283299030 -0.46806467291060  Biso  1.000000 S
   S22         1.0  0.06283195868294 0.48591848508630 -0.39010547035880  Biso  1.000000 S
   S23         1.0  -0.04624549529761 -0.22782852465390 -0.39288343161800  Biso  1.000000 S
   S24         1.0  -0.20630090683610 0.03982730862262 -0.39755036869590  Biso  1.000000 S
   S25         1.0  -0.33919716035620 0.33969546413030 -0.39207069207200  Biso  1.000000 S
   S26         1.0  0.49776544606920 -0.38734887723300 -0.39836281785940  Biso  1.000000 S
   S27         1.0  0.37774397101580 -0.08078676529903 -0.39215918435530  Biso  1.000000 S
   S28         1.0  0.21997827679150 0.20325926786050 -0.39934424317690  Biso  1.000000 S
```

7. 1T-MoS_2/1T-MoS_2-120

```
_cell_length_a                          5.353347
_cell_length_b                          3.090008
_cell_length_c                          40.000000
_cell_angle_alpha                       90.000000
_cell_angle_beta                        90.000000
_cell_angle_gamma                       90.000000
_cell_volume                            661.675487
_space_group_name_H-M_alt               'C 2/m'
_space_group_IT_number                  12

loop_
_space_group_symop_operation_xyz
   'x, y, z'
   '-x, -y, -z'
   '-x, y, -z'
   'x, -y, z'
   'x+1/2, y+1/2, z'
   '-x+1/2, -y+1/2, -z'
   '-x+1/2, y+1/2, -z'
   'x+1/2, -y+1/2, z'

loop_
   _atom_site_label
   _atom_site_occupancy
   _atom_site_fract_x
   _atom_site_fract_y
   _atom_site_fract_z
   _atom_site_adp_type
   _atom_site_B_iso_or_equiv
   _atom_site_type_symbol
   Mo1        1.0    0.0005910992082194 0.0000000000000000 -0.4320565084204000    Biso  1.000000 Mo
   S1         1.0    0.1673009290802000 0.5000000000000000 -0.3924525000906000    Biso  1.000000 S
   S2         1.0    0.3338619639763000 0.0000000000000000 -0.4712506470757000    Biso  1.000000 S
```

8. $1H\text{-}MoS_2/1H\text{-}MoS_2$-0

```
_cell_length_a                        3.082780
_cell_length_b                        3.083200
_cell_length_c                        40.000000
_cell_angle_alpha                     90.000000
_cell_angle_beta                      90.000000
_cell_angle_gamma                     119.995003
_cell_volume                          329.273440
_space_group_name_H-M_alt             'P 1'
_space_group_IT_number                1

loop_
_space_group_symop_operation_xyz
   'x, y, z'

loop_
   _atom_site_label
   _atom_site_occupancy
   _atom_site_fract_x
   _atom_site_fract_y
   _atom_site_fract_z
   _atom_site_adp_type
   _atom_site_B_iso_or_equiv
   _atom_site_type_symbol
   MO001       1.0    -0.333329     0.333323     0.279592    Biso  1.000000 Mo
   S002        1.0     0.333333    -0.333328     0.240764    Biso  1.000000 S
   S003        1.0     0.333331    -0.333330     0.318239    Biso  1.000000 S
   MO004       1.0    -0.333326     0.333326     0.436801    Biso  1.000000 Mo
   S005        1.0     0.333334    -0.333327     0.398154    Biso  1.000000 S
   S006        1.0     0.333337    -0.333325     0.475629    Biso  1.000000 S
```

9. 1H-MoS_2/1H-MoS_2-27.8

```
_cell_length_a                        8.156399
_cell_length_b                        8.156982
_cell_length_c                        40.000000
_cell_angle_alpha                     90.000000
_cell_angle_beta                      90.000000
_cell_angle_gamma                     60.007999
_cell_volume                          2304.908035
_space_group_name_H-M_alt             'P 1'
_space_group_IT_number                1

loop_
_space_group_symop_operation_xyz
   'x, y, z'

loop_
   _atom_site_label
   _atom_site_occupancy
   _atom_site_fract_x
   _atom_site_fract_y
   _atom_site_fract_z
   _atom_site_adp_type
   _atom_site_B_iso_or_equiv
   _atom_site_type_symbol
   MO001       1.0    -0.476148     0.094559     0.315581    Biso  1.000000 Mo
   MO002       1.0    -0.188308     0.238163     0.315896    Biso  1.000000 Mo
   MO003       1.0     0.096966     0.380846     0.315566    Biso  1.000000 Mo
   MO004       1.0     0.383323    -0.478665     0.315591    Biso  1.000000 Mo
   MO005       1.0    -0.332037    -0.334368     0.315930    Biso  1.000000 Mo
   MO006       1.0    -0.048097    -0.190839     0.315880    Biso  1.000000 Mo
   MO007       1.0     0.240687    -0.050656     0.315909    Biso  1.000000 Mo
   S008        1.0     0.477522    -0.095932     0.354719    Biso  1.000000 S
   S009        1.0    -0.238065     0.047734     0.354632    Biso  1.000000 S
   S010        1.0     0.050272     0.189582     0.354637    Biso  1.000000 S
   S011        1.0     0.334669     0.332219     0.354480    Biso  1.000000 S
   S012        1.0    -0.379832     0.474998     0.354714    Biso  1.000000 S
   S013        1.0    -0.093488    -0.382315     0.354693    Biso  1.000000 S
   S014        1.0     0.191988    -0.240627     0.354644    Biso  1.000000 S
   S015        1.0     0.478312    -0.098969     0.276856    Biso  1.000000 S
   S016        1.0    -0.238396     0.050701     0.277564    Biso  1.000000 S
   S017        1.0     0.053275     0.186785     0.277564    Biso  1.000000 S
   S018        1.0     0.334690     0.332290     0.276507    Biso  1.000000 S
   S019        1.0    -0.377627     0.475901     0.276853    Biso  1.000000 S
   S020        1.0    -0.096903    -0.380003     0.276848    Biso  1.000000 S
   S021        1.0     0.189660    -0.241040     0.277561    Biso  1.000000 S
   MO022       1.0     0.476046    -0.380740     0.168012    Biso  1.000000 Mo
   MO023       1.0     0.048014    -0.238112     0.167692    Biso  1.000000 Mo
   MO024       1.0     0.188380     0.050657     0.167722    Biso  1.000000 Mo
   MO025       1.0    -0.383371    -0.094474     0.168000    Biso  1.000000 Mo
   MO026       1.0    -0.240558     0.190916     0.167734    Biso  1.000000 Mo
   MO027       1.0     0.332131     0.334392     0.167699    Biso  1.000000 Mo
   MO028       1.0    -0.096974     0.478699     0.168033    Biso  1.000000 Mo
   S029        1.0    -0.478194     0.380299     0.206782    Biso  1.000000 S
   S030        1.0     0.096879    -0.475985     0.206753    Biso  1.000000 S
   S031        1.0     0.238114    -0.186972     0.206023    Biso  1.000000 S
   S032        1.0    -0.334935    -0.332126     0.207067    Biso  1.000000 S
   S033        1.0    -0.189653    -0.050638     0.206019    Biso  1.000000 S
   S034        1.0     0.377801     0.098998     0.206778    Biso  1.000000 S
   S035        1.0    -0.052893     0.241042     0.206073    Biso  1.000000 S
   S036        1.0    -0.477386     0.382305     0.128909    Biso  1.000000 S
   S037        1.0     0.093613    -0.475107     0.128906    Biso  1.000000 S
   S038        1.0     0.238039    -0.189443     0.128957    Biso  1.000000 S
   S039        1.0    -0.334697    -0.332258     0.129112    Biso  1.000000 S
   S040        1.0    -0.192079    -0.047598     0.128957    Biso  1.000000 S
   S041        1.0     0.379802     0.096083     0.128898    Biso  1.000000 S
   S042        1.0    -0.050203     0.240657     0.128987    Biso  1.000000 S
```

10. 1H-MoS_2/1H-MoS_2-38.2

```
_cell_length_a                        8.156481
_cell_length_b                        8.156987
_cell_length_c                        40.000000
_cell_angle_alpha                     90.000000
_cell_angle_beta                      90.000000
_cell_angle_gamma                     119.993202
_cell_volume                          2304.904658
_space_group_name_H-M_alt             'P 1'
_space_group_IT_number                1

loop_
_space_group_symop_operation_xyz
   'x, y, z'

loop_
   _atom_site_label
   _atom_site_occupancy
   _atom_site_fract_x
   _atom_site_fract_y
   _atom_site_fract_z
   _atom_site_adp_type
   _atom_site_B_iso_or_equiv
   _atom_site_type_symbol
   MO001      1.0    -0.046505     0.190568     0.284153    Biso  1.000000 Mo
   MO002      1.0     0.093361    -0.382336     0.284118    Biso  1.000000 Mo
   MO003      1.0     0.236818     0.045954     0.284150    Biso  1.000000 Mo
   MO004      1.0     0.381796     0.475369     0.284099    Biso  1.000000 Mo
   MO005      1.0    -0.475862    -0.093813     0.284086    Biso  1.000000 Mo
   MO006      1.0    -0.333575     0.333032     0.284735    Biso  1.000000 Mo
   MO007      1.0    -0.191134    -0.237409     0.284144    Biso  1.000000 Mo
   S008       1.0     0.190928     0.237593     0.245222    Biso  1.000000 S
   S009       1.0     0.333051    -0.333610     0.245273    Biso  1.000000 S
   S010       1.0     0.476568     0.096445     0.245457    Biso  1.000000 S
   S011       1.0    -0.380433    -0.477150     0.245462    Biso  1.000000 S
   S012       1.0    -0.238083    -0.046925     0.245213    Biso  1.000000 S
   S013       1.0    -0.096889     0.379914     0.245475    Biso  1.000000 S
   S014       1.0     0.046417    -0.191533     0.245229    Biso  1.000000 S
   S015       1.0     0.192471     0.237105     0.322942    Biso  1.000000 S
   S016       1.0     0.333293    -0.333411     0.321786    Biso  1.000000 S
   S017       1.0     0.472356     0.093678     0.323145    Biso  1.000000 S
   S018       1.0    -0.378971    -0.473019     0.323135    Biso  1.000000 S
   S019       1.0    -0.237682    -0.044942     0.322934    Biso  1.000000 S
   S020       1.0    -0.094244     0.378351     0.323167    Biso  1.000000 S
   S021       1.0     0.044284    -0.193037     0.322948    Biso  1.000000 S
   MO022      1.0     0.382378    -0.093218     0.432291    Biso  1.000000 Mo
   MO023      1.0    -0.475326    -0.381743     0.432301    Biso  1.000000 Mo
   MO024      1.0    -0.190546     0.046558     0.432258    Biso  1.000000 Mo
   MO025      1.0    -0.333035     0.333659     0.431639    Biso  1.000000 Mo
   MO026      1.0    -0.045915    -0.236754     0.432241    Biso  1.000000 Mo
   MO027      1.0     0.093873     0.475990     0.432297    Biso  1.000000 Mo
   MO028      1.0     0.237400     0.191166     0.432243    Biso  1.000000 Mo
   S029       1.0    -0.378306     0.094228     0.393237    Biso  1.000000 S
   S030       1.0    -0.237078    -0.192404     0.393464    Biso  1.000000 S
   S031       1.0     0.044873     0.237687     0.393472    Biso  1.000000 S
   S032       1.0    -0.093671    -0.472283     0.393228    Biso  1.000000 S
   S033       1.0     0.193042    -0.044302     0.393432    Biso  1.000000 S
   S034       1.0     0.333637    -0.333005     0.394629    Biso  1.000000 S
   S035       1.0     0.472858     0.378992     0.393233    Biso  1.000000 S
   S036       1.0    -0.379863     0.097059     0.470925    Biso  1.000000 S
   S037       1.0    -0.237557    -0.190904     0.471181    Biso  1.000000 S
   S038       1.0     0.046997     0.238223     0.471179    Biso  1.000000 S
   S039       1.0    -0.096449    -0.476536     0.470920    Biso  1.000000 S
   S040       1.0     0.191501    -0.046345     0.471170    Biso  1.000000 S
   S041       1.0     0.333632    -0.332981     0.471125    Biso  1.000000 S
   S042       1.0     0.477151     0.380439     0.470923    Biso  1.000000 S
```

11. 1H-MoS_2/1H-MoS_2-60

```
_cell_length_a                       16.019300
_cell_length_b                       50.751499
_cell_length_c                       40.000000
_cell_angle_alpha                    90.000000
_cell_angle_beta                     90.000000
_cell_angle_gamma                    1.740700
_cell_volume                         987.840668
_space_group_name_H-M_alt            'P 1'
_space_group_IT_number               1

loop_
_space_group_symop_operation_xyz
   'x, y, z'

loop_
   _atom_site_label
   _atom_site_occupancy
   _atom_site_fract_x
   _atom_site_fract_y
   _atom_site_fract_z
   _atom_site_adp_type
   _atom_site_B_iso_or_equiv
   _atom_site_type_symbol
   MO001       1.0    -0.423335    -0.041027     0.283205    Biso  1.000000 Mo
   MO002       1.0    -0.090048    -0.041012     0.283205    Biso  1.000000 Mo
   MO003       1.0     0.243276    -0.041009     0.283205    Biso  1.000000 Mo
   S004        1.0    -0.335068    -0.033936     0.246117    Biso  1.000000 S
   S005        1.0    -0.001770    -0.033924     0.246117    Biso  1.000000 S
   S006        1.0     0.331540    -0.033917     0.246116    Biso  1.000000 S
   S007        1.0    -0.344799    -0.030916     0.320068    Biso  1.000000 S
   S008        1.0    -0.011501    -0.030905     0.320067    Biso  1.000000 S
   S009        1.0     0.321786    -0.030889     0.320066    Biso  1.000000 S
   MO010       1.0     0.089644     0.041143     0.433190    Biso  1.000000 Mo
   MO011       1.0    -0.243680     0.041140     0.433187    Biso  1.000000 Mo
   MO012       1.0     0.422978     0.041143     0.433188    Biso  1.000000 Mo
   S013        1.0     0.011212     0.031000     0.396327    Biso  1.000000 S
   S014        1.0    -0.322215     0.031029     0.396325    Biso  1.000000 S
   S015        1.0     0.344357     0.031058     0.396326    Biso  1.000000 S
   S016        1.0     0.001278     0.034083     0.470277    Biso  1.000000 S
   S017        1.0    -0.331923     0.034042     0.470276    Biso  1.000000 S
   S018        1.0     0.334758     0.034037     0.470277    Biso  1.000000 S
```

12. 1H-MoS_2/1H-MoS_2-98.2

```
_cell_length_a                       8.156415
_cell_length_b                       8.156900
_cell_length_c                       40.000000
_cell_angle_alpha                    90.000000
_cell_angle_beta                     90.000000
_cell_angle_gamma                    60.008202
_cell_volume                         2304.894135
_space_group_name_H-M_alt            'P 1'
_space_group_IT_number               1

loop_
_space_group_symop_operation_xyz
   'x, y, z'

loop_
   _atom_site_label
   _atom_site_occupancy
   _atom_site_fract_x
   _atom_site_fract_y
   _atom_site_fract_z
   _atom_site_adp_type
   _atom_site_B_iso_or_equiv
   _atom_site_type_symbol
   MO001      1.0   -0.186730    0.237970    0.315904   Biso  1.000000 Mo
   MO002      1.0   -0.330609   -0.334536    0.315925   Biso  1.000000 Mo
   MO003      1.0    0.384871   -0.478918    0.315605   Biso  1.000000 Mo
   MO004      1.0   -0.474620    0.094396    0.315567   Biso  1.000000 Mo
   MO005      1.0    0.242170   -0.050747    0.315894   Biso  1.000000 Mo
   MO006      1.0   -0.046558   -0.191051    0.315890   Biso  1.000000 Mo
   MO007      1.0    0.098429    0.380678    0.315561   Biso  1.000000 Mo
   S008       1.0    0.051834    0.189391    0.354637   Biso  1.000000 S
   S009       1.0   -0.092030   -0.382505    0.354693   Biso  1.000000 S
   S010       1.0   -0.378297    0.474811    0.354722   Biso  1.000000 S
   S011       1.0   -0.236576    0.047544    0.354633   Biso  1.000000 S
   S012       1.0    0.478999   -0.096058    0.354704   Biso  1.000000 S
   S013       1.0    0.193529   -0.240757    0.354646   Biso  1.000000 S
   S014       1.0    0.336175    0.331983    0.354479   Biso  1.000000 S
   S015       1.0    0.054689    0.186737    0.277526   Biso  1.000000 S
   S016       1.0   -0.095528   -0.380101    0.276860   Biso  1.000000 S
   S017       1.0   -0.376114    0.475569    0.276854   Biso  1.000000 S
   S018       1.0   -0.236688    0.050477    0.277578   Biso  1.000000 S
   S019       1.0    0.479810   -0.099099    0.276841   Biso  1.000000 S
   S020       1.0    0.191304   -0.241335    0.277593   Biso  1.000000 S
   S021       1.0    0.336106    0.332171    0.276504   Biso  1.000000 S
   MO022      1.0   -0.242065    0.190982    0.167748   Biso  1.000000 Mo
   MO023      1.0   -0.384913   -0.094439    0.167996   Biso  1.000000 Mo
   MO024      1.0    0.474550   -0.380657    0.168020   Biso  1.000000 Mo
   MO025      1.0    0.330643    0.334425    0.167693   Biso  1.000000 Mo
   MO026      1.0    0.186794    0.050784    0.167707   Biso  1.000000 Mo
   MO027      1.0    0.046482   -0.238046    0.167692   Biso  1.000000 Mo
   MO028      1.0   -0.098476    0.478679    0.168043   Biso  1.000000 Mo
   S029       1.0   -0.191268   -0.050597    0.206018   Biso  1.000000 S
   S030       1.0   -0.336328   -0.332098    0.207079   Biso  1.000000 S
   S031       1.0   -0.479748    0.380379    0.206784   Biso  1.000000 S
   S032       1.0    0.376215    0.099047    0.206769   Biso  1.000000 S
   S033       1.0    0.236543   -0.186758    0.205996   Biso  1.000000 S
   S034       1.0    0.095417   -0.475990    0.206734   Biso  1.000000 S
   S035       1.0   -0.054363    0.241003    0.206111   Biso  1.000000 S
   S036       1.0   -0.193602   -0.047517    0.128964   Biso  1.000000 S
   S037       1.0   -0.336189   -0.332250    0.129117   Biso  1.000000 S
   S038       1.0   -0.478874    0.382375    0.128917   Biso  1.000000 S
   S039       1.0    0.378289    0.096140    0.128887   Biso  1.000000 S
   S040       1.0    0.236536   -0.189417    0.128953   Biso  1.000000 S
   S041       1.0    0.092064   -0.475056    0.128909   Biso  1.000000 S
   S042       1.0   -0.051802    0.240750    0.128985   Biso  1.000000 S
```

13. 1H-MoS_2/1H-MoS_2-120

```
_chemical_name_common              '1H-MoS2/1H-MoS2-120'
_cell_length_a                     50.751499
_cell_length_b                     45.414501
_cell_length_c                     40.000000
_cell_angle_alpha                  90.000000
_cell_angle_beta                   90.000000
_cell_angle_gamma                  0.204633
_cell_volume                       658.545098
_space_group_name_H-M_alt          'P 1'
_space_group_IT_number             1

loop_
_space_group_symop_operation_xyz
   'x, y, z'

loop_
   _atom_site_label
   _atom_site_occupancy
   _atom_site_fract_x
   _atom_site_fract_y
   _atom_site_fract_z
   _atom_site_adp_type
   _atom_site_B_iso_or_equiv
   _atom_site_type_symbol
   Mo1        1.0     0.333620     0.666350     0.28944    Biso  1.000000 Mo
   S1         1.0     0.666520     0.333490     0.25068    Biso  1.000000 S
   S2         1.0     0.666520     0.333490     0.32818    Biso  1.000000 S
   Mo2        1.0     0.331790     0.668390     0.42696    Biso  1.000000 Mo
   S3         1.0     0.665860     0.334230     0.3882    Biso  1.000000 S
   S4         1.0     0.665860     0.334230     0.46572    Biso  1.000000 S
```

14. $MoSe_2/MoSe_2$-0

```
_cell_length_a                         3.19374621
_cell_length_b                         3.19374621
_cell_length_c                         40.000000
_cell_angle_alpha                      90.000000
_cell_angle_beta                       90.000000
_cell_angle_gamma                      120.000000
_symmetry_space_group_name_H-M         'P 1'
_symmetry_Int_Tables_number            1

loop_
_symmetry_equiv_pos_as_xyz
   'x, y, z'

loop_
   _atom_site_label
   _atom_site_type_symbol
   _atom_site_fract_x
   _atom_site_fract_y
   _atom_site_fract_z
MO001  MO   0.333333333333  -0.333333333333   0.4175461185131
MO002  MO   0.333333333333  -0.333333333333  -0.4175461185131
SE003  SE   0.000000000000   0.000000000000   0.3764105233261
SE004  SE   0.000000000000   0.000000000000  -0.3764105233261
SE005  SE   0.000000000000   0.000000000000   0.4585535576066
SE006  SE   0.000000000000   0.000000000000  -0.4585535576066
```

15. $MoSe_2/MoSe_2$-27.8

```
_cell_length_a                         11.51557103
_cell_length_b                         19.94588378
_cell_length_c                         40.000000
_cell_angle_alpha                      90.000000
_cell_angle_beta                       90.000000
_cell_angle_gamma                      90.000000
_cell_volume                           9187.074769
_space_group_name_H-M_alt              'C 2'
_space_group_IT_number                 5

loop_
_space_group_symop_operation_xyz
   'x, y, z'
   '-x, y, -z'
   'x+1/2, y+1/2, z'
   '-x+1/2, y+1/2, -z'

loop_
   _atom_site_label
   _atom_site_occupancy
   _atom_site_fract_x
   _atom_site_fract_y
   _atom_site_fract_z
   _atom_site_adp_type
   _atom_site_B_iso_or_equiv
   _atom_site_type_symbol
   Mo1         1.0    -0.11492917080920 0.06518646814571 -0.42217581867030    Biso  1.000000 Mo
   Mo2         1.0    -0.38325824687060 0.10170112417040 -0.42216915313460    Biso  1.000000 Mo
   Mo3         1.0    0.34706211352720 0.14194126570930 -0.42214798219720    Biso  1.000000 Mo
   Mo4         1.0    0.07858490722863 0.17819069580230 -0.42200638721730    Biso  1.000000 Mo
   Mo5         1.0    -0.18916697094070 0.21807592233700 -0.42231448219930    Biso  1.000000 Mo
   Mo6         1.0    0.04226945022502 -0.24358959997380 -0.42214385500130    Biso  1.000000 Mo
   Mo7         1.0    -0.22935151047260 -0.20466752437290 -0.42234534710390    Biso  1.000000 Mo
   Mo8         1.0    -0.49784401316920 -0.16661647375390 -0.42235199790160    Biso  1.000000 Mo
   Mo9         1.0    0.23097415581290 -0.12745946596540 -0.42201164858250    Biso  1.000000 Mo
   Mo10        1.0    -0.03735373050350 -0.09110845852107 -0.42216698811880    Biso  1.000000 Mo
   Mo11        1.0    -0.30390889414100 -0.05079603136967 -0.42200948939250    Biso  1.000000 Mo
   Mo12        1.0    0.42475771890410 -0.01358330459993 -0.42231654032270    Biso  1.000000 Mo
   Mo13        1.0    0.15860777634060 0.02591895287130 -0.42214070981890    Biso  1.000000 Mo
   Se1         1.0    -0.07528018340472 0.15401742096890 -0.38080477647160    Biso  1.000000 Se
   Se2         1.0    -0.34320519259180 0.19177102851680 -0.38114397055580    Biso  1.000000 Se
   Se3         1.0    -0.11298839069170 -0.26852523752440 -0.38114579993130    Biso  1.000000 Se
   Se4         1.0    0.11699878027940 0.26888778033960 -0.38120174593890    Biso  1.000000 Se
   Se5         1.0    0.34799711947770 -0.19187950799220 -0.38119215067440    Biso  1.000000 Se
   Se6         1.0    0.07849370914347 -0.15406998313320 -0.38075720881050    Biso  1.000000 Se
   Se7         1.0    -0.19051250287470 -0.11564837413300 -0.38082894435690    Biso  1.000000 Se
   Se8         1.0    -0.45879556164890 -0.07704077562841 -0.38114403825740    Biso  1.000000 Se
   Se9         1.0    0.27178712036350 -0.03846136852104 -0.38078807718790    Biso  1.000000 Se
   Se10        1.0    0.00211726075797 -0.00000486832128 -0.38145057379480    Biso  1.000000 Se
   Se11        1.0    -0.26731916616120 0.03875027351122 -0.38077016872350    Biso  1.000000 Se
   Se12        1.0    0.46234678008480 0.07673892745799 -0.38119540989430    Biso  1.000000 Se
   Se13        1.0    0.19481765482650 0.11533318741920 -0.38072906666930    Biso  1.000000 Se
   Se14        1.0    -0.07432918235996 0.15419564059440 -0.46362907500170    Biso  1.000000 Se
   Se15        1.0    -0.34266133614540 0.19088303431900 -0.46292468420700    Biso  1.000000 Se
   Se16        1.0    -0.11163207168090 -0.26786499249480 -0.46293140214020    Biso  1.000000 Se
   Se17        1.0    0.11599619258320 0.26734272547730 -0.46290488596980    Biso  1.000000 Se
   Se18        1.0    0.34628742309560 -0.19061368074970 -0.46289473944620    Biso  1.000000 Se
   Se19        1.0    0.07863751532568 -0.15432609650690 -0.46354766551840    Biso  1.000000 Se
   Se20        1.0    -0.19137297057860 -0.11511014495330 -0.46360578060090    Biso  1.000000 Se
   Se21        1.0    -0.45610117319050 -0.07678355893709 -0.46301557725590    Biso  1.000000 Se
   Se22        1.0    0.27147390304240 -0.03906688878694 -0.46362734743640    Biso  1.000000 Se
   Se23        1.0    0.00239452395123 0.00000326308677 -0.46178664538940    Biso  1.000000 Se
   Se24        1.0    -0.26774427638950 0.03867236474224 -0.46355911941500    Biso  1.000000 Se
   Se25        1.0    0.46056472684350 0.07696609634687 -0.46280451176760    Biso  1.000000 Se
   Se26        1.0    0.19490046176140 0.11557016442260 -0.46357954253020    Biso  1.000000 Se
```

16. $MoSe_2/MoSe_2$-32.2

```
_cell_length_a                          19.94618298
_cell_length_b                          11.51501518
_cell_length_c                          40.000000
_cell_angle_alpha                       90.000000
_cell_angle_beta                        90.000000
_cell_angle_gamma                       90.000000
_cell_volume                            9187.223993
_space_group_name_H-M_alt               'C 2'
_space_group_IT_number                  5

loop_
_space_group_symop_operation_xyz
   'x, y, z'
   '-x, y, -z'
   'x+1/2, y+1/2, z'
   '-x+1/2, y+1/2, -z'

loop_
   _atom_site_label
   _atom_site_occupancy
   _atom_site_fract_x
   _atom_site_fract_y
   _atom_site_fract_z
   _atom_site_adp_type
   _atom_site_B_iso_or_equiv
   _atom_site_type_symbol
   Mo1        1.0   -0.0248163914420 0.1563853354369 0.4221526240923    Biso  1.000000 Mo
   Mo2        1.0   -0.1406470289124 0.3452260038727 0.4221353287415    Biso  1.000000 Mo
   Mo3        1.0   0.2446950370937 0.0403940727636 0.4221051661703    Biso  1.000000 Mo
   Mo4        1.0   0.1284806838866 0.2289319079752 0.4219793973635    Biso  1.000000 Mo
   Mo5        1.0   0.0146892067657 0.4227448506588 0.4223232087392    Biso  1.000000 Mo
   Mo6        1.0   -0.1006102076328 -0.3854406321470 0.4221226994889    Biso  1.000000 Mo
   Mo7        1.0   -0.2168559181149 -0.1912763574610 0.4223496214482    Biso  1.000000 Mo
   Mo8        1.0   0.1678865864034 -0.4998842249687 0.4223005897692    Biso  1.000000 Mo
   Mo9        1.0   0.0518899318927 -0.3056722230521 0.4219923720824    Biso  1.000000 Mo
   Mo10       1.0   -0.0640293027206 -0.1169928448982 0.4221537645672    Biso  1.000000 Mo
   Mo11       1.0   -0.1771593855532 0.0767117785972 0.4219809402615    Biso  1.000000 Mo
   Mo12       1.0   0.2057350634530 -0.2316819318999 0.4223433134261    Biso  1.000000 Mo
   Mo13       1.0   0.0924418388527 -0.0393051631481 0.4221214839770    Biso  1.000000 Mo
   Se1        1.0   -0.1141866388809 0.1928309075306 0.3807203679066    Biso  1.000000 Se
   Se2        1.0   0.2696744138199 -0.1150895536079 0.3811743781757    Biso  1.000000 Se
   Se3        1.0   0.1552393603791 0.0766198863002 0.3806820511472    Biso  1.000000 Se
   Se4        1.0   0.0394814336349 0.2697186081884 0.3807940961415    Biso  1.000000 Se
   Se5        1.0   -0.0755309655054 0.4603263144438 0.3811467342723    Biso  1.000000 Se
   Se6        1.0   -0.1906393985072 -0.3451907785752 0.3811150353895    Biso  1.000000 Se
   Se7        1.0   0.1930772503655 0.3458511137448 0.3811880099824    Biso  1.000000 Se
   Se8        1.0   0.0781882933452 -0.4607540363527 0.3811530572472    Biso  1.000000 Se
   Se9        1.0   -0.0376519444328 -0.2693541998947 0.3807306021970    Biso  1.000000 Se
   Se10       1.0   -0.1528901834104 -0.0772618025427 0.3808217632341    Biso  1.000000 Se
   Se11       1.0   -0.2677534553144 0.1149425171347 0.3811303437605    Biso  1.000000 Se
   Se12       1.0   0.1168216357998 -0.1925337137778 0.3807998404905    Biso  1.000000 Se
   Se13       1.0   0.0012046771373 0.0000261033282 0.3814406856398    Biso  1.000000 Se
   Se14       1.0   -0.1144656370639 0.1928836608284 0.4635378582977    Biso  1.000000 Se
   Se15       1.0   0.2690113600895 -0.1135987418326 0.4628158634887    Biso  1.000000 Se
   Se16       1.0   0.1554455102041 0.0768785340205 0.4635399465440    Biso  1.000000 Se
   Se17       1.0   0.0400657974785 0.2694141732193 0.4636414587088    Biso  1.000000 Se
   Se18       1.0   -0.0758085765615 0.4587245639730 0.4628819831142    Biso  1.000000 Se
   Se19       1.0   -0.1896583821304 -0.3450549685230 0.4629562425538    Biso  1.000000 Se
   Se20       1.0   0.1917208549681 0.3441412292336 0.4628391260606    Biso  1.000000 Se
   Se21       1.0   0.0780706177984 -0.4579779696088 0.4629041148795    Biso  1.000000 Se
   Se22       1.0   -0.0377104473368 -0.2698009264767 0.4635138049168    Biso  1.000000 Se
   Se23       1.0   -0.1530187278193 -0.0760047909230 0.4636102449445    Biso  1.000000 Se
   Se24       1.0   -0.2662622407245 0.1137926489443 0.4629770833714    Biso  1.000000 Se
   Se25       1.0   0.1162587212313 -0.1932775193529 0.4636278689255    Biso  1.000000 Se
   Se26       1.0   0.0013641328400 0.0000081688489 0.4617649503577    Biso  1.000000 Se
```

17. $MoSe_2/MoSe_2$-60

```
_cell_length_a                        3.19377667
_cell_length_b                        3.19377667
_cell_length_c                        40.000000
_cell_angle_alpha                     90.000000
_cell_angle_beta                      90.000000
_cell_angle_gamma                     120.000000
_symmetry_space_group_name_H-M        'P 1'
_symmetry_Int_Tables_number           1

loop_
_symmetry_equiv_pos_as_xyz
   'x, y, z'

loop_
   _atom_site_label
   _atom_site_type_symbol
   _atom_site_fract_x
   _atom_site_fract_y
   _atom_site_fract_z
MO001  MO   0.333333333333  -0.333333333333  -0.4177853392350
MO002  MO  -0.333333333333   0.333333333333   0.4177853392350
SE003  SE   0.000000000000   0.000000000000  -0.3766553517135
SE004  SE   0.000000000000   0.000000000000   0.3766553517135
SE005  SE   0.000000000000   0.000000000000  -0.4587930117346
SE006  SE   0.000000000000   0.000000000000   0.4587930117346
```

18. $MoSe_2/MoSe_2$-92.2

```
_cell_length_a                      11.51494005
_cell_length_b                      19.94614746
_cell_length_c                      40.000000
_cell_angle_alpha                   90.000000
_cell_angle_beta                    90.000000
_cell_angle_gamma                   90.000000
_cell_volume                        9187.148070
_space_group_name_H-M_alt           'C 2'
_space_group_IT_number              5

loop_
_space_group_symop_operation_xyz
   'x, y, z'
   '-x, y, -z'
   'x+1/2, y+1/2, z'
   '-x+1/2, y+1/2, -z'

loop_
   _atom_site_label
   _atom_site_occupancy
   _atom_site_fract_x
   _atom_site_fract_y
   _atom_site_fract_z
   _atom_site_adp_type
   _atom_site_B_iso_or_equiv
   _atom_site_type_symbol
   Mo1         1.0    0.4978663775126  0.1665837371404 -0.4223524871244    Biso  1.000000 Mo
   Mo2         1.0    0.3039274494144  0.0507611159103 -0.4220097980276    Biso  1.000000 Mo
   Mo3         1.0    0.1149532999578 -0.0652192151589 -0.4221759099103    Biso  1.000000 Mo
   Mo4         1.0   -0.1585856732816 -0.0259535459983 -0.4221412152418    Biso  1.000000 Mo
   Mo5         1.0   -0.0785649063005 -0.1782209563115 -0.4220067203063    Biso  1.000000 Mo
   Mo6         1.0   -0.3470433136545 -0.1419730239132 -0.4221483085724    Biso  1.000000 Mo
   Mo7         1.0    0.2293750080524  0.2046365271406 -0.4223456226327    Biso  1.000000 Mo
   Mo8         1.0   -0.0422481732103  0.2435555547912 -0.4221443254578    Biso  1.000000 Mo
   Mo9         1.0    0.0373729803879  0.0910772139047 -0.4221673010623    Biso  1.000000 Mo
   Mo10        1.0   -0.2309522454265  0.1274281163318 -0.4220119588501    Biso  1.000000 Mo
   Mo11        1.0   -0.4247354635981  0.0135507751503 -0.4223169580008    Biso  1.000000 Mo
   Mo12        1.0    0.3832811267573 -0.1017291265360 -0.4221692807391    Biso  1.000000 Mo
   Mo13        1.0    0.1891860133620 -0.2181064227409 -0.4223150147098    Biso  1.000000 Mo
   Se1         1.0    0.4588164728258  0.0770081715652 -0.3811448066460    Biso  1.000000 Se
   Se2         1.0    0.2673405539379 -0.0387823514816 -0.3807695912937    Biso  1.000000 Se
   Se3         1.0    0.0753010100080 -0.1540487567819 -0.3808049663087    Biso  1.000000 Se
   Se4         1.0   -0.1947982479342 -0.1153654984204 -0.3807292191825    Biso  1.000000 Se
   Se5         1.0   -0.1169749576144 -0.2689191405683 -0.3812024291094    Biso  1.000000 Se
   Se6         1.0    0.1130094493545  0.2684929657682 -0.3811463373371    Biso  1.000000 Se
   Se7         1.0    0.1905340964797  0.1156168468575 -0.3808297708812    Biso  1.000000 Se
   Se8         1.0   -0.0784728778114  0.1540380203214 -0.3807571678163    Biso  1.000000 Se
   Se9         1.0   -0.0020950811735 -0.0000273565167 -0.3814512942271    Biso  1.000000 Se
   Se10        1.0   -0.2717636918177  0.0384290862964 -0.3807889141067    Biso  1.000000 Se
   Se11        1.0   -0.4623239082659 -0.0767696059654 -0.3811954345540    Biso  1.000000 Se
   Se12        1.0    0.3432276578293 -0.1918016568910 -0.3811453018907    Biso  1.000000 Se
   Se13        1.0   -0.3479752771011  0.1918483892743 -0.3811927999924    Biso  1.000000 Se
   Se14        1.0    0.4561211565898  0.0767507347350 -0.4630150272671    Biso  1.000000 Se
   Se15        1.0    0.2677656732925 -0.0387048480426 -0.4635600444765    Biso  1.000000 Se
   Se16        1.0    0.0743483105615 -0.1542266243387 -0.4636294212488    Biso  1.000000 Se
   Se17        1.0   -0.1948820523370 -0.1156023651951 -0.4635799300872    Biso  1.000000 Se
   Se18        1.0   -0.1159719336611 -0.2673742568452 -0.4629052800168    Biso  1.000000 Se
   Se19        1.0    0.1116545308525  0.2678323537973 -0.4629313204739    Biso  1.000000 Se
   Se20        1.0    0.1913937420654  0.1150792041868 -0.4636057511530    Biso  1.000000 Se
   Se21        1.0   -0.0786182243377  0.1542934411730 -0.4635485301909    Biso  1.000000 Se
   Se22        1.0   -0.0023691150871 -0.0000355106588 -0.4617864705703    Biso  1.000000 Se
   Se23        1.0   -0.2714504655905  0.0390348360532 -0.4636272639184    Biso  1.000000 Se
   Se24        1.0   -0.4605426818497 -0.0769970416706 -0.4628055913904    Biso  1.000000 Se
   Se25        1.0    0.3426849240318 -0.1909124841549 -0.4629239159392    Biso  1.000000 Se
   Se26        1.0   -0.3462645100713  0.1905826977921 -0.4628953677567    Biso  1.000000 Se
```

19. $MoSe_2/MoSe_2$-120

```
_cell_length_a                        3.193095
_cell_length_b                        3.193859
_cell_length_c                        40.000000
_cell_angle_alpha                     90.000000
_cell_angle_beta                      90.000000
_cell_angle_gamma                     119.981201
_cell_volume                          353.346219
_space_group_name_H-M_alt             'P 1'
_space_group_IT_number                1

loop_
_space_group_symop_operation_xyz
   'x, y, z'

loop_
   _atom_site_label
   _atom_site_occupancy
   _atom_site_fract_x
   _atom_site_fract_y
   _atom_site_fract_z
   _atom_site_adp_type
   _atom_site_B_iso_or_equiv
   _atom_site_type_symbol
   MO001       1.0     0.333393    -0.333383    -0.417571    Biso  1.000000 Mo
   MO002       1.0     0.333367    -0.333353     0.417571    Biso  1.000000 Mo
   SE003       1.0     0.000056     0.000001    -0.376430    Biso  1.000000 Se
   SE004       1.0     0.000026     0.000034     0.376428    Biso  1.000000 Se
   SE005       1.0     0.000047     0.000009    -0.458585    Biso  1.000000 Se
   SE006       1.0     0.000031     0.000032     0.458587    Biso  1.000000 Se
```

20. $MoTe_2$/$MoTe_2$-0

```
_cell_length_a                          3.417701
_cell_length_b                          3.417701
_cell_length_c                          40.000000
_cell_angle_alpha                       90.000000
_cell_angle_beta                        90.000000
_cell_angle_gamma                       120.000000
_cell_volume                            404.630574
_space_group_name_H-M_alt               'P -6 m 2'
_space_group_IT_number                  187

loop_
_space_group_symop_operation_xyz
   'x, y, z'
   '-y, x-y, z'
   '-x+y, -x, z'
   'x, y, -z'
   '-y, x-y, -z'
   '-x+y, -x, -z'
   '-y, -x, z'
   '-x+y, y, z'
   'x, x-y, z'
   '-y, -x, -z'
   '-x+y, y, -z'
   'x, x-y, -z'

loop_
   _atom_site_label
   _atom_site_occupancy
   _atom_site_fract_x
   _atom_site_fract_y
   _atom_site_fract_z
   _atom_site_adp_type
   _atom_site_B_iso_or_equiv
   _atom_site_type_symbol
   Te1         1.0   0.3333333333333 -0.3333333333333 -0.4530734656137   Biso  1.000000 Te
   Te2         1.0   0.3333333333333 -0.3333333333333 -0.3652626179084   Biso  1.000000 Te
   Mo1         1.0   0.0000000000000 0.0000000000000 -0.4092143134555   Biso  1.000000 Mo
```

21. $MoTe_2/MoTe_2$-17.9

```
_cell_length_a                          19.028999
_cell_length_b                          19.028999
_cell_length_c                          40.000000
_cell_angle_alpha                       90.000000
_cell_angle_beta                        90.000000
_cell_angle_gamma                       120.000000
_cell_volume                            12543.609478
_space_group_name_H-M_alt               'P 3 1 2'
_space_group_IT_number                  149

loop_
_space_group_symop_operation_xyz
   'x, y, z'
   '-y, x-y, z'
   '-x+y, -x, z'
   '-y, -x, -z'
   '-x+y, y, -z'
   'x, x-y, -z'

loop_
   _atom_site_label
   _atom_site_occupancy
   _atom_site_fract_x
   _atom_site_fract_y
   _atom_site_fract_z
   _atom_site_adp_type
   _atom_site_B_iso_or_equiv
   _atom_site_type_symbol
   Te1        1.0    0.11829144372420 0.07849345234184 -0.45806888216840    Biso  1.000000 Te
   Te2        1.0    0.15344010410530 -0.08298245139673 -0.45916581095190    Biso  1.000000 Te
   Te3        1.0    0.18053751324170 -0.24932872162750 -0.45923424284090    Biso  1.000000 Te
   Te4        1.0    0.21504485878830 -0.41231331305880 -0.45811275474300    Biso  1.000000 Te
   Te5        1.0    0.27894208278540 0.26932952839770 -0.46029160475770    Biso  1.000000 Te
   Te6        1.0    0.31287692484410 0.10807951962420 -0.45931706209570    Biso  1.000000 Te
   Te7        1.0    0.34264956935070 -0.05408528731241 -0.45946931124810    Biso  1.000000 Te
   Te8        1.0    0.44197297103010 0.46309378765360 -0.45974762110460    Biso  1.000000 Te
   Te9        1.0    0.47150207723420 0.29738155181710 -0.45914138423180    Biso  1.000000 Te
   Te10       1.0    -0.49336291440800 0.13741183061540 -0.45888148226400    Biso  1.000000 Te
   Te11       1.0    -0.33333333333330 0.33333333333330 -0.45722812442990    Biso  1.000000 Te
   Te12       1.0    0.11922646545500 0.07779819413822 -0.37152452804190    Biso  1.000000 Te
   Te13       1.0    0.15180702900200 -0.08558327309894 -0.37123769900050    Biso  1.000000 Te
   Te14       1.0    0.18030730568170 -0.24882585198220 -0.37150037358670    Biso  1.000000 Te
   Te15       1.0    0.21425651547840 -0.41055499286770 -0.37148694482490    Biso  1.000000 Te
   Te16       1.0    0.27999169302480 0.26983460919860 -0.37142923560390    Biso  1.000000 Te
   Te17       1.0    0.31219161537270 0.10826813349370 -0.37117769819050    Biso  1.000000 Te
   Te18       1.0    0.34363457197610 -0.05371242602672 -0.37075530529340    Biso  1.000000 Te
   Te19       1.0    0.44056913609280 0.46158278714010 -0.37154517067930    Biso  1.000000 Te
   Te20       1.0    0.47095526940250 0.29942275059120 -0.37148749829220    Biso  1.000000 Te
   Te21       1.0    -0.49420412769550 0.13819477742290 -0.37110976308780    Biso  1.000000 Te
   Te22       1.0    -0.33333333333330 0.33333333333330 -0.37166996022000    Biso  1.000000 Te
   Mo1        1.0    0.07826836817567 -0.04083810295376 -0.41495528340200    Biso  1.000000 Mo
   Mo2        1.0    0.10682435343540 -0.20371529322270 -0.41574618754400    Biso  1.000000 Mo
   Mo3        1.0    0.13879850493560 -0.36719885202260 -0.41554103719700    Biso  1.000000 Mo
   Mo4        1.0    0.17140910674040 0.46941246471130 -0.41500353087090    Biso  1.000000 Mo
   Mo5        1.0    0.23883721470770 0.15315155483910 -0.41550760681740    Biso  1.000000 Mo
   Mo6        1.0    0.27028250974960 -0.00950265650927 -0.41504518575560    Biso  1.000000 Mo
   Mo7        1.0    0.33333333333330 -0.33333333333330 -0.41485942848860    Biso  1.000000 Mo
   Mo8        1.0    0.39692195942890 0.34363839194370 -0.41605161332430    Biso  1.000000 Mo
   Mo9        1.0    0.43062811615320 0.18076733158640 -0.41517398629310    Biso  1.000000 Mo
   Mo10       1.0    0.46102722990220 0.01938464246002 -0.41521528869490    Biso  1.000000 Mo
   Mo11       1.0    -0.41125383421220 0.37576676909230 -0.41500071659260    Biso  1.000000 Mo
```

22. $MoTe_2/MoTe_2$-27.8

```
_cell_length_a                      12.322935
_cell_length_b                      12.322935
_cell_length_c                      40.000000
_cell_angle_alpha                   90.000000
_cell_angle_beta                    90.000000
_cell_angle_gamma                   120.000000
_cell_volume                        5260.402140
_space_group_name_H-M_alt           'P 3 1 2'
_space_group_IT_number              149

loop_
_space_group_symop_operation_xyz
   'x, y, z'
   '-y, x-y, z'
   '-x+y, -x, z'
   '-y, -x, -z'
   '-x+y, y, -z'
   'x, x-y, -z'

loop_
   _atom_site_label
   _atom_site_occupancy
   _atom_site_fract_x
   _atom_site_fract_y
   _atom_site_fract_z
   _atom_site_adp_type
   _atom_site_B_iso_or_equiv
   _atom_site_type_symbol
   Te1        1.0  -0.1977802212475 -0.4858153524133 0.4588557600085  Biso  1.000000 Te
   Te2        1.0  0.1793742184780 0.0451427503647 0.4587630529323  Biso  1.000000 Te
   Te3        1.0  -0.4364008577667 -0.4121339353442 0.4607868375851  Biso  1.000000 Te
   Te4        1.0  0.2558220995507 0.3577043514694 0.4606060155807  Biso  1.000000 Te
   Te5        1.0  0.3333333333333 -0.3333333333333 0.4560957669925  Biso  1.000000 Te
   Te6        1.0  -0.2017482656294 -0.4872005610328 0.3725711860789  Biso  1.000000 Te
   Te7        1.0  0.1803276678035 0.0472710850187 0.3724620638106  Biso  1.000000 Te
   Te8        1.0  -0.4350698796381 -0.4104484303633 0.3705155035067  Biso  1.000000 Te
   Te9        1.0  0.2556031017535 0.3580323751869 0.3704734068079  Biso  1.000000 Te
   Te10       1.0  0.3333333333333 -0.3333333333333 0.3745317741253  Biso  1.000000 Te
   Mo1        1.0  -0.3333333333333 0.3333333333333 0.4162175237488  Biso  1.000000 Mo
   Mo2        1.0  0.3634619994689 0.1022085068628 0.4159307689917  Biso  1.000000 Mo
   Mo3        1.0  0.0459683774568 -0.1353979269130 0.4156602758306  Biso  1.000000 Mo
   Mo4        1.0  0.4326182325263 0.4165601395580 0.4154185266596  Biso  1.000000 Mo
   Mo5        1.0  -0.4751122184536 -0.2791753385581 0.4157106020080  Biso  1.000000 Mo
```

23. $MoTe_2/MoTe_2$-32.2

```
_cell_length_a                        12.322671
_cell_length_b                        12.322671
_cell_length_c                        40.000000
_cell_angle_alpha                     90.000000
_cell_angle_beta                      90.000000
_cell_angle_gamma                     120.000000
_cell_volume                          5260.176607
_space_group_name_H-M_alt             'P 3 2 1'
_space_group_IT_number                150

loop_
_space_group_symop_operation_xyz
   'x, y, z'
   '-y, x-y, z'
   '-x+y, -x, z'
   'y, x, -z'
   'x-y, -y, -z'
   '-x, -x+y, -z'

loop_
   _atom_site_label
   _atom_site_occupancy
   _atom_site_fract_x
   _atom_site_fract_y
   _atom_site_fract_z
   _atom_site_adp_type
   _atom_site_B_iso_or_equiv
   _atom_site_type_symbol
   Te1        1.0  0.28182655338730 -0.20866644319440 0.46025651827710  Biso  1.000000 Te
   Te2        1.0  -0.40709276899530 0.03315223704736 0.45732364628300  Biso  1.000000 Te
   Te3        1.0  -0.10050109729080 0.25518819115190 0.46043218849710  Biso  1.000000 Te
   Te4        1.0  0.12431012066320 0.18110329305550 0.45926285434820  Biso  1.000000 Te
   Te5        1.0  0.33333333333330 -0.33333333333330 -0.46118834332850  Biso  1.000000 Te
   Te6        1.0  0.28155604102790 -0.20651457700220 0.37113660362490  Biso  1.000000 Te
   Te7        1.0  -0.40921247681510 0.02986949669101 0.37372982165650  Biso  1.000000 Te
   Te8        1.0  -0.09994427246259 0.25703623225010 0.37078891728110  Biso  1.000000 Te
   Te9        1.0  0.12628610717530 0.18130088730530 0.37187023436070  Biso  1.000000 Te
   Te10       1.0  0.33333333333330 -0.33333333333330 -0.36979464491830  Biso  1.000000 Te
   Mo1        1.0  0.33333333333330 -0.33333333333330 0.41539078816480  Biso  1.000000 Mo
   Mo2        1.0  -0.36065806944700 -0.10616264891960 0.41590765281510  Biso  1.000000 Mo
   Mo3        1.0  -0.05673346969446 0.12529668980990 0.41556195283820  Biso  1.000000 Mo
   Mo4        1.0  0.49135460899970 0.27197605420140 0.41548250035800  Biso  1.000000 Mo
   Mo5        1.0  -0.43086971479300 -0.40240273011580 0.41598895689910  Biso  1.000000 Mo
```

24. $MoTe_2/MoTe_2$-60

```
_cell_length_a                          3.417713
_cell_length_b                          3.417713
_cell_length_c                          40.000000
_cell_angle_alpha                       90.000000
_cell_angle_beta                        90.000000
_cell_angle_gamma                       120.000000
_cell_volume                            404.633566
_space_group_name_H-M_alt               'P -3 m 1'
_space_group_IT_number                  164

loop_
_space_group_symop_operation_xyz
   'x, y, z'
   '-x, -y, -z'
   '-y, x-y, z'
   'y, -x+y, -z'
   '-x+y, -x, z'
   'x-y, x, -z'
   'y, x, -z'
   '-y, -x, z'
   'x-y, -y, -z'
   '-x+y, y, z'
   '-x, -x+y, -z'
   'x, x-y, z'

loop_
   _atom_site_label
   _atom_site_occupancy
   _atom_site_fract_x
   _atom_site_fract_y
   _atom_site_fract_z
   _atom_site_adp_type
   _atom_site_B_iso_or_equiv
   _atom_site_type_symbol
   Te1        1.0  0.3333333333333 -0.3333333333333 0.4630970686890  Biso  1.000000 Te
   Te2        1.0  0.3333333333333 -0.3333333333333 0.3753083642082  Biso  1.000000 Te
   Mo1        1.0  0.3333333333333 -0.3333333333333 -0.4192688039844  Biso  1.000000 Mo
```

25. $MoTe_2/MoTe_2$-87.8

```
_cell_length_a                       12.322701
_cell_length_b                       12.322701
_cell_length_c                       40.000000
_cell_angle_alpha                    90.000000
_cell_angle_beta                     90.000000
_cell_angle_gamma                    120.000000
_cell_volume                         5260.201847
_space_group_name_H-M_alt            'P 3 2 1'
_space_group_IT_number               150

loop_
_space_group_symop_operation_xyz
   'x, y, z'
   '-y, x-y, z'
   '-x+y, -x, z'
   'y, x, -z'
   'x-y, -y, -z'
   '-x, -x+y, -z'

loop_
   _atom_site_label
   _atom_site_occupancy
   _atom_site_fract_x
   _atom_site_fract_y
   _atom_site_fract_z
   _atom_site_adp_type
   _atom_site_B_iso_or_equiv
   _atom_site_type_symbol
   Te1        1.0  0.33333333333330 -0.33333333333330 -0.46118704770380  Biso  1.000000 Te
   Te2        1.0  -0.03313163605202 -0.44023964338230 0.45734308682490  Biso  1.000000 Te
   Te3        1.0  -0.49048873329340 -0.28177336638430 0.46025615816360  Biso  1.000000 Te
   Te4        1.0  0.05676382456313 -0.12432946994400 0.45926609618940  Biso  1.000000 Te
   Te5        1.0  0.35571003078600 0.10050941682030 0.46042937475860  Biso  1.000000 Te
   Te6        1.0  0.33333333333330 -0.33333333333330 -0.36981369125130  Biso  1.000000 Te
   Te7        1.0  -0.02985326689536 -0.43908806422170 0.37372323041900  Biso  1.000000 Te
   Te8        1.0  -0.48804564524320 -0.28151903075680 0.37114631017420  Biso  1.000000 Te
   Te9        1.0  0.05498466173378 -0.12630490078560 0.37187444077000  Biso  1.000000 Te
   Te10       1.0  0.35699825155430 0.09995122679795 0.37080521206040  Biso  1.000000 Te
   Mo1        1.0  0.49132454307050 0.27206078745120 0.41548989579380  Biso  1.000000 Mo
   Mo2        1.0  0.33333333333330 -0.33333333333330 0.41539208946960  Biso  1.000000 Mo
   Mo3        1.0  -0.12532349066480 -0.18200624868840 0.41556801362460  Biso  1.000000 Mo
   Mo4        1.0  -0.36063848554800 -0.10609477834280 0.41591217610180  Biso  1.000000 Mo
   Mo5        1.0  0.40244710575060 -0.02848245276018 0.41599729418950  Biso  1.000000 Mo
```

26. $MoTe_2$/$MoTe_2$-92.2

```
_cell_length_a                        12.322714
_cell_length_b                        12.322714
_cell_length_c                        40.000000
_cell_angle_alpha                     90.000000
_cell_angle_beta                      90.000000
_cell_angle_gamma                     120.000000
_cell_volume                          5260.213246
_space_group_name_H-M_alt             'P 3 1 2'
_space_group_IT_number                149

loop_
_space_group_symop_operation_xyz
   'x, y, z'
   '-y, x-y, z'
   '-x+y, -x, z'
   '-y, -x, -z'
   '-x+y, y, -z'
   'x, x-y, -z'

loop_
   _atom_site_label
   _atom_site_occupancy
   _atom_site_fract_x
   _atom_site_fract_y
   _atom_site_fract_z
   _atom_site_adp_type
   _atom_site_B_iso_or_equiv
   _atom_site_type_symbol
   Te1        1.0  0.35772828946110 0.25583301230490 0.46059022663490  Biso  1.000000 Te
   Te2        1.0  0.04512439115209 0.17935925406770 0.45875732189230  Biso  1.000000 Te
   Te3        1.0  0.43640215296480 0.02425465708284 0.46078481900660  Biso  1.000000 Te
   Te4        1.0  -0.48580650673510 -0.19782518294130 0.45884326872160  Biso  1.000000 Te
   Te5        1.0  -0.33333333333330 0.33333333333330 0.45608730051930  Biso  1.000000 Te
   Te6        1.0  0.35805258068250 0.25561188506190 0.37047001573050  Biso  1.000000 Te
   Te7        1.0  0.04724899037073 0.18030409512830 0.37244661498630  Biso  1.000000 Te
   Te8        1.0  0.43507582426690 0.02460889333704 0.37049744411920  Biso  1.000000 Te
   Te9        1.0  -0.48717775119050 -0.20177766867910 0.37255779870230  Biso  1.000000 Te
   Te10       1.0  -0.33333333333330 0.33333333333330 0.37451662819010  Biso  1.000000 Te
   Mo1        1.0  0.18135045654090 0.13540730354140 0.41564945501780  Biso  1.000000 Mo
   Mo2        1.0  -0.43264471020160 -0.01609555505410 0.41540798571710  Biso  1.000000 Mo
   Mo3        1.0  0.26126142578900 -0.10215816267130 0.41591903360390  Biso  1.000000 Mo
   Mo4        1.0  0.33333333333330 -0.33333333333330 0.41620223538270  Biso  1.000000 Mo
   Mo5        1.0  -0.27923105067210 -0.47514174519710 0.41569851746520  Biso  1.000000 Mo
```

27. $MoTe_2$/$MoTe_2$-120

```
_cell_length_a                         3.417716
_cell_length_b                         3.417716
_cell_length_c                         40.000000
_cell_angle_alpha                      90.000000
_cell_angle_beta                       90.000000
_cell_angle_gamma                      120.000000
_cell_volume                           404.634187
_space_group_name_H-M_alt              'P -6 m 2'
_space_group_IT_number                 187

loop_
_space_group_symop_operation_xyz
   'x, y, z'
   '-y, x-y, z'
   '-x+y, -x, z'
   'x, y, -z'
   '-y, x-y, -z'
   '-x+y, -x, -z'
   '-y, -x, z'
   '-x+y, y, z'
   'x, x-y, z'
   '-y, -x, -z'
   '-x+y, y, -z'
   'x, x-y, -z'

loop_
   _atom_site_label
   _atom_site_occupancy
   _atom_site_fract_x
   _atom_site_fract_y
   _atom_site_fract_z
   _atom_site_adp_type
   _atom_site_B_iso_or_equiv
   _atom_site_type_symbol
   Te1        1.0  -0.3333333333333 0.3333333333333 -0.4530733722373  Biso  1.000000 Te
   Te2        1.0  -0.3333333333333 0.3333333333333 -0.3652628624299  Biso  1.000000 Te
   Mo1        1.0  0.3333333333333 -0.3333333333333 -0.4092143738577  Biso  1.000000 Mo
```

28. WS_2/WS_2-0

```
_cell_length_a                        3.097672
_cell_length_b                        3.097672
_cell_length_c                        40.000000
_cell_angle_alpha                     90.000000
_cell_angle_beta                      90.000000
_cell_angle_gamma                     120.000000
_cell_volume                          332.400406
_space_group_name_H-M_alt             'P -6 m 2'
_space_group_IT_number                187

loop_
_space_group_symop_operation_xyz
   'x, y, z'
   '-y, x-y, z'
   '-x+y, -x, z'
   'x, y, -z'
   '-y, x-y, -z'
   '-x+y, -x, -z'
   '-y, -x, z'
   '-x+y, y, z'
   'x, x-y, z'
   '-y, -x, -z'
   '-x+y, y, -z'
   'x, x-y, -z'

loop_
   _atom_site_label
   _atom_site_occupancy
   _atom_site_fract_x
   _atom_site_fract_y
   _atom_site_fract_z
   _atom_site_adp_type
   _atom_site_B_iso_or_equiv
   _atom_site_type_symbol
   W1         1.0     0.3333333333333     0.666666666667     -0.4210801846536    Biso  1.000000 W
   S1         1.0     0.0000000000000     0.000000000000     -0.3821930680137    Biso  1.000000 S
   S2         1.0     0.0000000000000     0.000000000000     -0.4597565312003    Biso  1.000000 S
```

29. WS_2/WS_2-17.9

```
_cell_length_a                        17.247150
_cell_length_b                        17.247150
_cell_length_c                        40.000000
_cell_angle_alpha                     90.000000
_cell_angle_beta                      90.000000
_cell_angle_gamma                     120.000000
_cell_volume                          10304.462075
_space_group_name_H-M_alt             'P 3 1 2'
_space_group_IT_number                149

loop_
_space_group_symop_operation_xyz
   'x, y, z'
   '-y, x-y, z'
   '-x+y, -x, z'
   '-y, -x, -z'
   '-x+y, y, -z'
   'x, x-y, -z'

loop_
   _atom_site_label
   _atom_site_occupancy
   _atom_site_fract_x
   _atom_site_fract_y
   _atom_site_fract_z
   _atom_site_adp_type
   _atom_site_B_iso_or_equiv
   _atom_site_type_symbol
   W1         1.0    0.11841638328820 0.07632924950658 -0.42553137584460    Biso  1.000000 W
   W2         1.0    0.15151600197020 -0.08566456696134 -0.42613144797850    Biso  1.000000 W
   W3         1.0    0.18199827181220 -0.24835973579950 -0.42592067576350    Biso  1.000000 W
   W4         1.0    0.21458773424520 -0.40961301493440 -0.42560407596490    Biso  1.000000 W
   W5         1.0    0.27932580063870 0.26927982297460 -0.42589537476010    Biso  1.000000 W
   W6         1.0    0.31162103330860 0.10757374853470 -0.42637517387420    Biso  1.000000 W
   W7         1.0    0.34388130108530 -0.05374939558018 -0.42659594132560    Biso  1.000000 W
   W8         1.0    0.44130595086250 0.46202275013840 -0.42603107311930    Biso  1.000000 W
   W9         1.0    0.47191748140720 0.29963674859740 -0.42578248039450    Biso  1.000000 W
   W10        1.0    -0.49426198932060 0.13927006870920 -0.42611191177210    Biso  1.000000 W
   W11        1.0    -0.33333333333330 0.33333333333330 -0.42541067545430    Biso  1.000000 W
   S1         1.0    0.07651111038983 -0.04248788094210 -0.38677271448850    Biso  1.000000 S
   S2         1.0    0.10812428253690 -0.20390052598460 -0.38702180750690    Biso  1.000000 S
   S3         1.0    0.13889059336290 -0.36658448686790 -0.38688020990480    Biso  1.000000 S
   S4         1.0    0.17151579129000 0.47186254509460 -0.38710901623920    Biso  1.000000 S
   S5         1.0    0.23717946866110 0.15111123110210 -0.38697079581660    Biso  1.000000 S
   S6         1.0    0.26952516309450 -0.01035656425338 -0.38746887973280    Biso  1.000000 S
   S7         1.0    0.33333333333330 -0.33333333333330 -0.38661193876510    Biso  1.000000 S
   S8         1.0    0.39759109906520 0.34381892112480 -0.38693445202690    Biso  1.000000 S
   S9         1.0    0.42953693924700 0.18138285797510 -0.38714002746280    Biso  1.000000 S
   S10        1.0    0.46175519797960 0.02116384968006 -0.38732942088540    Biso  1.000000 S
   S11        1.0    -0.40950534449080 0.37602556211270 -0.38681342694790    Biso  1.000000 S
   S12        1.0    0.07687686507717 -0.04092887966522 -0.46382066941860    Biso  1.000000 S
   S13        1.0    0.10802424869570 -0.20453494681790 -0.46491102711370    Biso  1.000000 S
   S14        1.0    0.13860131892980 -0.36796114223690 -0.46444515264870    Biso  1.000000 S
   S15        1.0    0.17394853815930 0.47267146438590 -0.46466033951380    Biso  1.000000 S
   S16        1.0    0.23585204462930 0.15221895671080 -0.46466391284120    Biso  1.000000 S
   S17        1.0    0.26902743156540 -0.00989144435881 -0.46544704856670    Biso  1.000000 S
   S18        1.0    0.33333333333330 -0.33333333333330 -0.46328309718420    Biso  1.000000 S
   S19        1.0    0.39706965100160 0.34287445863430 -0.46486494909610    Biso  1.000000 S
   S20        1.0    0.43047272271740 0.18162738270010 -0.46483059629870    Biso  1.000000 S
   S21        1.0    0.46303905458750 0.02133647758624 -0.46524132008950    Biso  1.000000 S
   S22        1.0    -0.41062002245410 0.37385086699540 -0.46387187839430    Biso  1.000000 S
```

30. WS_2/WS_2-27.8

```
_cell_length_a                       11.168926
_cell_length_b                       11.168926
_cell_length_c                       40.000000
_cell_angle_alpha                    90.000000
_cell_angle_beta                     90.000000
_cell_angle_gamma                    120.000000
_cell_volume                         4321.290558
_space_group_name_H-M_alt            'P 3 1 2'
_space_group_IT_number               149

loop_
_space_group_symop_operation_xyz
   'x, y, z'
   '-y, x-y, z'
   '-x+y, -x, z'
   '-y, -x, -z'
   '-x+y, y, -z'
   'x, x-y, -z'

loop_
   _atom_site_label
   _atom_site_occupancy
   _atom_site_fract_x
   _atom_site_fract_y
   _atom_site_fract_z
   _atom_site_adp_type
   _atom_site_B_iso_or_equiv
   _atom_site_type_symbol
   W1         1.0    -0.1526677348292  0.4626002269868 -0.4258411586844    Biso  1.000000 W
   W2         1.0     0.3835521416901 -0.1539733934956 -0.4258450861165    Biso  1.000000 W
   W3         1.0    -0.0776651892874  0.2312661551934 -0.4257395965325    Biso  1.000000 W
   W4         1.0    -0.3070540835302 -0.0766158124242 -0.4259972108483    Biso  1.000000 W
   W5         1.0     0.0000000000000  0.0000000000000 -0.4260437580707    Biso  1.000000 W
   S1         1.0    -0.3333333333333  0.3333333333333 -0.3870137706570    Biso  1.000000 S
   S2         1.0     0.4361147478927  0.0253505305100 -0.3867736343310    Biso  1.000000 S
   S3         1.0     0.2041005015756 -0.2828029681285 -0.3869964133939    Biso  1.000000 S
   S4         1.0    -0.2565841130694  0.1019971780339 -0.3868417334216    Biso  1.000000 S
   S5         1.0     0.0507815569876  0.1792202927049 -0.3870197367245    Biso  1.000000 S
   S6         1.0    -0.3333333333333  0.3333333333333 -0.4634299451016    Biso  1.000000 S
   S7         1.0     0.4355859164847  0.0249496031838 -0.4648404688038    Biso  1.000000 S
   S8         1.0     0.2028619844268 -0.2845897571408 -0.4643176595874    Biso  1.000000 S
   S9         1.0    -0.2552292751729  0.1029995205507 -0.4648914374628    Biso  1.000000 S
   S10        1.0     0.0484507358337  0.1798465648647 -0.4643905611859    Biso  1.000000 S
```

31. WS_2/WS_2-32.2

```
_cell_length_a                         11.168883
_cell_length_b                         11.168883
_cell_length_c                         40.000000
_cell_angle_alpha                      90.000000
_cell_angle_beta                       90.000000
_cell_angle_gamma                      120.000000
_cell_volume                           4321.257349
_space_group_name_H-M_alt              'P 3 2 1'
_space_group_IT_number                 150

loop_
_space_group_symop_operation_xyz
   'x, y, z'
   '-y, x-y, z'
   '-x+y, -x, z'
   'y, x, -z'
   'x-y, -y, -z'
   '-x, -x+y, -z'

loop_
   _atom_site_label
   _atom_site_occupancy
   _atom_site_fract_x
   _atom_site_fract_y
   _atom_site_fract_z
   _atom_site_adp_type
   _atom_site_B_iso_or_equiv
   _atom_site_type_symbol
   W1         1.0     -0.28062662719060 -0.48738507080100 -0.4256803619098    Biso  1.000000 W
   W2         1.0      0.40955973906050  0.43552238078570 -0.4260395774990    Biso  1.000000 W
   W3         1.0      0.10292625868080  0.35934950246500 -0.4259310375537    Biso  1.000000 W
   W4         1.0     -0.12734471524920  0.05226527335797 -0.4258284313169    Biso  1.000000 W
   W5         1.0      0.33333333333330 -0.33333333333330 -0.4257648066678    Biso  1.000000 W
   S1         1.0      0.33333333333330 -0.33333333333330  0.3865858634496    Biso  1.000000 S
   S2         1.0      0.35840539739000  0.25681792808060 -0.3868516646110    Biso  1.000000 S
   S3         1.0      0.05224110254764  0.18009024995500 -0.3869140865430    Biso  1.000000 S
   S4         1.0     -0.48705532363560 -0.20507620650480 -0.3869446286762    Biso  1.000000 S
   S5         1.0      0.43623683373750  0.02593336982557 -0.3870401005015    Biso  1.000000 S
   S6         1.0      0.33333333333330 -0.33333333333330  0.4647721995774    Biso  1.000000 S
   S7         1.0      0.35745844548670  0.25548340904140 -0.4648129718012    Biso  1.000000 S
   S8         1.0      0.05391677540894  0.18040594858700 -0.4644646472128    Biso  1.000000 S
   S9         1.0     -0.48899869515330 -0.20679666187760 -0.4648374773376    Biso  1.000000 S
   S10        1.0      0.43715841893210  0.02872095438773 -0.4638479095534    Biso  1.000000 S
```

32. WS_2/WS_2-42.1

```
_cell_length_a                         17.247101
_cell_length_b                         17.247101
_cell_length_c                         40.000000
_cell_angle_alpha                      90.000000
_cell_angle_beta                       90.000000
_cell_angle_gamma                      120.000000
_cell_volume                           10304.402818
_space_group_name_H-M_alt              'P 3 2 1'
_space_group_IT_number                 150

loop_
_space_group_symop_operation_xyz
   'x, y, z'
   '-y, x-y, z'
   '-x+y, -x, z'
   'y, x, -z'
   'x-y, -y, -z'
   '-x, -x+y, -z'

loop_
   _atom_site_label
   _atom_site_occupancy
   _atom_site_fract_x
   _atom_site_fract_y
   _atom_site_fract_z
   _atom_site_adp_type
   _atom_site_B_iso_or_equiv
   _atom_site_type_symbol
   W1         1.0    0.1179928996124 0.0434660374082 -0.4257815047808    Biso  1.000000 W
   W2         1.0    0.3771932415304 -0.4076913955307 -0.4256900416551    Biso  1.000000 W
   W3         1.0    0.3446849811962 0.3984182371409 -0.4252863202265    Biso  1.000000 W
   W4         1.0    0.3110871192881 0.2038344281916 -0.4256190801851    Biso  1.000000 W
   W5         1.0    0.2796116409592 0.0108765814783 -0.4265154826516    Biso  1.000000 W
   W6         1.0    0.2472116255783 -0.1833940213192 -0.4260275156041    Biso  1.000000 W
   W7         1.0    0.1497121821986 0.2368489899936 -0.4256931703588    Biso  1.000000 W
   W8         1.0    -0.4618671592907 -0.4406584628672 -0.4262069510130    Biso  1.000000 W
   W9         1.0    -0.4947832594116 0.3644438220483 -0.4256411424738    Biso  1.000000 W
   W10        1.0    0.4733863917924 0.1716101493509 -0.4261758485035    Biso  1.000000 W
   W11        1.0    0.3333333333333 -0.3333333333333 0.4258531768579    Biso  1.000000 W
   S1         1.0    0.0436020941442 -0.0747899328449 -0.3868014511497    Biso  1.000000 S
   S2         1.0    0.3013549433304 0.4739154702377 -0.3867232073069    Biso  1.000000 S
   S3         1.0    0.2682222346615 0.2794464359866 -0.3865892441057    Biso  1.000000 S
   S4         1.0    0.2363480953966 0.0852915276800 -0.3870522855191    Biso  1.000000 S
   S5         1.0    0.2055145977715 -0.1068428601450 -0.3871733698249    Biso  1.000000 S
   S6         1.0    0.1391831149104 -0.4954854338152 -0.3870552376937    Biso  1.000000 S
   S7         1.0    0.4635292449416 0.4413449826761 -0.3867788622838    Biso  1.000000 S
   S8         1.0    0.4298021918555 0.2460686537660 -0.3868869018892    Biso  1.000000 S
   S9         1.0    0.3974528122660 0.0536424390003 -0.3874157094198    Biso  1.000000 S
   S10        1.0    0.3333333333333 -0.3333333333333 -0.3867109748524    Biso  1.000000 S
   S11        1.0    -0.2151713008852 0.3762103831952 -0.3869527539995    Biso  1.000000 S
   S12        1.0    0.0442825028634 -0.0743357595016 -0.4646151316754    Biso  1.000000 S
   S13        1.0    0.3036525895903 0.4749033933724 -0.4641259896307    Biso  1.000000 S
   S14        1.0    0.2683861649703 0.2805736605855 -0.4634235157326    Biso  1.000000 S
   S15        1.0    0.2348060623098 0.0863173941669 -0.4646975652207    Biso  1.000000 S
   S16        1.0    0.2046341877391 -0.1086041003313 -0.4648722066619    Biso  1.000000 S
   S17        1.0    0.1400582555673 -0.4945974806589 -0.4648491505063    Biso  1.000000 S
   S18        1.0    0.4625750560564 0.4391921579179 -0.4638780808284    Biso  1.000000 S
   S19        1.0    0.4285630939664 0.2457424590274 -0.4642792682036    Biso  1.000000 S
   S20        1.0    0.3982214703980 0.0542957127055 -0.4653133781639    Biso  1.000000 S
   S21        1.0    0.3333333333333 -0.3333333333333 -0.4647298105139    Biso  1.000000 S
   S22        1.0    -0.2157531337259 0.3772712965961 -0.4648198806936    Biso  1.000000 S
```

33. WS_2/WS_2-60

```
_cell_length_a                        3.097674
_cell_length_b                        3.097674
_cell_length_c                        40.000000
_cell_angle_alpha                     90.000000
_cell_angle_beta                      90.000000
_cell_angle_gamma                     120.000000
_cell_volume                          332.400867
_space_group_name_H-M_alt             'P -3 m 1'
_space_group_IT_number                164

loop_
_space_group_symop_operation_xyz
   'x, y, z'
   '-x, -y, -z'
   '-y, x-y, z'
   'y, -x+y, -z'
   '-x+y, -x, z'
   'x-y, x, -z'
   'y, x, -z'
   '-y, -x, z'
   'x-y, -y, -z'
   '-x+y, y, z'
   '-x, -x+y, -z'
   'x, x-y, z'

loop_
   _atom_site_label
   _atom_site_occupancy
   _atom_site_fract_x
   _atom_site_fract_y
   _atom_site_fract_z
   _atom_site_adp_type
   _atom_site_B_iso_or_equiv
   _atom_site_type_symbol
   W1         1.0     0.333333333333     0.666666666667     -0.4286638654307    Biso  1.000000 W
   S1         1.0     0.333333333333     0.666666666667    0.3897672337391    Biso  1.000000 S
   S2         1.0     0.333333333333     0.666666666667    0.4673471172335    Biso  1.000000 S
```

34. WS_2/WS_2-77.9

```
_cell_length_a                         17.247168
_cell_length_b                         17.247168
_cell_length_c                         40.000000
_cell_angle_alpha                      90.000000
_cell_angle_beta                       90.000000
_cell_angle_gamma                      120.000000
_cell_volume                           10304.482587
_space_group_name_H-M_alt              'P 3 2 1'
_space_group_IT_number                 150

loop_
_space_group_symop_operation_xyz
   'x, y, z'
   '-y, x-y, z'
   '-x+y, -x, z'
   'y, x, -z'
   'x-y, -y, -z'
   '-x, -x+y, -z'

loop_
   _atom_site_label
   _atom_site_occupancy
   _atom_site_fract_x
   _atom_site_fract_y
   _atom_site_fract_z
   _atom_site_adp_type
   _atom_site_B_iso_or_equiv
   _atom_site_type_symbol
   W1         1.0     0.1834463022487 -0.2472186164789 -0.4260577890142    Biso  1.000000 W
   W2         1.0     0.4076571787674 -0.3772507572960 -0.4257617848107    Biso  1.000000 W
   W3         1.0     -0.3643594110383 0.4947641292922 -0.4256580377589    Biso  1.000000 W
   W4         1.0     0.4407041332048 0.4618264762301 -0.4262796296070    Biso  1.000000 W
   W5         1.0     0.3333333333333 -0.3333333333333 0.4258686052840    Biso  1.000000 W
   W6         1.0     0.0872334094706 0.2368764452449 -0.4257659427326    Biso  1.000000 W
   W7         1.0     -0.1071937236539 0.2037900523629 -0.4256920989469    Biso  1.000000 W
   W8         1.0     0.1179530397135 0.0744389157624 -0.4259226771160    Biso  1.000000 W
   W9         1.0     -0.2687328953448 0.0108541690317 -0.4266026262857    Biso  1.000000 W
   W10        1.0     0.3447821370816 -0.0536480220588 -0.4253224686277    Biso  1.000000 W
   W11        1.0     -0.1715964648932 -0.4733265876744 -0.4262126023033    Biso  1.000000 W
   S1         1.0     0.1068494791504 -0.2055203701044 -0.3872157171596    Biso  1.000000 S
   S2         1.0     0.3333333333333 -0.3333333333333 -0.3867672540046    Biso  1.000000 S
   S3         1.0     -0.4413045120420 -0.4635791896921 -0.3867947036874    Biso  1.000000 S
   S4         1.0     0.3653846687910 -0.4955051319859 -0.3870854436173    Biso  1.000000 S
   S5         1.0     -0.2151744528465 0.4086706138199 -0.3869472633870    Biso  1.000000 S
   S6         1.0     0.0112493726289 0.2794228634924 -0.3866294870863    Biso  1.000000 S
   S7         1.0     -0.1836800878493 0.2459727182078 -0.3868962840339    Biso  1.000000 S
   S8         1.0     0.2362060195948 0.1509380547481 -0.3871380317503    Biso  1.000000 S
   S9         1.0     0.0436741514844 0.1183516340671 -0.3868913918850    Biso  1.000000 S
   S10        1.0     -0.3438023589139 0.0535770233314 -0.3874670116120    Biso  1.000000 S
   S11        1.0     0.3014818582986 -0.1724943670375 -0.3867257331304    Biso  1.000000 S
   S12        1.0     0.1086743510976 -0.2047777948886 -0.4649458315752    Biso  1.000000 S
   S13        1.0     0.3333333333333 -0.3333333333333 -0.4648480942221    Biso  1.000000 S
   S14        1.0     -0.4390176360749 -0.4627071631084 -0.4639565731227    Biso  1.000000 S
   S15        1.0     0.3652796920248 -0.4946995510863 -0.4649359445315    Biso  1.000000 S
   S16        1.0     -0.2158249770898 0.4069398522430 -0.4648578689243    Biso  1.000000 S
   S17        1.0     0.0123430907516 0.2806713603346 -0.4634634158001    Biso  1.000000 S
   S18        1.0     -0.1828081458507 0.2456277524944 -0.4643328299573    Biso  1.000000 S
   S19        1.0     0.2346915134943 0.1484325304773 -0.4648474233914    Biso  1.000000 S
   S20        1.0     0.0444843730084 0.1187583703829 -0.4647758425000    Biso  1.000000 S
   S21        1.0     -0.3438748329772 0.0543467935138 -0.4654200855751    Biso  1.000000 S
   S22        1.0     0.3038093615415 -0.1711296953173 -0.4641790138512    Biso  1.000000 S
```

35. WS_2/WS_2-87.8

```
_cell_length_a                        11.168821
_cell_length_b                        11.168821
_cell_length_c                        40.000000
_cell_angle_alpha                     90.000000
_cell_angle_beta                      90.000000
_cell_angle_gamma                     120.000000
_cell_volume                          4321.209382
_space_group_name_H-M_alt             'P 3 2 1'
_space_group_IT_number                150

loop_
_space_group_symop_operation_xyz
   'x, y, z'
   '-y, x-y, z'
   '-x+y, -x, z'
   'y, x, -z'
   'x-y, -y, -z'
   '-x, -x+y, -z'

loop_
   _atom_site_label
   _atom_site_occupancy
   _atom_site_fract_x
   _atom_site_fract_y
   _atom_site_fract_z
   _atom_site_adp_type
   _atom_site_B_iso_or_equiv
   _atom_site_type_symbol
   W1         1.0     0.2806349527165 -0.2067557418141 0.4257148246392    Biso  1.000000 W
   W2         1.0    -0.4095612852674  0.0259651490068 0.4260679213126    Biso  1.000000 W
   W3         1.0     0.1273475252966  0.1795950068282 0.4258492111526    Biso  1.000000 W
   W4         1.0    -0.1029202995740  0.2564062181580 0.4259602987711    Biso  1.000000 W
   W5         1.0     0.3333333333333 -0.3333333333333   -0.4258080333318    Biso  1.000000 W
   S1         1.0     0.1015972720686 -0.2568273960281 0.3868806395916    Biso  1.000000 S
   S2         1.0     0.4102878458278 -0.0259387898962 0.3870682349583    Biso  1.000000 S
   S3         1.0    -0.0522366250433  0.1278594061216 0.3869342704270    Biso  1.000000 S
   S4         1.0    -0.2819742223919  0.2050984087357 0.3869817387925    Biso  1.000000 S
   S5         1.0     0.3333333333333 -0.3333333333333 0.3866195710280    Biso  1.000000 S
   S6         1.0     0.1019541621955 -0.2554654178143 0.4648360319675    Biso  1.000000 S
   S7         1.0     0.4084579695737 -0.0286858236889 0.4638632269843    Biso  1.000000 S
   S8         1.0    -0.0539075371877  0.1264624625706 0.4644931653861    Biso  1.000000 S
   S9         1.0    -0.2822013812416  0.2067967972631 0.4648784065552    Biso  1.000000 S
   S10        1.0     0.3333333333333 -0.3333333333333 0.4648203747953    Biso  1.000000 S
```

36. WS_2/WS_2-102.1

```
_cell_length_a                          17.247238
_cell_length_b                          17.247238
_cell_length_c                          40.000000
_cell_angle_alpha                       90.000000
_cell_angle_beta                        90.000000
_cell_angle_gamma                       120.000000
_cell_volume                            10304.566915
_space_group_name_H-M_alt               'P 3 1 2'
_space_group_IT_number                  149

loop_
_space_group_symop_operation_xyz
   'x, y, z'
   '-y, x-y, z'
   '-x+y, -x, z'
   '-y, -x, -z'
   '-x+y, y, -z'
   'x, x-y, -z'

loop_
   _atom_site_label
   _atom_site_occupancy
   _atom_site_fract_x
   _atom_site_fract_y
   _atom_site_fract_z
   _atom_site_adp_type
   _atom_site_B_iso_or_equiv
   _atom_site_type_symbol
   W1          1.0    -0.4096240739792 0.3757807156273 -0.4255748058985    Biso  1.000000 W
   W2          1.0    -0.0537618555681 -0.3976400626164 -0.4265549331317    Biso  1.000000 W
   W3          1.0    -0.2483444887253 -0.4303488016825 -0.4258985685479    Biso  1.000000 W
   W4          1.0    0.4942569383115 -0.1392873090457 -0.4260738662873    Biso  1.000000 W
   W5          1.0    0.2996551409938 -0.1722637911989 -0.4257601029951    Biso  1.000000 W
   W6          1.0    0.1075787265158 -0.2040522685601 -0.4263478471970    Biso  1.000000 W
   W7          1.0    0.0420727084488 0.1184118847916 -0.4255296849959    Biso  1.000000 W
   W8          1.0    -0.1515134931185 0.0856600518314 -0.4261128468393    Biso  1.000000 W
   W9          1.0    0.4620098439163 0.0207104052114 -0.4259986944316    Biso  1.000000 W
   W10         1.0    0.0100454536971 0.2793362074414 -0.4258800337263    Biso  1.000000 W
   W11         1.0    0.3333333333333 -0.3333333333333 -0.4253806754702    Biso  1.000000 W
   S1          1.0    0.4718631912092 0.3003267944216 -0.3870730483005    Biso  1.000000 S
   S2          1.0    -0.3665863434319 0.4945344240158 -0.3868637663942    Biso  1.000000 S
   S3          1.0    0.3760109950672 -0.2144790442224 -0.3867787253817    Biso  1.000000 S
   S4          1.0    0.1814069559401 -0.2481266328750 -0.3871099064894    Biso  1.000000 S
   S5          1.0    -0.0103528928079 -0.2798589874265 -0.3874382995937    Biso  1.000000 S
   S6          1.0    -0.0765072803737 0.0424681938024 -0.3867667412530    Biso  1.000000 S
   S7          1.0    -0.4617637115085 -0.0211719500236 -0.3872894596277    Biso  1.000000 S
   S8          1.0    0.3438014099681 -0.0537759049682 -0.3869079273427    Biso  1.000000 S
   S9          1.0    0.0860411342886 0.2371493222994 -0.3869603978015    Biso  1.000000 S
   S10         1.0    -0.1081147873445 0.2038966796819 -0.3870042512408    Biso  1.000000 S
   S11         1.0    -0.3333333333333 0.3333333333333 -0.3865822023280    Biso  1.000000 S
   S12         1.0    0.4726691332668 0.2987431897331 -0.4646374953087    Biso  1.000000 S
   S13         1.0    -0.3679478301650 0.4934227250976 -0.4644127026853    Biso  1.000000 S
   S14         1.0    0.3738652288826 -0.2155309156588 -0.4638500739106    Biso  1.000000 S
   S15         1.0    0.1816582134630 -0.2488354039177 -0.4648015973244    Biso  1.000000 S
   S16         1.0    -0.0098823404381 -0.2789376137719 -0.4654110640537    Biso  1.000000 S
   S17         1.0    -0.0769035868844 0.0409229745999 -0.4638178677079    Biso  1.000000 S
   S18         1.0    -0.4630602030418 -0.0213465740754 -0.4651855484344    Biso  1.000000 S
   S19         1.0    0.3429081153071 -0.0541873136695 -0.4648517553703    Biso  1.000000 S
   S20         1.0    0.0836543935162 0.2358916571151 -0.4646465607546    Biso  1.000000 S
   S21         1.0    -0.1080179534939 0.2045394328488 -0.4648958355714    Biso  1.000000 S
   S22         1.0    -0.3333333333333 0.3333333333333 -0.4632706608542    Biso  1.000000 S
```

37. WS_2/WS_2-120

```
_cell_length_a                          3.097666
_cell_length_b                          3.097666
_cell_length_c                          40.000000
_cell_angle_alpha                       90.000000
_cell_angle_beta                        90.000000
_cell_angle_gamma                       120.000000
_cell_volume                            332.399025
_space_group_name_H-M_alt               'P -6 m 2'
_space_group_IT_number                  187

loop_
_space_group_symop_operation_xyz
   'x, y, z'
   '-y, x-y, z'
   '-x+y, -x, z'
   'x, y, -z'
   '-y, x-y, -z'
   '-x+y, -x, -z'
   '-y, -x, z'
   '-x+y, y, z'
   'x, x-y, z'
   '-y, -x, -z'
   '-x+y, y, -z'
   'x, x-y, -z'

loop_
   _atom_site_label
   _atom_site_occupancy
   _atom_site_fract_x
   _atom_site_fract_y
   _atom_site_fract_z
   _atom_site_adp_type
   _atom_site_B_iso_or_equiv
   _atom_site_type_symbol
   W1         1.0     0.666666666667     0.333333333333     -0.4210803108231    Biso  1.000000 W
   S1         1.0     0.333333333333     0.666666666667     -0.3821931192055    Biso  1.000000 S
   S2         1.0     0.333333333333     0.666666666667     -0.4597566103381    Biso  1.000000 S
```

38. WSe_2/WSe_2-0

```
_cell_length_a                        3.199075
_cell_length_b                        3.199075
_cell_length_c                        40.000000
_cell_angle_alpha                     90.000000
_cell_angle_beta                      90.000000
_cell_angle_gamma                     120.000000
_cell_volume                          354.518957
_space_group_name_H-M_alt             'P -6 m 2'
_space_group_IT_number                187

loop_
_space_group_symop_operation_xyz
   'x, y, z'
   '-y, x-y, z'
   '-x+y, -x, z'
   'x, y, -z'
   '-y, x-y, -z'
   '-x+y, -x, -z'
   '-y, -x, z'
   '-x+y, y, z'
   'x, x-y, z'
   '-y, -x, -z'
   '-x+y, y, -z'
   'x, x-y, -z'

loop_
   _atom_site_label
   _atom_site_occupancy
   _atom_site_fract_x
   _atom_site_fract_y
   _atom_site_fract_z
   _atom_site_adp_type
   _atom_site_B_iso_or_equiv
   _atom_site_type_symbol
   W1         1.0     0.3333333333333 -0.3333333333333 -0.4170579974483    Biso  1.000000 W
   Se1        1.0     -0.3333333333333 0.3333333333333 -0.4584143956761    Biso  1.000000 Se
   Se2        1.0     -0.3333333333333 0.3333333333333 -0.3755250231845    Biso  1.000000 Se
```

39. WSe_2/WSe_2-17.9

```
_cell_length_a                        17.811750
_cell_length_b                        17.811750
_cell_length_c                        40.000000
_cell_angle_alpha                     90.000000
_cell_angle_beta                      90.000000
_cell_angle_gamma                     120.000000
_cell_volume                          10990.155185
_space_group_name_H-M_alt             'P 3 1 2'
_space_group_IT_number                149

loop_
_space_group_symop_operation_xyz
   'x, y, z'
   '-y, x-y, z'
   '-x+y, -x, z'
   '-y, -x, -z'
   '-x+y, y, -z'
   'x, x-y, -z'

loop_
   _atom_site_label
   _atom_site_occupancy
   _atom_site_fract_x
   _atom_site_fract_y
   _atom_site_fract_z
   _atom_site_adp_type
   _atom_site_B_iso_or_equiv
   _atom_site_type_symbol
   W1          1.0    0.1185060944111 0.0766380964823 -0.4213229315329    Biso  1.000000 W
   W2          1.0    0.1517240161845 -0.0857136058238 -0.4217942229924    Biso  1.000000 W
   W3          1.0    0.1817456018578 -0.2486078729821 -0.4216148425978    Biso  1.000000 W
   W4          1.0    0.2144043889131 -0.4098965463930 -0.4213686759895    Biso  1.000000 W
   W5          1.0    0.2794063456014 0.2694663578538 -0.4215659420924    Biso  1.000000 W
   W6          1.0    0.3114594502220 0.1075191629172 -0.4220051305085    Biso  1.000000 W
   W7          1.0    0.3438672632851 -0.0536679062535 -0.4221990425712    Biso  1.000000 W
   W8          1.0    0.4414112026045 0.4619071174758 -0.4216826115766    Biso  1.000000 W
   W9          1.0    0.4715555867007 0.2993868899920 -0.4214718503524    Biso  1.000000 W
   W10         1.0    -0.4942268170228 0.1392070596182 -0.4217825814084    Biso  1.000000 W
   W11         1.0    -0.3333333333333 0.3333333333333 -0.4211828581504    Biso  1.000000 W
   Se1         1.0    0.0772408984550 -0.0408254631574 -0.4622082413467    Biso  1.000000 Se
   Se2         1.0    0.1080052665127 -0.2045539315559 -0.4632529461176    Biso  1.000000 Se
   Se3         1.0    0.1383892351677 -0.3681789012076 -0.4628007277041    Biso  1.000000 Se
   Se4         1.0    0.1737249269660 0.4722624031630 -0.4629947446784    Biso  1.000000 Se
   Se5         1.0    0.2361106832878 0.1523820277972 -0.4630375422356    Biso  1.000000 Se
   Se6         1.0    0.2691486191623 -0.0098721812030 -0.4637042393166    Biso  1.000000 Se
   Se7         1.0    0.3333333333333 -0.3333333333333 -0.4616919677687    Biso  1.000000 Se
   Se8         1.0    0.3971315740308 0.3429376751756 -0.4632512870929    Biso  1.000000 Se
   Se9         1.0    0.4302448516182 0.1813749570760 -0.4631273506011    Biso  1.000000 Se
   Se10        1.0    0.4629129775359 0.0213098363871 -0.4635073519715    Biso  1.000000 Se
   Se11        1.0    -0.4108341264972 0.3738372259197 -0.4622263749021    Biso  1.000000 Se
   Se12        1.0    0.0766424264437 -0.0422474810574 -0.3799826917765    Biso  1.000000 Se
   Se13        1.0    0.1080638658936 -0.2040317639548 -0.3800709046708    Biso  1.000000 Se
   Se14        1.0    0.1388499517085 -0.3667875659582 -0.3800036610534    Biso  1.000000 Se
   Se15        1.0    0.1717392603818 0.4718600002322 -0.3801988229042    Biso  1.000000 Se
   Se16        1.0    0.2370151777875 0.1512354102877 -0.3800748877394    Biso  1.000000 Se
   Se17        1.0    0.2694795811142 -0.0103249476942 -0.3804296132137    Biso  1.000000 Se
   Se18        1.0    0.3333333333333 -0.3333333333333 -0.3798987746490    Biso  1.000000 Se
   Se19        1.0    0.3975515299540 0.3437648773052 -0.3799600881577    Biso  1.000000 Se
   Se20        1.0    0.4295816163809 0.1813725322916 -0.3802075929046    Biso  1.000000 Se
   Se21        1.0    0.4618672596726 0.0211968772283 -0.3803290721190    Biso  1.000000 Se
   Se22        1.0    -0.4096669649016 0.3758033907857 -0.3799818817086    Biso  1.000000 Se
```

40. WSe_2/WSe_2-27.8

```
_cell_length_a                        11.534430
_cell_length_b                        11.534430
_cell_length_c                        40.000000
_cell_angle_alpha                     90.000000
_cell_angle_beta                      90.000000
_cell_angle_gamma                     120.000000
_cell_volume                          4608.746965
_space_group_name_H-M_alt             'P 3 1 2'
_space_group_IT_number                149

loop_
_space_group_symop_operation_xyz
   'x, y, z'
   '-y, x-y, z'
   '-x+y, -x, z'
   '-y, -x, -z'
   '-x+y, y, -z'
   'x, x-y, -z'

loop_
   _atom_site_label
   _atom_site_occupancy
   _atom_site_fract_x
   _atom_site_fract_y
   _atom_site_fract_z
   _atom_site_adp_type
   _atom_site_B_iso_or_equiv
   _atom_site_type_symbol
   W1         1.0    -0.1794385735805 -0.1296688894912 0.4215631210170    Biso  1.000000 W
   W2         1.0    -0.4851489628963 -0.2034040599995 0.4215740202235    Biso  1.000000 W
   W3         1.0    -0.1029122197087 -0.3599624288058 0.4217119561215    Biso  1.000000 W
   W4         1.0    -0.4114126222574 -0.4350989312875 0.4214531780699    Biso  1.000000 W
   W5         1.0    -0.3333333333333 0.3333333333333 0.4217334155503    Biso  1.000000 W
   Se1        1.0    -0.1308250560725 0.0484829868329 0.4626692459353    Biso  1.000000 Se
   Se2        1.0    -0.4361841065117 -0.0248785804040 0.4633105701291    Biso  1.000000 Se
   Se3        1.0    0.2558743232917 -0.1023049941202 0.4632717998098    Biso  1.000000 Se
   Se4        1.0    0.3333333333333 -0.3333333333333 0.4616997203939    Biso  1.000000 Se
   Se5        1.0    -0.2851920917302 -0.4869427191913 0.4627174769092    Biso  1.000000 Se
   Se6        1.0    -0.1295115718012 0.0502578645909 0.3801417899987    Biso  1.000000 Se
   Se7        1.0    -0.4353985271874 -0.0251977718995 0.3798959964513    Biso  1.000000 Se
   Se8        1.0    0.2558614125807 -0.1027266712533 0.3798362545598    Biso  1.000000 Se
   Se9        1.0    0.3333333333333 -0.3333333333333 0.3803523734829    Biso  1.000000 Se
   Se10       1.0    -0.2828903380439 -0.4873594563001 0.3801677115484    Biso  1.000000 Se
```

41. WSe_2/WSe_2-32.2

```
_cell_length_a                        11.534231
_cell_length_b                        11.534231
_cell_length_c                        40.000000
_cell_angle_alpha                     90.000000
_cell_angle_beta                      90.000000
_cell_angle_gamma                     120.000000
_cell_volume                          4608.588448
_space_group_name_H-M_alt             'P 3 2 1'
_space_group_IT_number                150

loop_
_space_group_symop_operation_xyz
   'x, y, z'
   '-y, x-y, z'
   '-x+y, -x, z'
   'y, x, -z'
   'x-y, -y, -z'
   '-x, -x+y, -z'

loop_
   _atom_site_label
   _atom_site_occupancy
   _atom_site_fract_x
   _atom_site_fract_y
   _atom_site_fract_z
   _atom_site_adp_type
   _atom_site_B_iso_or_equiv
   _atom_site_type_symbol
   W1          1.0    -0.1032342135170 -0.3595449364171 -0.4216625775098    Biso  1.000000 W
   W2          1.0    0.1270939370300 -0.0526822288090 -0.4215825617327    Biso  1.000000 W
   W3          1.0    -0.4089479441885 -0.4351703713778 -0.4217426463912    Biso  1.000000 W
   W4          1.0    0.2798070653190 0.4876020748927 -0.4214651355400    Biso  1.000000 W
   W5          1.0    0.3333333333333 -0.3333333333333 0.4214296713694    Biso  1.000000 W
   Se1         1.0    -0.0541244109367 -0.1806100419195 -0.4628579832978    Biso  1.000000 Se
   Se2         1.0    0.4085148174278 0.4374330645413 -0.4621487331414    Biso  1.000000 Se
   Se3         1.0    -0.3572352788237 -0.2556847178235 -0.4632557351896    Biso  1.000000 Se
   Se4         1.0    0.3333333333333 -0.3333333333333 -0.4633185527276    Biso  1.000000 Se
   Se5         1.0    -0.2068069165126 0.2822112467398 -0.4632093853523    Biso  1.000000 Se
   Se6         1.0    -0.0524864027263 -0.1801563672393 -0.3800784847607    Biso  1.000000 Se
   Se7         1.0    0.4100754790162 0.4364859043114 -0.3803113355401    Biso  1.000000 Se
   Se8         1.0    -0.3582174208240 -0.2566646830031 -0.3799377199205    Biso  1.000000 Se
   Se9         1.0    0.3333333333333 -0.3333333333333 -0.3796970018725    Biso  1.000000 Se
   Se10        1.0    -0.2052589209325 0.2819526766256 -0.3799750737474    Biso  1.000000 Se
```

42. WSe_2/WSe_2-42.1

```
_cell_length_a                         17.811781
_cell_length_b                         17.811781
_cell_length_c                         40.000000
_cell_angle_alpha                      90.000000
_cell_angle_beta                       90.000000
_cell_angle_gamma                      120.000000
_cell_volume                           10990.192845
_space_group_name_H-M_alt              'P 3 2 1'
_space_group_IT_number                 150

loop_
_space_group_symop_operation_xyz
   'x, y, z'
   '-y, x-y, z'
   '-x+y, -x, z'
   'y, x, -z'
   'x-y, -y, -z'
   '-x, -x+y, -z'

loop_
   _atom_site_label
   _atom_site_occupancy
   _atom_site_fract_x
   _atom_site_fract_y
   _atom_site_fract_z
   _atom_site_adp_type
   _atom_site_B_iso_or_equiv
   _atom_site_type_symbol
   W1          1.0    0.1178406988998 0.0742839563689 0.4215607192416    Biso  1.000000 W
   W2          1.0    0.3773992788168 -0.2151936328987 0.4214726709285    Biso  1.000000 W
   W3          1.0    0.3449586682827 -0.0536385729569 0.4211675895726    Biso  1.000000 W
   W4          1.0    0.3108457453258 0.1071921053065 0.4214362344695    Biso  1.000000 W
   W5          1.0    0.2796196577678 0.2687448835788 0.4222027097763    Biso  1.000000 W
   W6          1.0    0.2471686153257 0.4307792277611 0.4217784446995    Biso  1.000000 W
   W7          1.0    0.1493611849648 -0.0874696489777 0.4214873976265    Biso  1.000000 W
   W8          1.0    -0.4616968286152 -0.0210799410539 0.4219499976407    Biso  1.000000 W
   W9          1.0    -0.4946922061346 0.1412128714311 0.4213802211800    Biso  1.000000 W
   W10         1.0    0.4734288715706 0.3019600015760 0.4218318452697    Biso  1.000000 W
   W11         1.0    0.3333333333333 -0.3333333333333 -0.4214894233764    Biso  1.000000 W
   Se1         1.0    0.0444579684664 0.1186493489691 0.4631107670383    Biso  1.000000 Se
   Se2         1.0    0.3038794378091 -0.1712777410051 0.4626100195636    Biso  1.000000 Se
   Se3         1.0    0.2681146915649 -0.0124299902072 0.4618800926837    Biso  1.000000 Se
   Se4         1.0    0.2346080131197 0.1486236036106 0.4631443633723    Biso  1.000000 Se
   Se5         1.0    0.2050316256565 0.3135432704018 0.4632592733329    Biso  1.000000 Se
   Se6         1.0    0.1398335279535 -0.3653620055569 0.4632724374297    Biso  1.000000 Se
   Se7         1.0    0.4631303705410 0.0240137870972 0.4623585800525    Biso  1.000000 Se
   Se8         1.0    0.4284385299313 0.1831099588655 0.4626672168192    Biso  1.000000 Se
   Se9         1.0    0.3980895770008 0.3437716576075 0.4636654819098    Biso  1.000000 Se
   Se10        1.0    0.3333333333333 -0.3333333333333 0.4632469586264    Biso  1.000000 Se
   Se11        1.0    -0.2159058576632 0.4070611483011 0.4631808043790    Biso  1.000000 Se
   Se12        1.0    0.0437404858639 0.1184233109425 0.3799312270028    Biso  1.000000 Se
   Se13        1.0    0.3017389525568 -0.1723417208883 0.3799301260673    Biso  1.000000 Se
   Se14        1.0    0.2681787106614 -0.0114251714628 0.3798906169179    Biso  1.000000 Se
   Se15        1.0    0.2360913210197 0.1507748523703 0.3801923756424    Biso  1.000000 Se
   Se16        1.0    0.2055167554928 0.3125513504237 0.3802847176634    Biso  1.000000 Se
   Se17        1.0    0.1392117025887 -0.3653405864197 0.3801720261141    Biso  1.000000 Se
   Se18        1.0    0.4635608443286 0.0224625198623 0.3799993248115    Biso  1.000000 Se
   Se19        1.0    0.4294854948754 0.1836265484676 0.3800199292492    Biso  1.000000 Se
   Se20        1.0    0.3974326757479 0.3438327220870 0.3804435173346    Biso  1.000000 Se
   Se21        1.0    0.3333333333333 -0.3333333333333 0.3798164626416    Biso  1.000000 Se
   Se22        1.0    -0.2152259185862 0.4085031304466 0.3799626656537    Biso  1.000000 Se
```

43. WSe_2/WSe_2-60

```
_cell_length_a                          3.199067
_cell_length_b                          3.199067
_cell_length_c                          40.000000
_cell_angle_alpha                       90.000000
_cell_angle_beta                        90.000000
_cell_angle_gamma                       120.000000
_cell_volume                            354.517160
_space_group_name_H-M_alt               'P -3 m 1'
_space_group_IT_number                  164

loop_
_space_group_symop_operation_xyz
   'x, y, z'
   '-x, -y, -z'
   '-y, x-y, z'
   'y, -x+y, -z'
   '-x+y, -x, z'
   'x-y, x, -z'
   'y, x, -z'
   '-y, -x, z'
   'x-y, -y, -z'
   '-x+y, y, z'
   '-x, -x+y, -z'
   'x, x-y, z'

loop_
   _atom_site_label
   _atom_site_occupancy
   _atom_site_fract_x
   _atom_site_fract_y
   _atom_site_fract_z
   _atom_site_adp_type
   _atom_site_B_iso_or_equiv
   _atom_site_type_symbol
   W1         1.0     0.3333333333333 -0.3333333333333 -0.4241347702748    Biso  1.000000 W
   Se1        1.0     0.3333333333333 -0.3333333333333 0.4654709949152    Biso  1.000000 Se
   Se2        1.0     0.3333333333333 -0.3333333333333 0.3826140762964    Biso  1.000000 Se
```

44. WSe_2/WSe_2-77.9

```
_cell_length_a                         17.811657
_cell_length_b                         17.811657
_cell_length_c                         40.000000
_cell_angle_alpha                      90.000000
_cell_angle_beta                       90.000000
_cell_angle_gamma                      120.000000
_cell_volume                           10990.039853
_space_group_name_H-M_alt              'P 3 2 1'
_space_group_IT_number                 150

loop_
_space_group_symop_operation_xyz
   'x, y, z'
   '-y, x-y, z'
   '-x+y, -x, z'
   'y, x, -z'
   'x-y, -y, -z'
   '-x, -x+y, -z'

loop_
   _atom_site_label
   _atom_site_occupancy
   _atom_site_fract_x
   _atom_site_fract_y
   _atom_site_fract_z
   _atom_site_adp_type
   _atom_site_B_iso_or_equiv
   _atom_site_type_symbol
   W1         1.0    0.0742827609410 -0.0435571532435 0.4215513487068    Biso  1.000000 W
   W2         1.0    -0.0874740734771 -0.2368266616499 0.4214830894161    Biso  1.000000 W
   W3         1.0    -0.2471601608232 -0.4307725738673 0.4217802923979    Biso  1.000000 W
   W4         1.0    -0.4074094664404 0.3773960673228 0.4214734487425    Biso  1.000000 W
   W5         1.0    0.2687404963005 -0.0108799930271 0.4222004624492    Biso  1.000000 W
   W6         1.0    0.1071845406203 -0.2036607339969 0.4214392124093    Biso  1.000000 W
   W7         1.0    -0.0536406308337 -0.3986030703020 0.4211715287744    Biso  1.000000 W
   W8         1.0    0.4616996561642 0.0210814721902 0.4219534980161    Biso  1.000000 W
   W9         1.0    0.3019556679271 -0.1714658923480 0.4218348071066    Biso  1.000000 W
   W10        1.0    0.1412094880661 -0.3640947523258 0.4213858895197    Biso  1.000000 W
   W11        1.0    0.3333333333333 -0.3333333333333 0.4214912142198    Biso  1.000000 W
   Se1        1.0    -0.0444527604227 -0.1186437087561 0.4631028697618    Biso  1.000000 Se
   Se2        1.0    -0.2050236837821 -0.3135277147466 0.4632553384192    Biso  1.000000 Se
   Se3        1.0    -0.3653548842079 0.4948008118593 0.4632724070564    Biso  1.000000 Se
   Se4        1.0    0.4751589333563 0.3038811039475 0.4626124251440    Biso  1.000000 Se
   Se5        1.0    0.1486173433227 -0.0859860300093 0.4631384606875    Biso  1.000000 Se
   Se6        1.0    -0.0124424254348 -0.2805461443127 0.4618832985397    Biso  1.000000 Se
   Se7        1.0    0.3333333333333 -0.3333333333333 -0.4632464606011    Biso  1.000000 Se
   Se8        1.0    0.3437749192708 -0.0543125100966 0.4636648623535    Biso  1.000000 Se
   Se9        1.0    0.1831021827695 -0.2453343553277 0.4626713393091    Biso  1.000000 Se
   Se10       1.0    0.0240027055985 -0.4391147757652 0.4623590564664    Biso  1.000000 Se
   Se11       1.0    0.3770260579199 -0.2159085534435 0.4631820745104    Biso  1.000000 Se
   Se12       1.0    -0.0437364788194 -0.1184272690380 0.3799202544008    Biso  1.000000 Se
   Se13       1.0    -0.2055203149178 -0.3125531564410 0.3802816110625    Biso  1.000000 Se
   Se14       1.0    -0.3653403416777 0.4954440271244 0.3801744217599    Biso  1.000000 Se
   Se15       1.0    0.4740784057438 0.3017386020336 0.3799310932341    Biso  1.000000 Se
   Se16       1.0    0.1507775886236 -0.0853282684275 0.3801872752473    Biso  1.000000 Se
   Se17       1.0    -0.0114291448106 -0.2796144583015 0.3798908110147    Biso  1.000000 Se
   Se18       1.0    0.3333333333333 -0.3333333333333 -0.3798179243150    Biso  1.000000 Se
   Se19       1.0    0.3438400402586 -0.0535997200205 0.3804447164182    Biso  1.000000 Se
   Se20       1.0    0.1836216275398 -0.2458650284929 0.3800228936207    Biso  1.000000 Se
   Se21       1.0    0.0224600984885 -0.4411004310610 0.3800026197226    Biso  1.000000 Se
   Se22       1.0    0.3762684285300 -0.2152223612326 0.3799661989582    Biso  1.000000 Se
```

45. WSe_2/WSe_2-87.8

```
_cell_length_a                          11.534231
_cell_length_b                          11.534231
_cell_length_c                          40.000000
_cell_angle_alpha                       90.000000
_cell_angle_beta                        90.000000
_cell_angle_gamma                       120.000000
_cell_volume                            4608.588448
_space_group_name_H-M_alt               'P 3 2 1'
_space_group_IT_number                  150

loop_
_space_group_symop_operation_xyz
   'x, y, z'
   '-y, x-y, z'
   '-x+y, -x, z'
   'y, x, -z'
   'x-y, -y, -z'
   '-x, -x+y, -z'

loop_
   _atom_site_label
   _atom_site_occupancy
   _atom_site_fract_x
   _atom_site_fract_y
   _atom_site_fract_z
   _atom_site_adp_type
   _atom_site_B_iso_or_equiv
   _atom_site_type_symbol
   W1          1.0    0.1270945953669 -0.0526808125160 -0.4215820752280    Biso  1.000000 W
   W2          1.0    -0.1032346010031 -0.3595445715805 -0.4216630210700    Biso  1.000000 W
   W3          1.0    0.3333333333333 -0.3333333333333 0.4214309908677    Biso  1.000000 W
   W4          1.0    0.4351797180523 0.0262271527947 -0.4217423073880    Biso  1.000000 W
   W5          1.0    0.2077935457090 -0.2798076541526 -0.4214640502946    Biso  1.000000 W
   Se1         1.0    -0.0541235321485 -0.1806078362433 -0.4628577239343    Biso  1.000000 Se
   Se2         1.0    -0.2822148853413 -0.4890173455334 -0.4632102369520    Biso  1.000000 Se
   Se3         1.0    0.2556826621076 -0.1015569652300 -0.4632540459386    Biso  1.000000 Se
   Se4         1.0    0.0289207437921 -0.4085162362082 -0.4621488729564    Biso  1.000000 Se
   Se5         1.0    0.3333333333333 -0.3333333333333 -0.4633179713898    Biso  1.000000 Se
   Se6         1.0    -0.0524857538633 -0.1801568268420 -0.3800784434982    Biso  1.000000 Se
   Se7         1.0    -0.2819535031464 -0.4872146544508 -0.3799757095668    Biso  1.000000 Se
   Se8         1.0    0.2566660915513 -0.1015516376951 -0.3799371421235    Biso  1.000000 Se
   Se9         1.0    0.0264063336658 -0.4100760401701 -0.3803108668357    Biso  1.000000 Se
   Se10        1.0    0.3333333333333 -0.3333333333333 -0.3796962029035    Biso  1.000000 Se
```

46. WSe_2/WSe_2-102.1

```
_cell_length_a                         17.811726
_cell_length_b                         17.811726
_cell_length_c                         40.000000
_cell_angle_alpha                      90.000000
_cell_angle_beta                       90.000000
_cell_angle_gamma                      120.000000
_cell_volume                           10990.124587
_space_group_name_H-M_alt              'P 3 1 2'
_space_group_IT_number                 149

loop_
_space_group_symop_operation_xyz
   'x, y, z'
   '-y, x-y, z'
   '-x+y, -x, z'
   '-y, -x, -z'
   '-x+y, y, -z'
   'x, x-y, -z'

loop_
   _atom_site_label
   _atom_site_occupancy
   _atom_site_fract_x
   _atom_site_fract_y
   _atom_site_fract_z
   _atom_site_adp_type
   _atom_site_B_iso_or_equiv
   _atom_site_type_symbol
   W1          1.0    -0.3438719815593 0.0536672054370 0.4222031917791    Biso  1.000000 W
   W2          1.0    0.4619006506738 0.0204932966492 0.4216850127563    Biso  1.000000 W
   W3          1.0    0.2374399628041 0.1517254334993 0.4217981559643    Biso  1.000000 W
   W4          1.0    0.2694656134903 -0.0099403979925 0.4215652922767    Biso  1.000000 W
   W5          1.0    0.0418663923880 0.1185077321982 0.4213306229017    Biso  1.000000 W
   W6          1.0    -0.2143993441432 0.4098946789568 0.4213743018408    Biso  1.000000 W
   W7          1.0    -0.1817515486258 0.2486034636904 0.4216183089777    Biso  1.000000 W
   W8          1.0    0.3333333333333 -0.3333333333333 0.4211826725635    Biso  1.000000 W
   W9          1.0    0.3665581390949 -0.4942302667836 0.4217855466698    Biso  1.000000 W
   W10         1.0    0.1075114663079 -0.2039435202256 0.4220101370581    Biso  1.000000 W
   W11         1.0    0.1721573970649 0.4715437491788 0.4214716135593    Biso  1.000000 W
   Se1         1.0    -0.2691468755106 0.0098788869155 0.4637059376224    Biso  1.000000 Se
   Se2         1.0    -0.4629242619466 -0.0213159637611 0.4635092968331    Biso  1.000000 Se
   Se3         1.0    0.3125589784892 0.1080054802061 0.4632549077069    Biso  1.000000 Se
   Se4         1.0    0.3429373291267 -0.0541841960896 0.4632503447826    Biso  1.000000 Se
   Se5         1.0    0.0837321147217 0.2361266729186 0.4630391271096    Biso  1.000000 Se
   Se6         1.0    0.1180682274485 0.0772334002360 0.4622148711814    Biso  1.000000 Se
   Se7         1.0    -0.1383985335908 0.3681619729206 0.4628027050286    Biso  1.000000 Se
   Se8         1.0    -0.3333333333333 0.3333333333333 0.4617031088688    Biso  1.000000 Se
   Se9         1.0    -0.2985551714799 0.1737214458399 0.4629995641285    Biso  1.000000 Se
   Se10        1.0    0.4108318665109 -0.3738414784551 0.4622241587337    Biso  1.000000 Se
   Se11        1.0    0.1813837167263 -0.2488607980035 0.4631257469113    Biso  1.000000 Se
   Se12        1.0    -0.2694765952291 0.0103307681096 0.3804329974331    Biso  1.000000 Se
   Se13        1.0    -0.4618693229822 -0.0212003429724 0.3803314036215    Biso  1.000000 Se
   Se14        1.0    0.3120874259243 0.1080606979877 0.3800745706608    Biso  1.000000 Se
   Se15        1.0    0.3437679739043 -0.0537817844642 0.3799589844489    Biso  1.000000 Se
   Se16        1.0    0.0857870577611 0.2370131486162 0.3800774944790    Biso  1.000000 Se
   Se17        1.0    0.1188866271783 0.0766430882291 0.3799898058627    Biso  1.000000 Se
   Se18        1.0    -0.1388555237924 0.3667801085058 0.3800059839725    Biso  1.000000 Se
   Se19        1.0    -0.3333333333333 0.3333333333333 0.3799063416326    Biso  1.000000 Se
   Se20        1.0    -0.3001293246449 0.1717332935864 0.3802017973569    Biso  1.000000 Se
   Se21        1.0    0.4096684279345 -0.3758038890731 0.3799825408974    Biso  1.000000 Se
   Se22        1.0    0.1813692697116 -0.2482032019328 0.3802085338700    Biso  1.000000 Se
```

47. WSe_2/WSe_2-120

```
_cell_length_a                       3.199065
_cell_length_b                       3.199065
_cell_length_c                       40.000000
_cell_angle_alpha                    90.000000
_cell_angle_beta                     90.000000
_cell_angle_gamma                    120.000000
_cell_volume                         354.516790
_space_group_name_H-M_alt            'P -6 m 2'
_space_group_IT_number               187

loop_
_space_group_symop_operation_xyz
   'x, y, z'
   '-y, x-y, z'
   '-x+y, -x, z'
   'x, y, -z'
   '-y, x-y, -z'
   '-x+y, -x, -z'
   '-y, -x, z'
   '-x+y, y, z'
   'x, x-y, z'
   '-y, -x, -z'
   '-x+y, y, -z'
   'x, x-y, -z'

loop_
   _atom_site_label
   _atom_site_occupancy
   _atom_site_fract_x
   _atom_site_fract_y
   _atom_site_fract_z
   _atom_site_adp_type
   _atom_site_B_iso_or_equiv
   _atom_site_type_symbol
   W1         1.0     -0.3333333333333 0.3333333333333 0.4170578841617    Biso  1.000000 W
   Se1        1.0     0.3333333333333 -0.3333333333333 0.4584143743489    Biso  1.000000 Se
   Se2        1.0     0.3333333333333 -0.3333333333333 0.3755248186562    Biso  1.000000 Se
```

48. WTe_2/WTe_2-0

```
_cell_length_a                        3.404189
_cell_length_b                        3.404189
_cell_length_c                        40.000000
_cell_angle_alpha                     90.000000
_cell_angle_beta                      90.000000
_cell_angle_gamma                     120.000000
_cell_volume                          401.437593
_space_group_name_H-M_alt             'P -6 m 2'
_space_group_IT_number                187

loop_
_space_group_symop_operation_xyz
   'x, y, z'
   '-y, x-y, z'
   '-x+y, -x, z'
   'x, y, -z'
   '-y, x-y, -z'
   '-x+y, -x, -z'
   '-y, -x, z'
   '-x+y, y, z'
   'x, x-y, z'
   '-y, -x, -z'
   '-x+y, y, -z'
   'x, x-y, -z'

loop_
   _atom_site_label
   _atom_site_occupancy
   _atom_site_fract_x
   _atom_site_fract_y
   _atom_site_fract_z
   _atom_site_adp_type
   _atom_site_B_iso_or_equiv
   _atom_site_type_symbol
   Te1        1.0     0.3333333333333 -0.3333333333333 0.4531482444738    Biso  1.000000 Te
   Te2        1.0     0.3333333333333 -0.3333333333333 0.3645151009125    Biso  1.000000 Te
   W1         1.0     -0.3333333333333 0.3333333333333 0.4088830827695    Biso  1.000000 W
```

49. WTe_2/WTe_2-17.9

```
_cell_length_a                      18.953699
_cell_length_b                      18.953699
_cell_length_c                      40.000000
_cell_angle_alpha                   90.000000
_cell_angle_beta                    90.000000
_cell_angle_gamma                   120.000000
_cell_volume                        12444.532520
_space_group_name_H-M_alt           'P 3 1 2'
_space_group_IT_number              149

loop_
_space_group_symop_operation_xyz
   'x, y, z'
   '-y, x-y, z'
   '-x+y, -x, z'
   '-y, -x, -z'
   '-x+y, y, -z'
   'x, x-y, -z'

loop_
   _atom_site_label
   _atom_site_occupancy
   _atom_site_fract_x
   _atom_site_fract_y
   _atom_site_fract_z
   _atom_site_adp_type
   _atom_site_B_iso_or_equiv
   _atom_site_type_symbol
   Te1        1.0    0.07833774287150 -0.04002588530965 -0.4578355369563    Biso  1.000000 Te
   Te2        1.0    0.10804660016560 -0.20466398256520 -0.4590334722734    Biso  1.000000 Te
   Te3        1.0    0.13758423587680 -0.36897588994010 -0.4586439170747    Biso  1.000000 Te
   Te4        1.0    0.17393307921580 0.47144533978780 -0.4588529038172    Biso  1.000000 Te
   Te5        1.0    0.23647548109590 0.15321701471780 -0.4588804102076    Biso  1.000000 Te
   Te6        1.0    0.26937677692250 -0.00956247258047 -0.4599151635559    Biso  1.000000 Te
   Te7        1.0    0.33333333333330 -0.33333333333330 -0.4569890782993    Biso  1.000000 Te
   Te8        1.0    0.39678274216210 0.34274070659720 -0.4591903416238    Biso  1.000000 Te
   Te9        1.0    0.42995177230290 0.18061391310140 -0.4589934916421    Biso  1.000000 Te
   Te10       1.0    0.46293197027000 0.02105509243273 -0.4594113066914    Biso  1.000000 Te
   Te11       1.0    -0.41210160525710 0.37291701690960 -0.4578717362315    Biso  1.000000 Te
   Te12       1.0    0.07754102286267 -0.04151692074809 -0.3704281561241    Biso  1.000000 Te
   Te13       1.0    0.10825119299210 -0.20389825187060 -0.3700855669120    Biso  1.000000 Te
   Te14       1.0    0.13828613533870 -0.36753329806920 -0.3700290718729    Biso  1.000000 Te
   Te15       1.0    0.17165232052950 0.47112407522930 -0.3703649447629    Biso  1.000000 Te
   Te16       1.0    0.23723091203560 0.15165847287870 -0.3701540873701    Biso  1.000000 Te
   Te17       1.0    0.26976660800610 -0.01012077487544 -0.3702778975305    Biso  1.000000 Te
   Te18       1.0    0.33333333333330 -0.33333333333330 -0.3706770049804    Biso  1.000000 Te
   Te19       1.0    0.39736115181860 0.34359080862150 -0.3697307307995    Biso  1.000000 Te
   Te20       1.0    0.42929969347310 0.18057106233330 -0.3703484086195    Biso  1.000000 Te
   Te21       1.0    0.46165174264220 0.02107289187656 -0.3704244251850    Biso  1.000000 Te
   Te22       1.0    -0.41049822147770 0.37514701212660 -0.3704233737500    Biso  1.000000 Te
   W1         1.0    0.11899590473160 0.07796051461234 -0.4143220039349    Biso  1.000000 W
   W2         1.0    0.15303142187340 -0.08555557565568 -0.4147852787496    Biso  1.000000 W
   W3         1.0    0.18093230012540 -0.24963641014060 -0.4144967722424    Biso  1.000000 W
   W4         1.0    0.21302975186010 -0.41118869900520 -0.4143649860066    Biso  1.000000 W
   W5         1.0    0.27965387013360 0.27008224811030 -0.4143853707461    Biso  1.000000 W
   W6         1.0    0.31085554789840 0.10696998143840 -0.4149975054051    Biso  1.000000 W
   W7         1.0    0.34356049148250 -0.05336235810966 -0.4152629882633    Biso  1.000000 W
   W8         1.0    0.44161493205430 0.46124959818380 -0.4145474114979    Biso  1.000000 W
   W9         1.0    0.46971108123710 0.29811112010510 -0.4143716990216    Biso  1.000000 W
   W10        1.0    -0.49412351580770 0.13882761303890 -0.4148262908925    Biso  1.000000 W
   W11        1.0    -0.33333333333330 0.33333333333330 -0.4142458978652    Biso  1.000000 W
```

50. WTe_2/WTe_2-27.8

```
_cell_length_a                         12.273998
_cell_length_b                         12.273998
_cell_length_c                         40.000000
_cell_angle_alpha                      90.000000
_cell_angle_beta                       90.000000
_cell_angle_gamma                      120.000000
_cell_volume                           5218.704878
_space_group_name_H-M_alt              'P 3 1 2'
_space_group_IT_number                 149

loop_
_space_group_symop_operation_xyz
   'x, y, z'
   '-y, x-y, z'
   '-x+y, -x, z'
   '-y, -x, -z'
   '-x+y, y, -z'
   'x, x-y, -z'

loop_
   _atom_site_label
   _atom_site_occupancy
   _atom_site_fract_x
   _atom_site_fract_y
   _atom_site_fract_z
   _atom_site_adp_type
   _atom_site_B_iso_or_equiv
   _atom_site_type_symbol
   Te1        1.0   -0.1348521351073 0.0456803673468 -0.4583745439368    Biso  1.000000 Te
   Te2        1.0   0.1026631072221 0.3573075723505 -0.4602497004224    Biso  1.000000 Te
   Te3        1.0   0.3333333333333 -0.3333333333333 -0.4557324292779    Biso  1.000000 Te
   Te4        1.0   -0.4351278365944 -0.0242768109161 -0.4600850602288    Biso  1.000000 Te
   Te5        1.0   -0.1995091095045 0.2878195645037 -0.4583216604036    Biso  1.000000 Te
   Te6        1.0   -0.1316100573185 0.0478583379178 -0.3711506205943    Biso  1.000000 Te
   Te7        1.0   0.1015817179403 0.3578753339521 -0.3692543888512    Biso  1.000000 Te
   Te8        1.0   0.3333333333333 -0.3333333333333 -0.3732140886208    Biso  1.000000 Te
   Te9        1.0   -0.4357532268850 -0.0247098688264 -0.3692291158786    Biso  1.000000 Te
   Te10       1.0   -0.2007407211071 0.2859307045477 -0.3710596199194    Biso  1.000000 Te
   W1         1.0   -0.3164375313365 -0.0827497800379 -0.4145475285087    Biso  1.000000 W
   W2         1.0   -0.0728716708435 0.2308318211067 -0.4149598896950    Biso  1.000000 W
   W3         1.0   0.1425559069226 -0.4701841992596 -0.4148379276712    Biso  1.000000 W
   W4         1.0   -0.3795444948999 0.1523630300064 -0.4147540834537    Biso  1.000000 W
   W5         1.0   0.0000000000000 0.0000000000000 -0.4151743321791    Biso  1.000000 W
```

51. WTe_2/WTe_2-32.2

```
_cell_length_a                          12.273987
_cell_length_b                          12.273987
_cell_length_c                          40.000000
_cell_angle_alpha                       90.000000
_cell_angle_beta                        90.000000
_cell_angle_gamma                       120.000000
_cell_volume                            5218.695146
_space_group_name_H-M_alt               'P 3 2 1'
_space_group_IT_number                  150

loop_
_space_group_symop_operation_xyz
   'x, y, z'
   '-y, x-y, z'
   '-x+y, -x, z'
   'y, x, -z'
   'x-y, -y, -z'
   '-x, -x+y, -z'

loop_
   _atom_site_label
   _atom_site_occupancy
   _atom_site_fract_x
   _atom_site_fract_y
   _atom_site_fract_z
   _atom_site_adp_type
   _atom_site_B_iso_or_equiv
   _atom_site_type_symbol
   Te1        1.0   -0.2814977080407 -0.4903534451986 -0.4599465473212    Biso  1.000000 Te
   Te2        1.0   -0.0565331552408 -0.1812075347381 -0.4589624228607    Biso  1.000000 Te
   Te3        1.0   0.0327490986444 -0.4073953746319 -0.4569805246430    Biso  1.000000 Te
   Te4        1.0   0.2553538630470 -0.1002584515303 -0.4600895647478    Biso  1.000000 Te
   Te5        1.0   0.3333333333333 -0.3333333333333 -0.4609233791921    Biso  1.000000 Te
   Te6        1.0   -0.2813181680894 -0.4881520421280 -0.3699366665414    Biso  1.000000 Te
   Te7        1.0   -0.0548460611268 -0.1812926452296 -0.3707029050767    Biso  1.000000 Te
   Te8        1.0   0.0299988079036 -0.4091705203430 -0.3726179461845    Biso  1.000000 Te
   Te9        1.0   0.2568977193054 -0.0998650640599 -0.3696680814505    Biso  1.000000 Te
   Te10       1.0   0.3333333333333 -0.3333333333333 -0.3686638573096    Biso  1.000000 Te
   W1         1.0   0.3333333333333 -0.3333333333333 0.4146746359615    Biso  1.000000 W
   W2         1.0   -0.1058416275848 -0.3605644388744 -0.4151341599683    Biso  1.000000 W
   W3         1.0   0.1252718683825 -0.0563519308415 -0.4148433397990    Biso  1.000000 W
   W4         1.0   -0.0287217949756 0.4024197270076 -0.4152092031691    Biso  1.000000 W
   W5         1.0   0.2189690157621 -0.2721661256504 -0.4147938614880    Biso  1.000000 W
```

52. WTe_2/WTe_2-42.1

```
_cell_length_a                       18.953699
_cell_length_b                       18.953699
_cell_length_c                       40.000000
_cell_angle_alpha                    90.000000
_cell_angle_beta                     90.000000
_cell_angle_gamma                    120.000000
_cell_volume                         12444.532520
_space_group_name_H-M_alt            'P 3 2 1'
_space_group_IT_number               150

loop_
_space_group_symop_operation_xyz
   'x, y, z'
   '-y, x-y, z'
   '-x+y, -x, z'
   'y, x, -z'
   'x-y, -y, -z'
   '-x, -x+y, -z'

loop_
   _atom_site_label
   _atom_site_occupancy
   _atom_site_fract_x
   _atom_site_fract_y
   _atom_site_fract_z
   _atom_site_adp_type
   _atom_site_B_iso_or_equiv
   _atom_site_type_symbol
   Te1         1.0     0.0449707837385 -0.0734575499611 -0.4591386732667    Biso  1.000000 Te
   Te2         1.0     0.3048966582030 0.4760794584603 -0.4584924560112    Biso  1.000000 Te
   Te3         1.0     0.2675344422757 0.2807397970661 -0.4573097637000    Biso  1.000000 Te
   Te4         1.0     0.2341192325961 0.0859895363689 -0.4588753081742    Biso  1.000000 Te
   Te5         1.0     0.2054454361334 -0.1082744161443 -0.4589930013468    Biso  1.000000 Te
   Te6         1.0     0.1402791968342 -0.4950744880257 -0.4590337968763    Biso  1.000000 Te
   Te7         1.0     0.4636667423232 0.4388190287649 -0.4579231339969    Biso  1.000000 Te
   Te8         1.0     0.4281557673695 0.2442599335550 -0.4583767921084    Biso  1.000000 Te
   Te9         1.0     0.3982412414750 0.0543014505015 -0.4595606201489    Biso  1.000000 Te
   Te10        1.0     0.3333333333333 -0.3333333333333 -0.4594185209357    Biso  1.000000 Te
   Te11        1.0     -0.2162020732787 0.3773688749938 -0.4591903784452    Biso  1.000000 Te
   Te12        1.0     0.0441014847778 -0.0741452158246 -0.3697468701255    Biso  1.000000 Te
   Te13        1.0     0.3024971979323 0.4748775795787 -0.3699717604508    Biso  1.000000 Te
   Te14        1.0     0.2677410703412 0.2797954965940 -0.3703604961363    Biso  1.000000 Te
   Te15        1.0     0.2356807860412 0.0851429846604 -0.3702944722036    Biso  1.000000 Te
   Te16        1.0     0.2060286033868 -0.1068208539885 -0.3703481791356    Biso  1.000000 Te
   Te17        1.0     0.1394046450159 -0.4956802027696 -0.3702634044939    Biso  1.000000 Te
   Te18        1.0     0.4641255512059 0.4409201638026 -0.3703335820140    Biso  1.000000 Te
   Te19        1.0     0.4291710492745 0.2448920407400 -0.3701739882002    Biso  1.000000 Te
   Te20        1.0     0.3974858757017 0.0535052138301 -0.3703346366191    Biso  1.000000 Te
   Te21        1.0     0.3333333333333 -0.3333333333333 -0.3695360694212    Biso  1.000000 Te
   Te22        1.0     -0.2155584819150 0.3763617141679 -0.3698488165799    Biso  1.000000 Te
   W1          1.0     0.1172023939626 0.0438848209593 -0.4144214186364    Biso  1.000000 W
   W2          1.0     0.3777568372603 -0.4062933812415 -0.4143599722691    Biso  1.000000 W
   W3          1.0     0.3455012131569 0.3995405525701 -0.4140686188529    Biso  1.000000 W
   W4          1.0     0.3105262917601 0.2032678074969 -0.4143539152598    Biso  1.000000 W
   W5          1.0     0.2801107853849 0.0113225697862 -0.4151517762777    Biso  1.000000 W
   W6          1.0     0.2464394740630 -0.1845159766236 -0.4146940977268    Biso  1.000000 W
   W7          1.0     0.1476241447659 0.2360774743527 -0.4143743421806    Biso  1.000000 W
   W8          1.0     -0.4612232425554 -0.4399855443749 -0.4149205323874    Biso  1.000000 W
   W9          1.0     -0.4944103439030 0.3629037163514 -0.4142604312601    Biso  1.000000 W
   W10         1.0     0.4741459201413 0.1708199716160 -0.4147423087431    Biso  1.000000 W
   W11         1.0     0.3333333333333 -0.3333333333333 0.4143887995728    Biso  1.000000 W
```

53. WTe_2/WTe_2-60

```
_cell_length_a                        3.404210
_cell_length_b                        3.404210
_cell_length_c                        40.000000
_cell_angle_alpha                     90.000000
_cell_angle_beta                      90.000000
_cell_angle_gamma                     120.000000
_cell_volume                          401.442541
_space_group_name_H-M_alt             'P -3 m 1'
_space_group_IT_number                164

loop_
_space_group_symop_operation_xyz
   'x, y, z'
   '-x, -y, -z'
   '-y, x-y, z'
   'y, -x+y, -z'
   '-x+y, -x, z'
   'x-y, x, -z'
   'y, x, -z'
   '-y, -x, z'
   'x-y, -y, -z'
   '-x+y, y, z'
   '-x, -x+y, -z'
   'x, x-y, z'

loop_
   _atom_site_label
   _atom_site_occupancy
   _atom_site_fract_x
   _atom_site_fract_y
   _atom_site_fract_z
   _atom_site_adp_type
   _atom_site_B_iso_or_equiv
   _atom_site_type_symbol
   Te1        1.0     0.3333333333333 -0.3333333333333 0.4627892013537    Biso  1.000000 Te
   Te2        1.0     0.3333333333333 -0.3333333333333 0.3742019119843    Biso  1.000000 Te
   W1         1.0     0.3333333333333 -0.3333333333333 -0.4185452378607    Biso  1.000000 W
```

54. WTe_2/WTe_2-77.9

```
_cell_length_a                         18.953699
_cell_length_b                         18.953699
_cell_length_c                         40.000000
_cell_angle_alpha                      90.000000
_cell_angle_beta                       90.000000
_cell_angle_gamma                      120.000000
_cell_volume                           12444.532520
_space_group_name_H-M_alt              'P 3 2 1'
_space_group_IT_number                 150

loop_
_space_group_symop_operation_xyz
   'x, y, z'
   '-y, x-y, z'
   '-x+y, -x, z'
   'y, x, -z'
   'x-y, -y, -z'
   '-x, -x+y, -z'

loop_
   _atom_site_label
   _atom_site_occupancy
   _atom_site_fract_x
   _atom_site_fract_y
   _atom_site_fract_z
   _atom_site_adp_type
   _atom_site_B_iso_or_equiv
   _atom_site_type_symbol
   Te1         1.0    -0.0449736883855 0.0734525808516 0.4591314520114    Biso  1.000000 Te
   Te2         1.0    -0.2054492708652 0.1082603313419 0.4589870481404    Biso  1.000000 Te
   Te3         1.0    -0.3646458013924 0.1402815880790 0.4590296410212    Biso  1.000000 Te
   Te4         1.0    0.4760743922010 0.1711859120462 0.4584882892727    Biso  1.000000 Te
   Te5         1.0    0.1481268989582 0.2341218441803 0.4588675726105    Biso  1.000000 Te
   Te6         1.0    -0.0132066843097 0.2675273425748 0.4573063432305    Biso  1.000000 Te
   Te7         1.0    0.3333333333333 -0.3333333333333 -0.4594182166899    Biso  1.000000 Te
   Te8         1.0    0.3439379229752 0.3982413967108 0.4595543487082    Biso  1.000000 Te
   Te9         1.0    0.1838899267150 0.4281581224658 0.4583711474731    Biso  1.000000 Te
   Te10        1.0    0.0248426415646 0.4636644231209 0.4579199131525    Biso  1.000000 Te
   Te11        1.0    0.3773638184379 -0.4064313718305 0.4591870578474    Biso  1.000000 Te
   Te12        1.0    -0.0441072671031 0.0741413711051 0.3697388294512    Biso  1.000000 Te
   Te13        1.0    -0.2060341203641 0.1068141685789 0.3703431673138    Biso  1.000000 Te
   Te14        1.0    -0.3649175347611 0.1394083580535 0.3702610670458    Biso  1.000000 Te
   Te15        1.0    0.4748779126916 0.1723831252271 0.3699706399629    Biso  1.000000 Te
   Te16        1.0    0.1505338593552 0.2356848071456 0.3702873157783    Biso  1.000000 Te
   Te17        1.0    -0.0120613700914 0.2677366880117 0.3703561583039    Biso  1.000000 Te
   Te18        1.0    0.3333333333333 -0.3333333333333 -0.3695283615693    Biso  1.000000 Te
   Te19        1.0    0.3439827187592 0.3974936547575 0.3703304492492    Biso  1.000000 Te
   Te20        1.0    0.1842765957775 0.4291783646073 0.3701717589900    Biso  1.000000 Te
   Te21        1.0    0.0232032843676 0.4641235520600 0.3703323950597    Biso  1.000000 Te
   Te22        1.0    0.3763585995517 -0.4080803282266 0.3698424581750    Biso  1.000000 Te
   W1          1.0    0.0733124427409 0.1172030751995 0.4144134426629    Biso  1.000000 W
   W2          1.0    -0.0884577079570 0.1476167528093 0.4143689858155    Biso  1.000000 W
   W3          1.0    -0.2464412310777 0.1845081332443 0.4146903039091    Biso  1.000000 W
   W4          1.0    -0.4062895516880 0.2159610127334 0.4143575217660    Biso  1.000000 W
   W5          1.0    0.2687818093064 0.2801144392156 0.4151438636372    Biso  1.000000 W
   W6          1.0    0.1072525037834 0.3105267116438 0.4143480818283    Biso  1.000000 W
   W7          1.0    -0.0540419966398 0.3454964903485 0.4140662103791    Biso  1.000000 W
   W8          1.0    0.4612226069749 0.4399824220867 0.4149163757413    Biso  1.000000 W
   W9          1.0    0.3033252442408 0.4741510881127 0.4147379080185    Biso  1.000000 W
   W10         1.0    0.1426865800029 -0.4943984746469 0.4142576963185    Biso  1.000000 W
   W11         1.0    0.3333333333333 -0.3333333333333 0.4143840164428    Biso  1.000000 W
```

55. WTe_2/WTe_2-87.8

```
_cell_length_a                         12.273987
_cell_length_b                         12.273987
_cell_length_c                         40.000000
_cell_angle_alpha                      90.000000
_cell_angle_beta                       90.000000
_cell_angle_gamma                      120.000000
_cell_volume                           5218.695146
_space_group_name_H-M_alt              'P 3 2 1'
_space_group_IT_number                 150

loop_
_space_group_symop_operation_xyz
   'x, y, z'
   '-y, x-y, z'
   '-x+y, -x, z'
   'y, x, -z'
   'x-y, -y, -z'
   '-x, -x+y, -z'

loop_
   _atom_site_label
   _atom_site_occupancy
   _atom_site_fract_x
   _atom_site_fract_y
   _atom_site_fract_z
   _atom_site_adp_type
   _atom_site_B_iso_or_equiv
   _atom_site_type_symbol
   Te1        1.0    -0.0565238668607 -0.1812214864819 -0.4589627673277    Biso  1.000000 Te
   Te2        1.0    -0.2815100613079 -0.4903576601814 -0.4599530491570    Biso  1.000000 Te
   Te3        1.0    0.2553246778822 -0.1002643735453 -0.4601001564932    Biso  1.000000 Te
   Te4        1.0    0.0327828795081 -0.4074119883732 -0.4569852224368    Biso  1.000000 Te
   Te5        1.0    0.3333333333333 -0.3333333333333 -0.4609380959932    Biso  1.000000 Te
   Te6        1.0    -0.0548432167537 -0.1813181620738 -0.3707169426370    Biso  1.000000 Te
   Te7        1.0    -0.2813249249453 -0.4881537822388 -0.3699456323403    Biso  1.000000 Te
   Te8        1.0    0.2568796495320 -0.0998785861874 -0.3696713895775    Biso  1.000000 Te
   Te9        1.0    0.0300244770133 -0.4091775851914 -0.3726321889933    Biso  1.000000 Te
   Te10       1.0    0.3333333333333 -0.3333333333333 -0.3686687129073    Biso  1.000000 Te
   W1         1.0    0.1252910950035 -0.0563372022083 -0.4148497486167    Biso  1.000000 W
   W2         1.0    -0.1058417077697 -0.3606064851013 -0.4151442647957    Biso  1.000000 W
   W3         1.0    0.3333333333333 -0.3333333333333 0.4146838594705    Biso  1.000000 W
   W4         1.0    0.4310984423603 0.0287026850408 -0.4152164468186    Biso  1.000000 W
   W5         1.0    0.2190023577428 -0.2721459889704 -0.4148046764437    Biso  1.000000 W
```

56. WTe_2/WTe_2-92.2

```
_cell_length_a                        12.273978
_cell_length_b                        12.273978
_cell_length_c                        40.000000
_cell_angle_alpha                     90.000000
_cell_angle_beta                      90.000000
_cell_angle_gamma                     120.000000
_cell_volume                          5218.687847
_space_group_name_H-M_alt             'P 3 1 2'
_space_group_IT_number                149

loop_
_space_group_symop_operation_xyz
   'x, y, z'
   '-y, x-y, z'
   '-x+y, -x, z'
   '-y, -x, -z'
   '-x+y, y, -z'
   'x, x-y, -z'

loop_
   _atom_site_label
   _atom_site_occupancy
   _atom_site_fract_x
   _atom_site_fract_y
   _atom_site_fract_z
   _atom_site_adp_type
   _atom_site_B_iso_or_equiv
   _atom_site_type_symbol
   Te1        1.0    0.1340000105444 0.1793272035157 0.4583726805101    Biso  1.000000 Te
   Te2        1.0    -0.0239488570708 -0.4359865311379 0.4603661998144    Biso  1.000000 Te
   Te3        1.0    -0.1017430604486 0.2558211318434 0.4602082008712    Biso  1.000000 Te
   Te4        1.0    -0.3333333333333 0.3333333333333 0.4557041114076    Biso  1.000000 Te
   Te5        1.0    -0.4861109661925 -0.2878267745188 0.4584275378255    Biso  1.000000 Te
   Te6        1.0    0.1327828161436 0.1800221511274 0.3711721904592    Biso  1.000000 Te
   Te7        1.0    -0.0245063420055 -0.4348796230313 0.3692881052625    Biso  1.000000 Te
   Te8        1.0    -0.1023988170081 0.2556165327608 0.3692525019392    Biso  1.000000 Te
   Te9        1.0    -0.3333333333333 0.3333333333333 0.3734177139836    Biso  1.000000 Te
   Te10       1.0    -0.4871853071894 -0.2856070947299 0.3712621722606    Biso  1.000000 Te
   W1         1.0    0.2606418134863 0.3631363715674 0.4150437818599    Biso  1.000000 W
   W2         1.0    0.1810664167215 0.0460184004373 0.4148325861741    Biso  1.000000 W
   W3         1.0    0.0165214132981 0.4328614327172 0.4146201603152    Biso  1.000000 W
   W4         1.0    -0.1961565716586 -0.4753990281598 0.4149216945669    Biso  1.000000 W
   W5         1.0    0.3333333333333 -0.3333333333333 0.4152633440713    Biso  1.000000 W
```

57. WTe_2/WTe_2-102.1

```
_cell_length_a                    18.953712
_cell_length_b                    18.953712
_cell_length_c                    40.000000
_cell_angle_alpha                 90.000000
_cell_angle_beta                  90.000000
_cell_angle_gamma                 120.000000
_cell_volume                      12444.550053
_space_group_name_H-M_alt         'P 3 1 2'
_space_group_IT_number            149

loop_
_space_group_symop_operation_xyz
   'x, y, z'
   '-y, x-y, z'
   '-x+y, -x, z'
   '-y, -x, -z'
   '-x+y, y, -z'
   'x, x-y, -z'

loop_
   _atom_site_label
   _atom_site_occupancy
   _atom_site_fract_x
   _atom_site_fract_y
   _atom_site_fract_z
   _atom_site_adp_type
   _atom_site_B_iso_or_equiv
   _atom_site_type_symbol
   Te1        1.0    0.2974976749470 0.4714276132114 0.4588702789039    Biso  1.000000 Te
   Te2        1.0    0.2693710461127 0.2789330637127 0.4599324046533    Biso  1.000000 Te
   Te3        1.0    0.4934419290700 -0.3689812335748 0.4586593557334    Biso  1.000000 Te
   Te4        1.0    0.4629298778173 0.4418832038485 0.4593987337554    Biso  1.000000 Te
   Te5        1.0    -0.2149858230494 0.3729147030519 0.4578752129155    Biso  1.000000 Te
   Te6        1.0    -0.2493182221757 0.1806235137600 0.4589918787458    Biso  1.000000 Te
   Te7        1.0    -0.3127142218681 -0.2046764156519 0.4590190123972    Biso  1.000000 Te
   Te8        1.0    -0.3427501723232 -0.3967971988848 0.4592082385702    Biso  1.000000 Te
   Te9        1.0    0.0399962499677 -0.0783526806821 0.4578421893300    Biso  1.000000 Te
   Te10       1.0    -0.0832664577233 0.1532173189936 0.4588856455832    Biso  1.000000 Te
   Te11       1.0    0.3333333333333 -0.3333333333333 0.4569633346592    Biso  1.000000 Te
   Te12       1.0    0.2994593663298 0.4711069538311 0.3703541954270    Biso  1.000000 Te
   Te13       1.0    0.2697552341549 0.2798807883937 0.3702737022556    Biso  1.000000 Te
   Te14       1.0    0.4941834974467 -0.3675362284696 0.3700182359763    Biso  1.000000 Te
   Te15       1.0    0.4616419134463 0.4405802339113 0.3704458694426    Biso  1.000000 Te
   Te16       1.0    -0.2143543582373 0.3751461600438 0.3704224562220    Biso  1.000000 Te
   Te17       1.0    -0.2487077177908 0.1805758713559 0.3703554888045    Biso  1.000000 Te
   Te18       1.0    -0.3121430338052 -0.2039075510532 0.3701160164231    Biso  1.000000 Te
   Te19       1.0    -0.3435926379242 -0.3973659341904 0.3697181780787    Biso  1.000000 Te
   Te20       1.0    0.0414865574306 -0.0775589549606 0.3704412801058    Biso  1.000000 Te
   Te21       1.0    -0.0855742302307 0.1516557855649 0.3701613103491    Biso  1.000000 Te
   Te22       1.0    0.3333333333333 -0.3333333333333 0.3707190000316    Biso  1.000000 Te
   W1         1.0    0.3758083605708 -0.4112571408507 0.4143697658481    Biso  1.000000 W
   W2         1.0    0.3435000492204 0.3968975555156 0.4152679402950    Biso  1.000000 W
   W3         1.0    -0.4306332715741 -0.2496643984645 0.4145001537197    Biso  1.000000 W
   W4         1.0    -0.4612115241192 -0.4415603514365 0.4145492397282    Biso  1.000000 W
   W5         1.0    -0.1388553683159 0.4941148005126 0.4148277941608    Biso  1.000000 W
   W6         1.0    -0.1715690150035 0.2981350122216 0.4143735369118    Biso  1.000000 W
   W7         1.0    -0.2039001861754 0.1069375691773 0.4150021851849    Biso  1.000000 W
   W8         1.0    -0.2385706078994 -0.0855142037368 0.4147940809280    Biso  1.000000 W
   W9         1.0    -0.2700831665862 -0.2797148214746 0.4143925486705    Biso  1.000000 W
   W10        1.0    0.1189993384534 0.0410073208986 0.4143307290910    Biso  1.000000 W
   W11        1.0    -0.3333333333333 0.3333333333333 0.4142451849613    Biso  1.000000 W
```

58. WTe_2/WTe_2-120

```
_cell_length_a                        3.404187
_cell_length_b                        3.404187
_cell_length_c                        40.000000
_cell_angle_alpha                     90.000000
_cell_angle_beta                      90.000000
_cell_angle_gamma                     120.000000
_cell_volume                          401.437143
_space_group_name_H-M_alt             'P -6 m 2'
_space_group_IT_number                187

loop_
_space_group_symop_operation_xyz
   'x, y, z'
   '-y, x-y, z'
   '-x+y, -x, z'
   'x, y, -z'
   '-y, x-y, -z'
   '-x+y, -x, -z'
   '-y, -x, z'
   '-x+y, y, z'
   'x, x-y, z'
   '-y, -x, -z'
   '-x+y, y, -z'
   'x, x-y, -z'

loop_
   _atom_site_label
   _atom_site_occupancy
   _atom_site_fract_x
   _atom_site_fract_y
   _atom_site_fract_z
   _atom_site_adp_type
   _atom_site_B_iso_or_equiv
   _atom_site_type_symbol
   Te1        1.0     0.3333333333333 -0.3333333333333 -0.4531482427751    Biso  1.000000 Te
   Te2        1.0     0.3333333333333 -0.3333333333333 -0.3645150561348    Biso  1.000000 Te
   W1         1.0     0.0000000000000 0.0000000000000 -0.4088830586302    Biso  1.000000 W
```

59. 1T-MoS_2/$MoSe_2$-0

```
_cell_length_a                         3.162258
_cell_length_b                         3.161922
_cell_length_c                         40.000000
_cell_angle_alpha                      90.000000
_cell_angle_beta                       90.000000
_cell_angle_gamma                      119.994164
_cell_volume                           346.389404
_space_group_name_H-M_alt              'P 1'
_space_group_IT_number                 1

loop_
_space_group_symop_operation_xyz
   'x, y, z'

loop_
   _atom_site_label
   _atom_site_occupancy
   _atom_site_fract_x
   _atom_site_fract_y
   _atom_site_fract_z
   _atom_site_adp_type
   _atom_site_B_iso_or_equiv
   _atom_site_type_symbol
   MO001       1.0     0.013488    -0.484844     0.296439    Biso  1.000000 Mo
   S002        1.0    -0.319870    -0.151432     0.334799    Biso  1.000000 S
   S003        1.0     0.346998     0.181893     0.257888    Biso  1.000000 S
   MO004       1.0    -0.320591    -0.152023     0.438708    Biso  1.000000 Mo
   SE005       1.0     0.346023     0.181285     0.480236    Biso  1.000000 Se
   SE006       1.0     0.346052     0.181320     0.397490    Biso  1.000000 Se
```

60. 1T-MoS_2/$MoSe_2$-60

```
_cell_length_a                        3.152516
_cell_length_b                        3.152291
_cell_length_c                        40.000000
_cell_angle_alpha                     90.000000
_cell_angle_beta                      90.000000
_cell_angle_gamma                     60.004658
_cell_volume                          344.266369
_space_group_name_H-M_alt             'P 1'
_space_group_IT_number                1

loop_
_space_group_symop_operation_xyz
   'x, y, z'

loop_
   _atom_site_label
   _atom_site_occupancy
   _atom_site_fract_x
   _atom_site_fract_y
   _atom_site_fract_z
   _atom_site_adp_type
   _atom_site_B_iso_or_equiv
   _atom_site_type_symbol
   MO001       1.0    -0.181044     0.346259     0.294644    Biso  1.000000 Mo
   S002        1.0     0.152304    -0.320479     0.333197    Biso  1.000000 S
   S003        1.0     0.485489     0.012845     0.256001    Biso  1.000000 S
   MO004       1.0     0.151526    -0.320025     0.440488    Biso  1.000000 Mo
   SE005       1.0    -0.181840     0.346669     0.482117    Biso  1.000000 Se
   SE006       1.0    -0.181856     0.346630     0.399113    Biso  1.000000 Se
```

61. 1H-MoS_2/$MoSe_2$-0

```
_cell_length_a                        3.148814
_cell_length_b                        3.148233
_cell_length_c                        40.000000
_cell_angle_alpha                     90.000000
_cell_angle_beta                      90.000000
_cell_angle_gamma                     119.996750
_cell_volume                          343.414561
_space_group_name_H-M_alt             'P 1'
_space_group_IT_number                1

loop_
_space_group_symop_operation_xyz
   'x, y, z'

loop_
   _atom_site_label
   _atom_site_occupancy
   _atom_site_fract_x
   _atom_site_fract_y
   _atom_site_fract_z
   _atom_site_adp_type
   _atom_site_B_iso_or_equiv
   _atom_site_type_symbol
   MO001      1.0    0.347203   -0.317841    0.288688   Biso  1.000000 Mo
   S002       1.0   -0.319439    0.348847    0.250397   Biso  1.000000 S
   S003       1.0   -0.319431    0.348787    0.326715   Biso  1.000000 S
   MO004      1.0    0.347319   -0.316869    0.432459   Biso  1.000000 Mo
   SE005      1.0    0.013989    0.016454    0.474201   Biso  1.000000 Se
   SE006      1.0    0.013989    0.016483    0.391180   Biso  1.000000 Se
```

62. 1H-MoS_2/$MoSe_2$-60

```
_cell_length_a                          3.143259
_cell_length_b                          3.142901
_cell_length_c                          40.000000
_cell_angle_alpha                       90.000000
_cell_angle_beta                        90.000000
_cell_angle_gamma                       60.000793
_cell_volume                            342.219666
_space_group_name_H-M_alt               'P 1'
_space_group_IT_number                  1

loop_
_space_group_symop_operation_xyz
   'x, y, z'

loop_
   _atom_site_label
   _atom_site_occupancy
   _atom_site_fract_x
   _atom_site_fract_y
   _atom_site_fract_z
   _atom_site_adp_type
   _atom_site_B_iso_or_equiv
   _atom_site_type_symbol
   MO001       1.0    -0.349391    -0.319408     0.289778    Biso  1.000000 Mo
   S002        1.0     0.317251     0.347286     0.251460    Biso  1.000000 S
   S003        1.0     0.317225     0.347262     0.327898    Biso  1.000000 S
   MO004       1.0     0.317420     0.347408     0.433909    Biso  1.000000 Mo
   SE005       1.0    -0.015937     0.014090     0.475611    Biso  1.000000 Se
   SE006       1.0    -0.015946     0.014132     0.392483    Biso  1.000000 Se
```

63. 1T-MoS_2/$MoTe_2$-0

```
_cell_length_a                        3.296017
_cell_length_b                        3.296076
_cell_length_c                        40.000000
_cell_angle_alpha                     90.000000
_cell_angle_beta                      90.000000
_cell_angle_gamma                     119.979340
_cell_volume                          376.415639
_space_group_name_H-M_alt             'P 1'
_space_group_IT_number                1

loop_
_space_group_symop_operation_xyz
   'x, y, z'

loop_
   _atom_site_label
   _atom_site_occupancy
   _atom_site_fract_x
   _atom_site_fract_y
   _atom_site_fract_z
   _atom_site_adp_type
   _atom_site_B_iso_or_equiv
   _atom_site_type_symbol
   MO001       1.0     0.362936    -0.309565     0.302646    Biso  1.000000 Mo
   S002        1.0     0.029515     0.023860     0.339270    Biso  1.000000 S
   S003        1.0    -0.303841     0.357063     0.265693    Biso  1.000000 S
   TE004       1.0    -0.303883     0.356642     0.402992    Biso  1.000000 Te
   TE005       1.0    -0.303883     0.356662     0.493341    Biso  1.000000 Te
   MO006       1.0     0.362615    -0.309912     0.447778    Biso  1.000000 Mo
```

64. 1T-MoS_2/$MoTe_2$-60

```
_cell_length_a                      3.285804
_cell_length_b                      3.285928
_cell_length_c                      40.000000
_cell_angle_alpha                   90.000000
_cell_angle_beta                    90.000000
_cell_angle_gamma                   59.999580
_cell_volume                        374.014543
_space_group_name_H-M_alt           'P 1'
_space_group_IT_number              1

loop_
_space_group_symop_operation_xyz
   'x, y, z'

loop_
   _atom_site_label
   _atom_site_occupancy
   _atom_site_fract_x
   _atom_site_fract_y
   _atom_site_fract_z
   _atom_site_adp_type
   _atom_site_B_iso_or_equiv
   _atom_site_type_symbol
   MO001      1.0    -0.357231    -0.303402     0.301111    Biso  1.000000 Mo
   S002       1.0    -0.023985     0.029730     0.337834    Biso  1.000000 S
   S003       1.0     0.309441     0.363150     0.264019    Biso  1.000000 S
   TE004      1.0    -0.356379    -0.304245     0.404308    Biso  1.000000 Te
   TE005      1.0    -0.356411    -0.304204     0.495036    Biso  1.000000 Te
   MO006      1.0     0.310266     0.362451     0.449412    Biso  1.000000 Mo
```

65. 1H-MoS_2/$MoTe_2$-0

```
_cell_length_a                      3.237016
_cell_length_b                      3.236898
_cell_length_c                      40.000000
_cell_angle_alpha                   90.000000
_cell_angle_beta                    90.000000
_cell_angle_gamma                   119.998940
_cell_volume                        362.968646
_space_group_name_H-M_alt           'P 1'
_space_group_IT_number              1

loop_
_space_group_symop_operation_xyz
   'x, y, z'

loop_
   _atom_site_label
   _atom_site_occupancy
   _atom_site_fract_x
   _atom_site_fract_y
   _atom_site_fract_z
   _atom_site_adp_type
   _atom_site_B_iso_or_equiv
   _atom_site_type_symbol
   MO001      1.0    0.029688   -0.476081    0.286967   Biso  1.000000 Mo
   S002       1.0    0.363037    0.190592    0.249521   Biso  1.000000 S
   S003       1.0    0.363049    0.190593    0.324414   Biso  1.000000 S
   TE004      1.0    0.030552   -0.475657    0.393783   Biso  1.000000 Te
   TE005      1.0    0.030546   -0.475655    0.485645   Biso  1.000000 Te
   MO006      1.0   -0.302801   -0.142333    0.439489   Biso  1.000000 Mo
```

66. 1H-MoS_2/$MoTe_2$-60

```
_cell_length_a                          3.231866
_cell_length_b                          3.231739
_cell_length_c                          40.000000
_cell_angle_alpha                       90.000000
_cell_angle_beta                        90.000000
_cell_angle_gamma                       59.995693
_cell_volume                            361.794024
_space_group_name_H-M_alt               'P 1'
_space_group_IT_number                  1

loop_
_space_group_symop_operation_xyz
   'x, y, z'

loop_
   _atom_site_label
   _atom_site_occupancy
   _atom_site_fract_x
   _atom_site_fract_y
   _atom_site_fract_z
   _atom_site_adp_type
   _atom_site_B_iso_or_equiv
   _atom_site_type_symbol
   MO001       1.0    -0.357223    -0.303286     0.288527    Biso  1.000000 Mo
   S002        1.0     0.309425     0.363393     0.251036    Biso  1.000000 S
   S003        1.0     0.309414     0.363398     0.326047    Biso  1.000000 S
   TE004       1.0    -0.357756    -0.303032     0.394559    Biso  1.000000 Te
   TE005       1.0    -0.357750    -0.303042     0.486640    Biso  1.000000 Te
   MO006       1.0     0.308909     0.363618     0.440511    Biso  1.000000 Mo
```

67. 1T-MoS_2/WS_2-0

```
_cell_length_a                         3.107949
_cell_length_b                         14.387214
_cell_length_c                         40.000000
_cell_angle_alpha                      90.000000
_cell_angle_beta                       90.000000
_cell_angle_gamma                      49.605309
_cell_volume                           1362.186490
_space_group_name_H-M_alt              'P 1'
_space_group_IT_number                 1

loop_
_space_group_symop_operation_xyz
   'x, y, z'

loop_
   _atom_site_label
   _atom_site_occupancy
   _atom_site_fract_x
   _atom_site_fract_y
   _atom_site_fract_z
   _atom_site_adp_type
   _atom_site_B_iso_or_equiv
   _atom_site_type_symbol
   S001       1.0     0.248996    -0.249742     0.344857    Biso  1.000000 S
   S002       1.0    -0.492630     0.497459     0.336029    Biso  1.000000 S
   S003       1.0    -0.250317     0.250213     0.344861    Biso  1.000000 S
   S004       1.0     0.006440    -0.002523     0.336011    Biso  1.000000 S
   S005       1.0    -0.253724    -0.082220     0.268609    Biso  1.000000 S
   S006       1.0     0.004610    -0.334979     0.259369    Biso  1.000000 S
   S007       1.0     0.247008     0.417799     0.268619    Biso  1.000000 S
   S008       1.0    -0.495803     0.165053     0.259362    Biso  1.000000 S
   S009       1.0    -0.497235    -0.167321     0.481854    Biso  1.000000 S
   S010       1.0    -0.252592    -0.415594     0.481514    Biso  1.000000 S
   S011       1.0     0.002755     0.332706     0.481830    Biso  1.000000 S
   S012       1.0     0.247241     0.084460     0.481519    Biso  1.000000 S
   S013       1.0     0.495007    -0.164668     0.405096    Biso  1.000000 S
   S014       1.0    -0.244663    -0.418280     0.404578    Biso  1.000000 S
   S015       1.0    -0.004782     0.335294     0.405070    Biso  1.000000 S
   S016       1.0     0.255701     0.081632     0.404584    Biso  1.000000 S
   MO017      1.0    -0.421348    -0.192929     0.304120    Biso  1.000000 Mo
   MO018      1.0    -0.325460    -0.391742     0.300591    Biso  1.000000 Mo
   MO019      1.0     0.078591     0.307070     0.304123    Biso  1.000000 Mo
   MO020      1.0     0.174910     0.108267     0.300581    Biso  1.000000 Mo
   W021       1.0    -0.251275    -0.082634     0.443381    Biso  1.000000 W
   W022       1.0    -0.001097    -0.332761     0.442690    Biso  1.000000 W
   W023       1.0     0.248789     0.417369     0.443355    Biso  1.000000 W
   W024       1.0     0.498921     0.167235     0.442682    Biso  1.000000 W
```

68. 1T-MoS_2/WS_2-60

```
_cell_length_a                         3.121995
_cell_length_b                         3.121687
_cell_length_c                         40.000000
_cell_angle_alpha                      90.000000
_cell_angle_beta                       90.000000
_cell_angle_gamma                      60.003979
_cell_volume                           337.621099
_space_group_name_H-M_alt              'P 1'
_space_group_IT_number                 1

loop_
_space_group_symop_operation_xyz
   'x, y, z'

loop_
   _atom_site_label
   _atom_site_occupancy
   _atom_site_fract_x
   _atom_site_fract_y
   _atom_site_fract_z
   _atom_site_adp_type
   _atom_site_B_iso_or_equiv
   _atom_site_type_symbol
   MO001       1.0    -0.334301    -0.332131     0.297549    Biso  1.000000 Mo
   S002        1.0    -0.000683     0.000425     0.336320    Biso  1.000000 S
   S003        1.0     0.332361     0.333801     0.258356    Biso  1.000000 S
   W004        1.0    -0.333671    -0.332903     0.435373    Biso  1.000000 W
   S005        1.0     0.332978     0.333752     0.474173    Biso  1.000000 S
   S006        1.0     0.332965     0.333716     0.397048    Biso  1.000000 S
```

69. 1H-MoS_2/WS_2-0

```
_cell_length_a                         3.098935
_cell_length_b                         3.098944
_cell_length_c                         40.000000
_cell_angle_alpha                      90.000000
_cell_angle_beta                       90.000000
_cell_angle_gamma                      119.995552
_cell_volume                           332.687328
_space_group_name_H-M_alt              'P 1'
_space_group_IT_number                 1

loop_
_space_group_symop_operation_xyz
   'x, y, z'

loop_
   _atom_site_label
   _atom_site_occupancy
   _atom_site_fract_x
   _atom_site_fract_y
   _atom_site_fract_z
   _atom_site_adp_type
   _atom_site_B_iso_or_equiv
   _atom_site_type_symbol
   MO001       1.0     0.334066    -0.333010     0.290710    Biso  1.000000 Mo
   S002        1.0    -0.332586     0.333647     0.251982    Biso  1.000000 S
   S003        1.0    -0.332582     0.333622     0.329300    Biso  1.000000 S
   W004        1.0    -0.332021     0.335666     0.433260    Biso  1.000000 W
   S005        1.0     0.334627    -0.330990     0.472121    Biso  1.000000 S
   S006        1.0     0.334626    -0.330965     0.394547    Biso  1.000000 S
```

70. 1H-MoS_2/WS_2-60

```
_cell_length_a                       3.090787
_cell_length_b                       3.090559
_cell_length_c                       40.000000
_cell_angle_alpha                    90.000000
_cell_angle_beta                     90.000000
_cell_angle_gamma                    60.001328
_cell_volume                         330.904398
_space_group_name_H-M_alt            'P 1'
_space_group_IT_number               1

loop_
_space_group_symop_operation_xyz
   'x, y, z'

loop_
   _atom_site_label
   _atom_site_occupancy
   _atom_site_fract_x
   _atom_site_fract_y
   _atom_site_fract_z
   _atom_site_adp_type
   _atom_site_B_iso_or_equiv
   _atom_site_type_symbol
   MO001      1.0    -0.331283    -0.333801     0.279426    Biso  1.000000 Mo
   S002       1.0     0.335303     0.332909     0.240658    Biso  1.000000 S
   S003       1.0     0.335371     0.332863     0.318026    Biso  1.000000 S
   W004       1.0    -0.338064    -0.330798     0.437030    Biso  1.000000 W
   S005       1.0     0.328623     0.335902     0.475997    Biso  1.000000 S
   S006       1.0     0.328641     0.335995     0.398283    Biso  1.000000 S
```

71. 1T-MoS_2/WSe_2-0

```
_cell_length_a                         15.915093
_cell_length_b                         3.176833
_cell_length_c                         40.000000
_cell_angle_alpha                      90.000000
_cell_angle_beta                       90.000000
_cell_angle_gamma                      119.931282
_cell_volume                           1752.647182
_space_group_name_H-M_alt              'P 1'
_space_group_IT_number                 1

loop_
_space_group_symop_operation_xyz
   'x, y, z'

loop_
   _atom_site_label
   _atom_site_occupancy
   _atom_site_fract_x
   _atom_site_fract_y
   _atom_site_fract_z
   _atom_site_adp_type
   _atom_site_B_iso_or_equiv
   _atom_site_type_symbol
   MO001       1.0     0.092824    -0.261782     0.300116    Biso  1.000000 Mo
   MO002       1.0     0.266678    -0.327555     0.300598    Biso  1.000000 Mo
   MO003       1.0     0.440239    -0.393528     0.301986    Biso  1.000000 Mo
   MO004       1.0    -0.312468    -0.275445     0.299897    Biso  1.000000 Mo
   MO005       1.0    -0.154074    -0.379387     0.302845    Biso  1.000000 Mo
   S006        1.0     0.205332     0.019074     0.341405    Biso  1.000000 S
   S007        1.0     0.395780    -0.004705     0.342073    Biso  1.000000 S
   S008        1.0    -0.403330    -0.002519     0.333719    Biso  1.000000 S
   S009        1.0    -0.197467     0.012240     0.343361    Biso  1.000000 S
   S010        1.0     0.003026     0.013629     0.333861    Biso  1.000000 S
   S011        1.0     0.136565     0.347423     0.259524    Biso  1.000000 S
   S012        1.0     0.328993     0.328243     0.259986    Biso  1.000000 S
   S013        1.0    -0.469283     0.332523     0.268716    Biso  1.000000 S
   S014        1.0    -0.269022     0.333219     0.258972    Biso  1.000000 S
   S015        1.0    -0.063800     0.346390     0.269024    Biso  1.000000 S
   W016        1.0     0.136040     0.346052     0.447040    Biso  1.000000 W
   W017        1.0     0.336836     0.348020     0.447312    Biso  1.000000 W
   W018        1.0    -0.463555     0.346951     0.446981    Biso  1.000000 W
   W019        1.0    -0.263543     0.346963     0.446178    Biso  1.000000 W
   W020        1.0    -0.063143     0.348048     0.446886    Biso  1.000000 W
   SE021       1.0     0.069900    -0.319268     0.405240    Biso  1.000000 Se
   SE022       1.0     0.269759    -0.319652     0.406282    Biso  1.000000 Se
   SE023       1.0     0.469807    -0.319639     0.405693    Biso  1.000000 Se
   SE024       1.0    -0.331822    -0.323788     0.404943    Biso  1.000000 Se
   SE025       1.0    -0.128592    -0.315555     0.405571    Biso  1.000000 Se
   SE026       1.0     0.069405    -0.320557     0.488885    Biso  1.000000 Se
   SE027       1.0     0.269396    -0.320586     0.489055    Biso  1.000000 Se
   SE028       1.0     0.470362    -0.318250     0.489012    Biso  1.000000 Se
   SE029       1.0    -0.329379    -0.317646     0.488234    Biso  1.000000 Se
   SE030       1.0    -0.130916    -0.321413     0.488502    Biso  1.000000 Se
```

72. 1T-MoS_2/WSe_2-60

```
_cell_length_a                         3.177441
_cell_length_b                         3.177231
_cell_length_c                         40.000000
_cell_angle_alpha                      90.000000
_cell_angle_beta                       90.000000
_cell_angle_gamma                      60.003315
_cell_volume                           349.728809
_space_group_name_H-M_alt              'P 1'
_space_group_IT_number                 1

loop_
_space_group_symop_operation_xyz
   'x, y, z'

loop_
   _atom_site_label
   _atom_site_occupancy
   _atom_site_fract_x
   _atom_site_fract_y
   _atom_site_fract_z
   _atom_site_adp_type
   _atom_site_B_iso_or_equiv
   _atom_site_type_symbol
   MO001       1.0    -0.343173    -0.325456     0.297260    Biso  1.000000 Mo
   S002        1.0    -0.009941     0.007855     0.335253    Biso  1.000000 S
   S003        1.0     0.323236     0.341176     0.258825    Biso  1.000000 S
   W004        1.0    -0.342821    -0.325519     0.438282    Biso  1.000000 W
   SE005       1.0     0.323834     0.341157     0.397029    Biso  1.000000 Se
   SE006       1.0     0.323826     0.341176     0.480232    Biso  1.000000 Se
```

73. 1H-MoS_2/WSe_2-0

```
_cell_length_a                        3.143054
_cell_length_b                        3.142512
_cell_length_c                        40.000000
_cell_angle_alpha                     90.000000
_cell_angle_beta                      90.000000
_cell_angle_gamma                     119.997887
_cell_volume                          342.159554
_space_group_name_H-M_alt             'P 1'
_space_group_IT_number                1

loop_
_space_group_symop_operation_xyz
   'x, y, z'

loop_
   _atom_site_label
   _atom_site_occupancy
   _atom_site_fract_x
   _atom_site_fract_y
   _atom_site_fract_z
   _atom_site_adp_type
   _atom_site_B_iso_or_equiv
   _atom_site_type_symbol
   MO001       1.0     0.342425    -0.324133     0.290966    Biso  1.000000 Mo
   S002        1.0    -0.324184     0.342527     0.252708    Biso  1.000000 S
   S003        1.0    -0.324081     0.342571     0.329194    Biso  1.000000 S
   W004        1.0    -0.325800     0.345088     0.438106    Biso  1.000000 W
   SE005       1.0     0.340803    -0.321582     0.396245    Biso  1.000000 Se
   SE006       1.0     0.340777    -0.321672     0.480240    Biso  1.000000 Se
```

74. 1H-MoS_2/WSe_2-60

```
_cell_length_a                        3.147087
_cell_length_b                        3.146920
_cell_length_c                        40.000000
_cell_angle_alpha                     90.000000
_cell_angle_beta                      90.000000
_cell_angle_gamma                     60.001877
_cell_volume                          343.078337
_space_group_name_H-M_alt             'P 1'
_space_group_IT_number                1

loop_
_space_group_symop_operation_xyz
   'x, y, z'

loop_
   _atom_site_label
   _atom_site_occupancy
   _atom_site_fract_x
   _atom_site_fract_y
   _atom_site_fract_z
   _atom_site_adp_type
   _atom_site_B_iso_or_equiv
   _atom_site_type_symbol
   MO001       1.0    -0.010621    -0.491537     0.287596    Biso  1.000000 Mo
   S002        1.0    -0.343972     0.175137     0.249379    Biso  1.000000 S
   S003        1.0    -0.343989     0.175144     0.325772    Biso  1.000000 S
   W004        1.0     0.323019    -0.158403     0.433924    Biso  1.000000 W
   SE005       1.0    -0.010334    -0.491724     0.392199    Biso  1.000000 Se
   SE006       1.0    -0.010333    -0.491728     0.476090    Biso  1.000000 Se
```

75. 1T-MoS_2/WTe_2-0

```
_cell_length_a                       3.272063
_cell_length_b                       16.623808
_cell_length_c                       40.000000
_cell_angle_alpha                    90.000000
_cell_angle_beta                     90.000000
_cell_angle_gamma                    119.472923
_cell_volume                         1894.196336
_space_group_name_H-M_alt            'P 1'
_space_group_IT_number               1

loop_
_space_group_symop_operation_xyz
   'x, y, z'

loop_
   _atom_site_label
   _atom_site_occupancy
   _atom_site_fract_x
   _atom_site_fract_y
   _atom_site_fract_z
   _atom_site_adp_type
   _atom_site_B_iso_or_equiv
   _atom_site_type_symbol
   MO001       1.0     0.291677     0.113089     0.301440    Biso  1.000000 Mo
   MO002       1.0     0.417511     0.363572     0.294867    Biso  1.000000 Mo
   MO003       1.0     0.424309    -0.433619     0.300828    Biso  1.000000 Mo
   MO004       1.0     0.308083    -0.280282     0.299835    Biso  1.000000 Mo
   MO005       1.0     0.407594    -0.040468     0.303975    Biso  1.000000 Mo
   S006        1.0     0.017487     0.203418     0.333223    Biso  1.000000 S
   S007        1.0     0.040989     0.412968     0.333839    Biso  1.000000 S
   S008        1.0     0.024441    -0.393573     0.340934    Biso  1.000000 S
   S009        1.0     0.023721    -0.194003     0.333668    Biso  1.000000 S
   S010        1.0     0.011675     0.001154     0.343636    Biso  1.000000 S
   S011        1.0    -0.314817     0.070509     0.261174    Biso  1.000000 S
   S012        1.0    -0.327139     0.265673     0.271052    Biso  1.000000 S
   S013        1.0    -0.261040     0.492187     0.262115    Biso  1.000000 S
   S014        1.0    -0.284309    -0.317240     0.259365    Biso  1.000000 S
   S015        1.0    -0.307128    -0.126358     0.270858    Biso  1.000000 S
   TE016       1.0     0.353459     0.138089     0.403837    Biso  1.000000 Te
   TE017       1.0     0.351543     0.337321     0.404041    Biso  1.000000 Te
   TE018       1.0     0.345432    -0.465135     0.405961    Biso  1.000000 Te
   TE019       1.0     0.347258    -0.264441     0.405039    Biso  1.000000 Te
   TE020       1.0     0.341232    -0.066855     0.405760    Biso  1.000000 Te
   TE021       1.0     0.348347     0.136038     0.494745    Biso  1.000000 Te
   TE022       1.0     0.343747     0.334192     0.495803    Biso  1.000000 Te
   TE023       1.0     0.347173    -0.464438     0.496302    Biso  1.000000 Te
   TE024       1.0     0.347548    -0.264327     0.496531    Biso  1.000000 Te
   TE025       1.0     0.352265    -0.062432     0.495676    Biso  1.000000 Te
   W026        1.0    -0.319148     0.069038     0.448959    Biso  1.000000 W
   W027        1.0    -0.318567     0.269262     0.449474    Biso  1.000000 W
   W028        1.0    -0.324040     0.467083     0.450782    Biso  1.000000 W
   W029        1.0    -0.320329    -0.331477     0.450638    Biso  1.000000 W
   W030        1.0    -0.323621    -0.132795     0.450642    Biso  1.000000 W
```

76. 1T-MoS_2/WTe_2-60

```
_cell_length_a                         3.293599
_cell_length_b                         3.293494
_cell_length_c                         40.000000
_cell_angle_alpha                      90.000000
_cell_angle_beta                       90.000000
_cell_angle_gamma                      59.991394
_cell_volume                           375.734036
_space_group_name_H-M_alt              'P 1'
_space_group_IT_number                 1

loop_
_space_group_symop_operation_xyz
   'x, y, z'

loop_
   _atom_site_label
   _atom_site_occupancy
   _atom_site_fract_x
   _atom_site_fract_y
   _atom_site_fract_z
   _atom_site_adp_type
   _atom_site_B_iso_or_equiv
   _atom_site_type_symbol
   MO001       1.0    -0.361484    -0.310591     0.302573    Biso  1.000000 Mo
   S002        1.0    -0.028224     0.022672     0.339264    Biso  1.000000 S
   S003        1.0     0.305159     0.356104     0.265554    Biso  1.000000 S
   TE004       1.0     0.305216     0.355797     0.403195    Biso  1.000000 Te
   TE005       1.0     0.305192     0.355804     0.494140    Biso  1.000000 Te
   W006        1.0    -0.361498    -0.310877     0.448274    Biso  1.000000 W
```

77. 1H-MoS_2/WTe_2-0

```
_cell_length_a                      3.231537
_cell_length_b                      3.231492
_cell_length_c                      40.000000
_cell_angle_alpha                   90.000000
_cell_angle_beta                    90.000000
_cell_angle_gamma                   120.007370
_cell_volume                        361.718401
_space_group_name_H-M_alt           'P 1'
_space_group_IT_number              1

loop_
_space_group_symop_operation_xyz
   'x, y, z'

loop_
   _atom_site_label
   _atom_site_occupancy
   _atom_site_fract_x
   _atom_site_fract_y
   _atom_site_fract_z
   _atom_site_adp_type
   _atom_site_B_iso_or_equiv
   _atom_site_type_symbol
   MO001      1.0    0.356211   -0.304517    0.289685   Biso  1.000000 Mo
   S002       1.0   -0.310442    0.362145    0.252189   Biso  1.000000 S
   S003       1.0   -0.310454    0.362110    0.327198   Biso  1.000000 S
   TE004      1.0    0.356680   -0.304242    0.396011   Biso  1.000000 Te
   TE005      1.0    0.356673   -0.304240    0.488725   Biso  1.000000 Te
   W006       1.0   -0.310018    0.362433    0.442293   Biso  1.000000 W
```

78. 1H-MoS_2/WTe_2-60

```
_cell_length_a                         3.232680
_cell_length_b                         3.216189
_cell_length_c                         40.000000
_cell_angle_alpha                      90.000000
_cell_angle_beta                       90.000000
_cell_angle_gamma                      60.103893
_cell_volume                           360.535979
_space_group_name_H-M_alt              'P 1'
_space_group_IT_number                 1

loop_
_space_group_symop_operation_xyz
   'x, y, z'

loop_
   _atom_site_label
   _atom_site_occupancy
   _atom_site_fract_x
   _atom_site_fract_y
   _atom_site_fract_z
   _atom_site_adp_type
   _atom_site_B_iso_or_equiv
   _atom_site_type_symbol
   MO001       1.0    -0.057153    -0.411332     0.285114    Biso  1.000000 Mo
   S002        1.0    -0.392087     0.256410     0.247610    Biso  1.000000 S
   S003        1.0    -0.389363     0.255097     0.322559    Biso  1.000000 S
   TE004       1.0    -0.006460     0.457081     0.395527    Biso  1.000000 Te
   TE005       1.0    -0.000838     0.455047     0.488421    Biso  1.000000 Te
   W006        1.0     0.338791    -0.206683     0.441868    Biso  1.000000 W
```

79. $MoSe_2/MoTe_2$-0

```
_cell_length_a                        3.306089
_cell_length_b                        3.305607
_cell_length_c                        40.000000
_cell_angle_alpha                     90.000000
_cell_angle_beta                      90.000000
_cell_angle_gamma                     119.996788
_cell_volume                          378.591134
_space_group_name_H-M_alt             'P 1'
_space_group_IT_number                1

loop_
_space_group_symop_operation_xyz
   'x, y, z'

loop_
   _atom_site_label
   _atom_site_occupancy
   _atom_site_fract_x
   _atom_site_fract_y
   _atom_site_fract_z
   _atom_site_adp_type
   _atom_site_B_iso_or_equiv
   _atom_site_type_symbol
   MO001      1.0     0.353418    -0.317534     0.300159    Biso  1.000000 Mo
   SE002      1.0     0.019922     0.015710     0.340020    Biso  1.000000 Se
   SE003      1.0     0.020059     0.015804     0.259937    Biso  1.000000 Se
   TE004      1.0    -0.314373     0.348767     0.411515    Biso  1.000000 Te
   TE005      1.0    -0.314446     0.348714     0.501666    Biso  1.000000 Te
   MO006      1.0     0.352210    -0.317961     0.456382    Biso  1.000000 Mo
```

80. $MoSe_2/MoTe_2$-60

```
_cell_length_a                       3.301309
_cell_length_b                       3.301306
_cell_length_c                       40.000000
_cell_angle_alpha                    90.000000
_cell_angle_beta                     90.000000
_cell_angle_gamma                    59.994431
_cell_volume                         377.518483
_space_group_name_H-M_alt            'P 1'
_space_group_IT_number               1

loop_
_space_group_symop_operation_xyz
   'x, y, z'

loop_
   _atom_site_label
   _atom_site_occupancy
   _atom_site_fract_x
   _atom_site_fract_y
   _atom_site_fract_z
   _atom_site_adp_type
   _atom_site_B_iso_or_equiv
   _atom_site_type_symbol
   MO001       1.0    -0.349068    -0.313673     0.300113    Biso  1.000000 Mo
   SE002       1.0    -0.015713     0.019607     0.340091    Biso  1.000000 Se
   SE003       1.0    -0.015751     0.019674     0.259955    Biso  1.000000 Se
   TE004       1.0    -0.348834    -0.313962     0.411441    Biso  1.000000 Te
   TE005       1.0    -0.348780    -0.314057     0.501701    Biso  1.000000 Te
   MO006       1.0     0.317905     0.352682     0.456378    Biso  1.000000 Mo
```

81. $MoSe_2/WS_2$-0

```
_cell_length_a                       3.147996
_cell_length_b                       3.147840
_cell_length_c                       40.000000
_cell_angle_alpha                    90.000000
_cell_angle_beta                     90.000000
_cell_angle_gamma                    119.997581
_cell_volume                         343.279623
_space_group_name_H-M_alt            'P 1'
_space_group_IT_number               1

loop_
_space_group_symop_operation_xyz
   'x, y, z'

loop_
   _atom_site_label
   _atom_site_occupancy
   _atom_site_fract_x
   _atom_site_fract_y
   _atom_site_fract_z
   _atom_site_adp_type
   _atom_site_B_iso_or_equiv
   _atom_site_type_symbol
   MO001       1.0    -0.341536     0.326669     0.303432    Biso  1.000000 Mo
   SE002       1.0    -0.008178    -0.006658     0.344798    Biso  1.000000 Se
   SE003       1.0    -0.008188    -0.006659     0.261762    Biso  1.000000 Se
   W004        1.0     0.324778    -0.340090     0.448019    Biso  1.000000 W
   S005        1.0    -0.341849     0.326595     0.486481    Biso  1.000000 S
   S006        1.0    -0.341828     0.326604     0.409789    Biso  1.000000 S
```

82. $MoSe_2/WS_2$-60

```
_cell_length_a                        3.152209
_cell_length_b                        3.152258
_cell_length_c                        40.000000
_cell_angle_alpha                     90.000000
_cell_angle_beta                      90.000000
_cell_angle_gamma                     60.005825
_cell_volume                          344.233289
_space_group_name_H-M_alt             'P 1'
_space_group_IT_number                1

loop_
_space_group_symop_operation_xyz
   'x, y, z'

loop_
   _atom_site_label
   _atom_site_occupancy
   _atom_site_fract_x
   _atom_site_fract_y
   _atom_site_fract_z
   _atom_site_adp_type
   _atom_site_B_iso_or_equiv
   _atom_site_type_symbol
   MO001       1.0    -0.324918    -0.340568     0.297036    Biso  1.000000 Mo
   SE002       1.0     0.008430    -0.007225     0.338300    Biso  1.000000 Se
   SE003       1.0     0.008465    -0.007232     0.255318    Biso  1.000000 Se
   W004        1.0    -0.325039    -0.339462     0.441938    Biso  1.000000 W
   S005        1.0     0.341622     0.327216     0.480385    Biso  1.000000 S
   S006        1.0     0.341539     0.327192     0.403803    Biso  1.000000 S
```

83. $MoSe_2/WSe_2$-0

```
_cell_length_a                        3.197184
_cell_length_b                        3.197070
_cell_length_c                        40.000000
_cell_angle_alpha                     90.000000
_cell_angle_beta                      90.000000
_cell_angle_gamma                     119.998726
_cell_volume                          354.091882
_space_group_name_H-M_alt             'P 1'
_space_group_IT_number                1

loop_
_space_group_symop_operation_xyz
   'x, y, z'

loop_
   _atom_site_label
   _atom_site_occupancy
   _atom_site_fract_x
   _atom_site_fract_y
   _atom_site_fract_z
   _atom_site_adp_type
   _atom_site_B_iso_or_equiv
   _atom_site_type_symbol
   MO001       1.0     0.333654    -0.332907     0.295771    Biso  1.000000 Mo
   SE002       1.0     0.000303     0.000415     0.336713    Biso  1.000000 Se
   SE003       1.0     0.000307     0.000424     0.254653    Biso  1.000000 Se
   W004        1.0    -0.332939     0.333846     0.445789    Biso  1.000000 W
   SE005       1.0     0.333712    -0.332829     0.404526    Biso  1.000000 Se
   SE006       1.0     0.333715    -0.332829     0.487409    Biso  1.000000 Se
```

84. $MoSe_2/WSe_2$-60

```
_cell_length_a                       3.202032
_cell_length_b                       9.606079
_cell_length_c                       40.000000
_cell_angle_alpha                    90.000000
_cell_angle_beta                     90.000000
_cell_angle_gamma                    60.001083
_cell_volume                         1065.533731
_space_group_name_H-M_alt            'P 1'
_space_group_IT_number               1

loop_
_space_group_symop_operation_xyz
   'x, y, z'

loop_
   _atom_site_label
   _atom_site_occupancy
   _atom_site_fract_x
   _atom_site_fract_y
   _atom_site_fract_z
   _atom_site_adp_type
   _atom_site_B_iso_or_equiv
   _atom_site_type_symbol
   MO001       1.0    -0.333850     0.222352     0.295963    Biso  1.000000 Mo
   MO002       1.0    -0.333846    -0.444329     0.295963    Biso  1.000000 Mo
   MO003       1.0    -0.333868    -0.110982     0.295962    Biso  1.000000 Mo
   SE004       1.0    -0.000491     0.333455     0.336776    Biso  1.000000 Se
   SE005       1.0    -0.000527    -0.333204     0.336774    Biso  1.000000 Se
   SE006       1.0    -0.000520     0.000125     0.336775    Biso  1.000000 Se
   SE007       1.0    -0.000507     0.333458     0.254810    Biso  1.000000 Se
   SE008       1.0    -0.000526    -0.333207     0.254813    Biso  1.000000 Se
   SE009       1.0    -0.000530     0.000130     0.254811    Biso  1.000000 Se
   W010        1.0    -0.333752     0.222338     0.445638    Biso  1.000000 W
   W011        1.0    -0.333740    -0.444327     0.445638    Biso  1.000000 W
   W012        1.0    -0.333770    -0.110975     0.445637    Biso  1.000000 W
   SE013       1.0     0.332885     0.111234     0.404441    Biso  1.000000 Se
   SE014       1.0     0.332916     0.444561     0.404442    Biso  1.000000 Se
   SE015       1.0     0.332904    -0.222098     0.404443    Biso  1.000000 Se
   SE016       1.0     0.332900     0.111233     0.487232    Biso  1.000000 Se
   SE017       1.0     0.332926     0.444556     0.487231    Biso  1.000000 Se
   SE018       1.0     0.332908    -0.222098     0.487229    Biso  1.000000 Se
```

85. $MoSe_2/WTe_2$-0

```
_cell_length_a                       3.297148
_cell_length_b                       9.891520
_cell_length_c                       40.000000
_cell_angle_alpha                    90.000000
_cell_angle_beta                     90.000000
_cell_angle_gamma                    119.999855
_cell_volume                         1129.776954
_space_group_name_H-M_alt            'P 1'
_space_group_IT_number               1

loop_
_space_group_symop_operation_xyz
   'x, y, z'

loop_
   _atom_site_label
   _atom_site_occupancy
   _atom_site_fract_x
   _atom_site_fract_y
   _atom_site_fract_z
   _atom_site_adp_type
   _atom_site_B_iso_or_equiv
   _atom_site_type_symbol
   MO001      1.0    0.348020    0.228334    0.299968   Biso  1.000000 Mo
   MO002      1.0    0.348010   -0.438340    0.299964   Biso  1.000000 Mo
   MO003      1.0    0.348015   -0.105002    0.299964   Biso  1.000000 Mo
   SE004      1.0    0.014695    0.339456    0.339977   Biso  1.000000 Se
   SE005      1.0    0.014677   -0.327229    0.339972   Biso  1.000000 Se
   SE006      1.0    0.014666    0.006100    0.339974   Biso  1.000000 Se
   SE007      1.0    0.014663    0.339430    0.259767   Biso  1.000000 Se
   SE008      1.0    0.014685   -0.327225    0.259764   Biso  1.000000 Se
   SE009      1.0    0.014695    0.006117    0.259765   Biso  1.000000 Se
   TE010      1.0    0.347882    0.228310    0.411670   Biso  1.000000 Te
   TE011      1.0    0.347899   -0.438341    0.411670   Biso  1.000000 Te
   TE012      1.0    0.347894   -0.105015    0.411672   Biso  1.000000 Te
   TE013      1.0    0.347887    0.228323    0.502629   Biso  1.000000 Te
   TE014      1.0    0.347888   -0.438351    0.502629   Biso  1.000000 Te
   TE015      1.0    0.347895   -0.105014    0.502630   Biso  1.000000 Te
   W016       1.0   -0.318784    0.117205    0.456955   Biso  1.000000 W
   W017       1.0   -0.318770    0.450541    0.456953   Biso  1.000000 W
   W018       1.0   -0.318778   -0.216130    0.456955   Biso  1.000000 W
```

86. $MoSe_2/WTe_2$-60

```
_cell_length_a                         3.301393
_cell_length_b                         3.301362
_cell_length_c                         40.000000
_cell_angle_alpha                      90.000000
_cell_angle_beta                       90.000000
_cell_angle_gamma                      59.998203
_cell_volume                           377.548843
_space_group_name_H-M_alt              'P 1'
_space_group_IT_number                 1

loop_
_space_group_symop_operation_xyz
   'x, y, z'

loop_
   _atom_site_label
   _atom_site_occupancy
   _atom_site_fract_x
   _atom_site_fract_y
   _atom_site_fract_z
   _atom_site_adp_type
   _atom_site_B_iso_or_equiv
   _atom_site_type_symbol
   MO001       1.0    -0.351483    -0.318712     0.293774    Biso  1.000000 Mo
   SE002       1.0    -0.018079     0.014564     0.333672    Biso  1.000000 Se
   SE003       1.0    -0.018152     0.014614     0.253505    Biso  1.000000 Se
   TE004       1.0     0.314841     0.347999     0.405472    Biso  1.000000 Te
   TE005       1.0     0.314848     0.348016     0.496343    Biso  1.000000 Te
   W006        1.0    -0.351844    -0.318621     0.450693    Biso  1.000000 W
```

87. $MoTe_2/WS_2$-0

```
_cell_length_a                       3.234512
_cell_length_b                       3.234491
_cell_length_c                       40.000000
_cell_angle_alpha                    90.000000
_cell_angle_beta                     90.000000
_cell_angle_gamma                    119.999413
_cell_volume                         362.416477
_space_group_name_H-M_alt            'P 1'
_space_group_IT_number               1

loop_
_space_group_symop_operation_xyz
   'x, y, z'

loop_
   _atom_site_label
   _atom_site_occupancy
   _atom_site_fract_x
   _atom_site_fract_y
   _atom_site_fract_z
   _atom_site_adp_type
   _atom_site_B_iso_or_equiv
   _atom_site_type_symbol
   TE001       1.0     0.307662    -0.353976     0.258316    Biso  1.000000 Te
   TE002       1.0     0.307671    -0.353976     0.350358    Biso  1.000000 Te
   MO003       1.0    -0.359017     0.312675     0.304432    Biso  1.000000 Mo
   W004        1.0     0.306971    -0.354309     0.456967    Biso  1.000000 W
   S005        1.0    -0.359685     0.312363     0.494599    Biso  1.000000 S
   S006        1.0    -0.359673     0.312352     0.419309    Biso  1.000000 S
```

88. $MoTe_2/WS_2$-60

```
_cell_length_a                         3.242615
_cell_length_b                         3.242645
_cell_length_c                         40.000000
_cell_angle_alpha                      90.000000
_cell_angle_beta                       90.000000
_cell_angle_gamma                      60.002800
_cell_volume                           364.248414
_space_group_name_H-M_alt              'P 1'
_space_group_IT_number                 1

loop_
_space_group_symop_operation_xyz
   'x, y, z'

loop_
   _atom_site_label
   _atom_site_occupancy
   _atom_site_fract_x
   _atom_site_fract_y
   _atom_site_fract_z
   _atom_site_adp_type
   _atom_site_B_iso_or_equiv
   _atom_site_type_symbol
   TE001       1.0    -0.307340    -0.354235     0.259736    Biso  1.000000 Te
   TE002       1.0    -0.307343    -0.354224     0.351528    Biso  1.000000 Te
   MO003       1.0     0.026006    -0.020862     0.305768    Biso  1.000000 Mo
   W004        1.0     0.359371     0.312603     0.455589    Biso  1.000000 W
   S005        1.0     0.026032    -0.020741     0.493238    Biso  1.000000 S
   S006        1.0     0.025993    -0.020721     0.418121    Biso  1.000000 S
```

89. $MoTe_2/WSe_2$-0

```
_cell_length_a                      3.293620
_cell_length_b                      3.293590
_cell_length_c                      40.000000
_cell_angle_alpha                   90.000000
_cell_angle_beta                    90.000000
_cell_angle_gamma                   120.001060
_cell_volume                        375.775994
_space_group_name_H-M_alt           'P 1'
_space_group_IT_number              1

loop_
_space_group_symop_operation_xyz
   'x, y, z'

loop_
   _atom_site_label
   _atom_site_occupancy
   _atom_site_fract_x
   _atom_site_fract_y
   _atom_site_fract_z
   _atom_site_adp_type
   _atom_site_B_iso_or_equiv
   _atom_site_type_symbol
   TE001       1.0     0.315513    -0.347563     0.258304    Biso  1.000000 Te
   TE002       1.0     0.315509    -0.347585     0.348828    Biso  1.000000 Te
   MO003       1.0    -0.351149     0.319110     0.303657    Biso  1.000000 Mo
   W004        1.0     0.315619    -0.347557     0.460374    Biso  1.000000 W
   SE005       1.0    -0.351105     0.319132     0.419902    Biso  1.000000 Se
   SE006       1.0    -0.351017     0.319103     0.500996    Biso  1.000000 Se
```

90. $MoTe_2/WSe_2$-60

```
_cell_length_a                         3.299885
_cell_length_b                         9.899863
_cell_length_c                         40.000000
_cell_angle_alpha                      90.000000
_cell_angle_beta                       90.000000
_cell_angle_gamma                      60.000114
_cell_volume                           1131.668243
_space_group_name_H-M_alt              'P 1'
_space_group_IT_number                 1

loop_
_space_group_symop_operation_xyz
   'x, y, z'

loop_
   _atom_site_label
   _atom_site_occupancy
   _atom_site_fract_x
   _atom_site_fract_y
   _atom_site_fract_z
   _atom_site_adp_type
   _atom_site_B_iso_or_equiv
   _atom_site_type_symbol
   TE001       1.0     0.017767     0.161920     0.258226    Biso  1.000000 Te
   TE002       1.0     0.017789     0.495261     0.258229    Biso  1.000000 Te
   TE003       1.0     0.017751    -0.171406     0.258224    Biso  1.000000 Te
   TE004       1.0     0.017749     0.161926     0.348519    Biso  1.000000 Te
   TE005       1.0     0.017791     0.495258     0.348518    Biso  1.000000 Te
   TE006       1.0     0.017738    -0.171402     0.348521    Biso  1.000000 Te
   MO007       1.0     0.351079     0.273034     0.303568    Biso  1.000000 Mo
   MO008       1.0     0.351090    -0.393613     0.303569    Biso  1.000000 Mo
   MO009       1.0     0.351078    -0.060306     0.303569    Biso  1.000000 Mo
   W010        1.0     0.017792     0.161925     0.460532    Biso  1.000000 W
   W011        1.0     0.017792     0.495259     0.460530    Biso  1.000000 W
   W012        1.0     0.017764    -0.171407     0.460529    Biso  1.000000 W
   SE013       1.0    -0.315552     0.050820     0.420127    Biso  1.000000 Se
   SE014       1.0    -0.315541     0.384143     0.420126    Biso  1.000000 Se
   SE015       1.0    -0.315566    -0.282520     0.420126    Biso  1.000000 Se
   SE016       1.0    -0.315557     0.050814     0.501090    Biso  1.000000 Se
   SE017       1.0    -0.315567     0.384155     0.501089    Biso  1.000000 Se
   SE018       1.0    -0.315587    -0.282511     0.501088    Biso  1.000000 Se
```

91. $MoTe_2/WTe_2$-0

```
_cell_length_a                         10.218287
_cell_length_b                         10.218036
_cell_length_c                         40.000000
_cell_angle_alpha                      90.000000
_cell_angle_beta                       90.000000
_cell_angle_gamma                      119.999321
_cell_volume                           3616.921524
_space_group_name_H-M_alt              'P 1'
_space_group_IT_number                 1

loop_
_space_group_symop_operation_xyz
   'x, y, z'

loop_
   _atom_site_label
   _atom_site_occupancy
   _atom_site_fract_x
   _atom_site_fract_y
   _atom_site_fract_z
   _atom_site_adp_type
   _atom_site_B_iso_or_equiv
   _atom_site_type_symbol
   Te1        1.0     0.110733     0.221920     0.258472    Biso  1.000000 Te
   Te2        1.0     0.444069     0.221917     0.258471    Biso  1.000000 Te
   Te3        1.0    -0.222599     0.221919     0.258517    Biso  1.000000 Te
   Te4        1.0     0.110743    -0.444741     0.258472    Biso  1.000000 Te
   Te5        1.0     0.444071    -0.444750     0.258518    Biso  1.000000 Te
   Te6        1.0    -0.222594    -0.444742     0.258473    Biso  1.000000 Te
   Te7        1.0     0.110734    -0.111418     0.258517    Biso  1.000000 Te
   Te8        1.0     0.444068    -0.111411     0.258472    Biso  1.000000 Te
   Te9        1.0    -0.222592    -0.111409     0.258472    Biso  1.000000 Te
   Te10       1.0     0.110738     0.221914     0.346531    Biso  1.000000 Te
   Te11       1.0     0.444059     0.221913     0.346532    Biso  1.000000 Te
   Te12       1.0    -0.222594     0.221930     0.346492    Biso  1.000000 Te
   Te13       1.0     0.110732    -0.444749     0.346532    Biso  1.000000 Te
   Te14       1.0     0.444076    -0.444743     0.346489    Biso  1.000000 Te
   Te15       1.0    -0.222592    -0.444750     0.346531    Biso  1.000000 Te
   Te16       1.0     0.110739    -0.111406     0.346492    Biso  1.000000 Te
   Te17       1.0     0.444075    -0.111417     0.346531    Biso  1.000000 Te
   Te18       1.0    -0.222606    -0.111415     0.346532    Biso  1.000000 Te
   Mo1        1.0     0.221923     0.110956     0.302561    Biso  1.000000 Mo
   Mo2        1.0    -0.444964     0.110738     0.302563    Biso  1.000000 Mo
   Mo3        1.0    -0.111413     0.110736     0.302564    Biso  1.000000 Mo
   Mo4        1.0     0.221700     0.444069     0.302563    Biso  1.000000 Mo
   Mo5        1.0    -0.444741     0.444061     0.302563    Biso  1.000000 Mo
   Mo6        1.0    -0.111404     0.444293     0.302561    Biso  1.000000 Mo
   Mo7        1.0     0.221924    -0.222595     0.302564    Biso  1.000000 Mo
   Mo8        1.0    -0.444737    -0.222370     0.302561    Biso  1.000000 Mo
   Mo9        1.0    -0.111631    -0.222592     0.302564    Biso  1.000000 Mo
   Te19       1.0     0.221865     0.110820     0.420123    Biso  1.000000 Te
   Te20       1.0    -0.444801     0.110825     0.420082    Biso  1.000000 Te
   Te21       1.0    -0.111467     0.110823     0.420084    Biso  1.000000 Te
   Te22       1.0     0.221863     0.444152     0.420082    Biso  1.000000 Te
   Te23       1.0    -0.444804     0.444155     0.420083    Biso  1.000000 Te
   Te24       1.0    -0.111467     0.444157     0.420123    Biso  1.000000 Te
   Te25       1.0     0.221862    -0.222512     0.420084    Biso  1.000000 Te
   Te26       1.0    -0.444805    -0.222507     0.420125    Biso  1.000000 Te
   Te27       1.0    -0.111473    -0.222514     0.420082    Biso  1.000000 Te
   Te28       1.0     0.221862     0.110816     0.508630    Biso  1.000000 Te
   Te29       1.0    -0.444795     0.110829     0.508675    Biso  1.000000 Te
   Te30       1.0    -0.111471     0.110827     0.508674    Biso  1.000000 Te
   Te31       1.0     0.221865     0.444154     0.508675    Biso  1.000000 Te
   Te32       1.0    -0.444805     0.444159     0.508674    Biso  1.000000 Te
   Te33       1.0    -0.111470     0.444153     0.508630    Biso  1.000000 Te
   Te34       1.0     0.221857    -0.222511     0.508673    Biso  1.000000 Te
   Te35       1.0    -0.444808    -0.222515     0.508629    Biso  1.000000 Te
   Te36       1.0    -0.111469    -0.222512     0.508675    Biso  1.000000 Te
```

```
W1          1.0     0.110684     0.222004     0.464337    Biso  1.000000 W
W2          1.0     0.444226     0.222000     0.464337    Biso  1.000000 W
W3          1.0    -0.222656     0.221792     0.464335    Biso  1.000000 W
W4          1.0     0.110893    -0.444671     0.464337    Biso  1.000000 W
W5          1.0     0.444003    -0.444883     0.464335    Biso  1.000000 W
W6          1.0    -0.222652    -0.444663     0.464337    Biso  1.000000 W
W7          1.0     0.110670    -0.111546     0.464335    Biso  1.000000 W
W9          1.0    -0.222438    -0.111331     0.464337    Biso  1.000000 W
```

92. $MoTe_2/WTe_2$-60

```
_cell_length_a                          10.209053
_cell_length_b                          3.403085
_cell_length_c                          40.000000
_cell_angle_alpha                       90.000000
_cell_angle_beta                        90.000000
_cell_angle_gamma                       60.010693
_cell_volume                            1203.637368
_space_group_name_H-M_alt               'P 1'
_space_group_IT_number                  1

loop_
_space_group_symop_operation_xyz
   'x, y, z'

loop_
   _atom_site_label
   _atom_site_occupancy
   _atom_site_fract_x
   _atom_site_fract_y
   _atom_site_fract_z
   _atom_site_adp_type
   _atom_site_B_iso_or_equiv
   _atom_site_type_symbol
   TE001       1.0     0.103106     0.343597     0.248161    Biso  1.000000 Te
   TE002       1.0     0.436523     0.343480     0.248168    Biso  1.000000 Te
   TE003       1.0    -0.230050     0.343323     0.248174    Biso  1.000000 Te
   TE004       1.0     0.102878     0.343881     0.336301    Biso  1.000000 Te
   TE005       1.0     0.436215     0.343876     0.336281    Biso  1.000000 Te
   TE006       1.0    -0.230460     0.343880     0.336318    Biso  1.000000 Te
   MO007       1.0    -0.119172    -0.323004     0.292289    Biso  1.000000 Mo
   MO008       1.0     0.214113    -0.322924     0.292270    Biso  1.000000 Mo
   MO009       1.0    -0.452434    -0.323099     0.292267    Biso  1.000000 Mo
   TE010       1.0    -0.213346     0.321154     0.430309    Biso  1.000000 Te
   TE011       1.0     0.453518     0.320826     0.430348    Biso  1.000000 Te
   TE012       1.0     0.120041     0.321055     0.430275    Biso  1.000000 Te
   TE013       1.0    -0.213410     0.321194     0.519010    Biso  1.000000 Te
   TE014       1.0     0.452960     0.321610     0.519020    Biso  1.000000 Te
   TE015       1.0     0.119711     0.321491     0.518982    Biso  1.000000 Te
   W016        1.0    -0.102298    -0.345400     0.474584    Biso  1.000000 W
   W017        1.0    -0.435592    -0.345484     0.474639    Biso  1.000000 W
   W018        1.0     0.230945    -0.345285     0.474584    Biso  1.000000 W
```

93. WSe_2/WS_2-0

```
_cell_length_a                        3.151236
_cell_length_b                        3.151212
_cell_length_c                        40.000000
_cell_angle_alpha                     90.000000
_cell_angle_beta                      90.000000
_cell_angle_gamma                     119.999458
_cell_volume                          343.994540
_space_group_name_H-M_alt             'P 1'
_space_group_IT_number                1

loop_
_space_group_symop_operation_xyz
   'x, y, z'

loop_
   _atom_site_label
   _atom_site_occupancy
   _atom_site_fract_x
   _atom_site_fract_y
   _atom_site_fract_z
   _atom_site_adp_type
   _atom_site_B_iso_or_equiv
   _atom_site_type_symbol
   W001        1.0    -0.173588    -0.342062     0.296467    Biso  1.000000 W
   SE002       1.0     0.493080    -0.008724     0.254341    Biso  1.000000 Se
   SE003       1.0     0.493060    -0.008741     0.338147    Biso  1.000000 Se
   W004        1.0     0.492863    -0.008874     0.443149    Biso  1.000000 W
   S005        1.0     0.159528     0.324454     0.481501    Biso  1.000000 S
   S006        1.0     0.159488     0.324448     0.404855    Biso  1.000000 S
```

94. WSe_2/WS_2-60

```
_cell_length_a                         3.147007
_cell_length_b                         3.147288
_cell_length_c                         40.000000
_cell_angle_alpha                      90.000000
_cell_angle_beta                       90.000000
_cell_angle_gamma                      59.991055
_cell_volume                           343.072315
_space_group_name_H-M_alt              'P 1'
_space_group_IT_number                 1

loop_
_space_group_symop_operation_xyz
   'x, y, z'

loop_
   _atom_site_label
   _atom_site_occupancy
   _atom_site_fract_x
   _atom_site_fract_y
   _atom_site_fract_z
   _atom_site_adp_type
   _atom_site_B_iso_or_equiv
   _atom_site_type_symbol
   W001        1.0     0.341638     0.327305     0.295974    Biso  1.000000 W
   SE002       1.0    -0.325041    -0.339356     0.253865    Biso  1.000000 Se
   SE003       1.0    -0.325066    -0.339294     0.337783    Biso  1.000000 Se
   W004        1.0    -0.324026    -0.341390     0.443591    Biso  1.000000 W
   S005        1.0     0.342629     0.325264     0.481993    Biso  1.000000 S
   S006        1.0     0.342696     0.325232     0.405253    Biso  1.000000 S
```

95. WTe_2/WS_2-0

```
_cell_length_a                      3.241987
_cell_length_b                      3.242077
_cell_length_c                      40.000000
_cell_angle_alpha                   90.000000
_cell_angle_beta                    90.000000
_cell_angle_gamma                   120.001846
_cell_volume                        364.097042
_space_group_name_H-M_alt           'P 1'
_space_group_IT_number              1

loop_
_space_group_symop_operation_xyz
   'x, y, z'

loop_
   _atom_site_label
   _atom_site_occupancy
   _atom_site_fract_x
   _atom_site_fract_y
   _atom_site_fract_z
   _atom_site_adp_type
   _atom_site_B_iso_or_equiv
   _atom_site_type_symbol
   TE001       1.0    -0.353119     0.308557     0.253428    Biso  1.000000 Te
   TE002       1.0    -0.353101     0.308573     0.345833    Biso  1.000000 Te
   W003        1.0    -0.019840    -0.024777     0.299752    Biso  1.000000 W
   W004        1.0     0.313151    -0.358560     0.450062    Biso  1.000000 W
   S005        1.0    -0.020226    -0.025249     0.487723    Biso  1.000000 S
   S006        1.0    -0.020245    -0.025264     0.412582    Biso  1.000000 S
```

96. WTe_2/WS_2-60

```
_cell_length_a                         3.234417
_cell_length_b                         3.234476
_cell_length_c                         40.000000
_cell_angle_alpha                      90.000000
_cell_angle_beta                       90.000000
_cell_angle_gamma                      59.998131
_cell_volume                           362.395164
_space_group_name_H-M_alt              'P 1'
_space_group_IT_number                 1

loop_
_space_group_symop_operation_xyz
   'x, y, z'

loop_
   _atom_site_label
   _atom_site_occupancy
   _atom_site_fract_x
   _atom_site_fract_y
   _atom_site_fract_z
   _atom_site_adp_type
   _atom_site_B_iso_or_equiv
   _atom_site_type_symbol
   TE001       1.0    -0.353644    -0.308098     0.251966    Biso  1.000000 Te
   TE002       1.0    -0.353684    -0.308108     0.344619    Biso  1.000000 Te
   W003        1.0     0.313026     0.358576     0.298377    Biso  1.000000 W
   W004        1.0    -0.353030    -0.308558     0.451479    Biso  1.000000 W
   S005        1.0     0.313632     0.358122     0.489117    Biso  1.000000 S
   S006        1.0     0.313660     0.358136     0.413823    Biso  1.000000 S
```

97. WTe_2/WSe_2-0

```
_cell_length_a                        3.296351
_cell_length_b                        3.296314
_cell_length_c                        40.000000
_cell_angle_alpha                     90.000000
_cell_angle_beta                      90.000000
_cell_angle_gamma                     119.998665
_cell_volume                          376.407688
_space_group_name_H-M_alt             'P 1'
_space_group_IT_number                1

loop_
_space_group_symop_operation_xyz
   'x, y, z'

loop_
   _atom_site_label
   _atom_site_occupancy
   _atom_site_fract_x
   _atom_site_fract_y
   _atom_site_fract_z
   _atom_site_adp_type
   _atom_site_B_iso_or_equiv
   _atom_site_type_symbol
   TE001       1.0    -0.016686     0.486639     0.251689    Biso  1.000000 Te
   TE002       1.0    -0.016686     0.486645     0.342671    Biso  1.000000 Te
   W003        1.0    -0.350045    -0.180026     0.297374    Biso  1.000000 W
   W004        1.0    -0.016788     0.486569     0.455175    Biso  1.000000 W
   SE005       1.0     0.316570     0.153231     0.414744    Biso  1.000000 Se
   SE006       1.0     0.316554     0.153252     0.495766    Biso  1.000000 Se
```

98. WTe_2/WSe_2-60

```
_cell_length_a                      3.291198
_cell_length_b                      3.291196
_cell_length_c                      40.000000
_cell_angle_alpha                   90.000000
_cell_angle_beta                    90.000000
_cell_angle_gamma                   60.000031
_cell_volume                        375.230844
_space_group_name_H-M_alt           'P 1'
_space_group_IT_number              1

loop_
_space_group_symop_operation_xyz
   'x, y, z'

loop_
   _atom_site_label
   _atom_site_occupancy
   _atom_site_fract_x
   _atom_site_fract_y
   _atom_site_fract_z
   _atom_site_adp_type
   _atom_site_B_iso_or_equiv
   _atom_site_type_symbol
   TE001       1.0    -0.316600    -0.346707     0.251911    Biso  1.000000 Te
   TE002       1.0    -0.316601    -0.346705     0.343076    Biso  1.000000 Te
   W003        1.0     0.350055     0.319958     0.297579    Biso  1.000000 W
   W004        1.0    -0.316581    -0.346744     0.454902    Biso  1.000000 W
   SE005       1.0     0.350075     0.319931     0.414400    Biso  1.000000 Se
   SE006       1.0     0.350072     0.319928     0.495552    Biso  1.000000 Se
```

99. 1T-MoS_2/1T-MoS_2-AA

```
_cell_length_a                          3.135561
_cell_length_b                          5.430384
_cell_length_c                          40.000000
_cell_angle_alpha                       90.000000
_cell_angle_beta                        90.000000
_cell_angle_gamma                       29.998634
_cell_volume                            340.531955
_space_group_name_H-M_alt               'P 1'
_space_group_IT_number                  1

loop_
_space_group_symop_operation_xyz
   'x, y, z'

loop_
   _atom_site_label
   _atom_site_occupancy
   _atom_site_fract_x
   _atom_site_fract_y
   _atom_site_fract_z
   _atom_site_adp_type
   _atom_site_B_iso_or_equiv
   _atom_site_type_symbol
   MO001       1.0    -0.000075     0.333565     0.306518    Biso  1.000000 Mo
   S002        1.0    -0.000110     0.000035     0.345201    Biso  1.000000 S
   S003        1.0    -0.000332    -0.332961     0.267500    Biso  1.000000 S
   MO004       1.0     0.000080     0.333107     0.439441    Biso  1.000000 Mo
   S005        1.0     0.000336    -0.000379     0.478452    Biso  1.000000 S
   S006        1.0     0.000100    -0.333368     0.400768    Biso  1.000000 S
```

100. $1T-MoS_2/1T-MoS_2$-AB

```
_cell_length_a                      3.111315
_cell_length_b                      3.111269
_cell_length_c                      40.000000
_cell_angle_alpha                   90.000000
_cell_angle_beta                    90.000000
_cell_angle_gamma                   60.013111
_cell_volume                        335.374108
_space_group_name_H-M_alt           'P 1'
_space_group_IT_number              1

loop_
_space_group_symop_operation_xyz
   'x, y, z'

loop_
   _atom_site_label
   _atom_site_occupancy
   _atom_site_fract_x
   _atom_site_fract_y
   _atom_site_fract_z
   _atom_site_adp_type
   _atom_site_B_iso_or_equiv
   _atom_site_type_symbol
   MO001       1.0     0.333108     0.332575     0.287705    Biso  1.000000 Mo
   S002        1.0    -0.000291    -0.000836     0.326786    Biso  1.000000 S
   S003        1.0    -0.333466    -0.333981     0.248430    Biso  1.000000 S
   MO004       1.0    -0.333235    -0.332393     0.425772    Biso  1.000000 Mo
   S005        1.0     0.000067     0.000869     0.465022    Biso  1.000000 S
   S006        1.0     0.333237     0.334276     0.386665    Biso  1.000000 S
```

101. 1H-MoS_2/1H-MoS_2-AA

```
_cell_length_a                      3.085509
_cell_length_b                      3.085124
_cell_length_c                      40.000000
_cell_angle_alpha                   90.000000
_cell_angle_beta                    90.000000
_cell_angle_gamma                   119.996323
_cell_volume                        329.766221
_space_group_name_H-M_alt           'P 1'
_space_group_IT_number              1

loop_
_space_group_symop_operation_xyz
   'x, y, z'

loop_
   _atom_site_label
   _atom_site_occupancy
   _atom_site_fract_x
   _atom_site_fract_y
   _atom_site_fract_z
   _atom_site_adp_type
   _atom_site_B_iso_or_equiv
   _atom_site_type_symbol
   MO001     1.0    -0.333309     0.333346     0.282030    Biso  1.000000 Mo
   S002      1.0     0.333326    -0.333337     0.243218    Biso  1.000000 S
   S003      1.0     0.333320    -0.333343     0.320679    Biso  1.000000 S
   MO004     1.0    -0.333307     0.333349     0.439363    Biso  1.000000 Mo
   S005      1.0     0.333321    -0.333341     0.400714    Biso  1.000000 S
   S006      1.0     0.333328    -0.333334     0.478175    Biso  1.000000 S
```

102. 1H-MoS_2/1H-MoS_2-AB

```
_cell_length_a                        5.359253
_cell_length_b                        3.093998
_cell_length_c                        40.000000
_cell_angle_alpha                     90.000000
_cell_angle_beta                      90.000000
_cell_angle_gamma                     30.003859
_cell_volume                          331.669034
_space_group_name_H-M_alt             'P 1'
_space_group_IT_number                1

loop_
_space_group_symop_operation_xyz
   'x, y, z'

loop_
   _atom_site_label
   _atom_site_occupancy
   _atom_site_fract_x
   _atom_site_fract_y
   _atom_site_fract_z
   _atom_site_adp_type
   _atom_site_B_iso_or_equiv
   _atom_site_type_symbol
   MO001       1.0     0.333325     0.000016     0.286999    Biso  1.000000 Mo
   S002        1.0    -0.333310    -0.000040     0.248250    Biso  1.000000 S
   S003        1.0    -0.333294    -0.000065     0.325634    Biso  1.000000 S
   MO004       1.0    -0.333419     0.000111     0.429395    Biso  1.000000 Mo
   S005        1.0     0.333201     0.000191     0.390759    Biso  1.000000 S
   S006        1.0     0.333216     0.000167     0.468143    Biso  1.000000 S
```

103. $MoSe_2/MoSe_2$-AA

```
_cell_length_a                        3.187201
_cell_length_b                        3.187034
_cell_length_c                        40.000000
_cell_angle_alpha                     90.000000
_cell_angle_beta                      90.000000
_cell_angle_gamma                     119.998207
_cell_volume                          351.880019
_space_group_name_H-M_alt             'P 1'
_space_group_IT_number                1

loop_
_space_group_symop_operation_xyz
   'x, y, z'

loop_
   _atom_site_label
   _atom_site_occupancy
   _atom_site_fract_x
   _atom_site_fract_y
   _atom_site_fract_z
   _atom_site_adp_type
   _atom_site_B_iso_or_equiv
   _atom_site_type_symbol
   MO001       1.0     0.333344    -0.333326     0.294337    Biso  1.000000 Mo
   SE002       1.0    -0.000009    -0.000002     0.335434    Biso  1.000000 Se
   SE003       1.0    -0.000004     0.000000     0.253135    Biso  1.000000 Se
   MO004       1.0     0.333343    -0.333327     0.459343    Biso  1.000000 Mo
   SE005       1.0    -0.000005    -0.000001     0.500545    Biso  1.000000 Se
   SE006       1.0    -0.000010    -0.000004     0.418246    Biso  1.000000 Se
```

104. $MoSe_2/MoSe_2$-AB

```
_cell_length_a                  3.193466
_cell_length_b                  5.531419
_cell_length_c                  40.000000
_cell_angle_alpha               90.000000
_cell_angle_beta                90.000000
_cell_angle_gamma               89.998688
_cell_volume                    706.575903
_space_group_name_H-M_alt       'P 1'
_space_group_IT_number          1

loop_
_space_group_symop_operation_xyz
   'x, y, z'

loop_
   _atom_site_label
   _atom_site_occupancy
   _atom_site_fract_x
   _atom_site_fract_y
   _atom_site_fract_z
   _atom_site_adp_type
   _atom_site_B_iso_or_equiv
   _atom_site_type_symbol
   MO001      1.0    -0.000034     0.499863     0.302192    Biso  1.000000 Mo
   MO002      1.0     0.499918    -0.000083     0.302189    Biso  1.000000 Mo
   SE003      1.0     0.499971    -0.333408     0.343110    Biso  1.000000 Se
   SE004      1.0    -0.000094     0.166567     0.343109    Biso  1.000000 Se
   SE005      1.0     0.499949    -0.333414     0.260983    Biso  1.000000 Se
   SE006      1.0    -0.000020     0.166562     0.260981    Biso  1.000000 Se
   MO007      1.0     0.000051     0.166765     0.451536    Biso  1.000000 Mo
   MO008      1.0    -0.499929    -0.333229     0.451534    Biso  1.000000 Mo
   SE009      1.0    -0.499945     0.333434     0.492676    Biso  1.000000 Se
   SE010      1.0     0.000045    -0.166561     0.492676    Biso  1.000000 Se
   SE011      1.0    -0.499978     0.333420     0.410549    Biso  1.000000 Se
   SE012      1.0     0.000104    -0.166555     0.410546    Biso  1.000000 Se
```

105. $MoTe_2/MoTe_2$-AA

```
_cell_length_a                         3.409921
_cell_length_b                         3.409813
_cell_length_c                         40.000000
_cell_angle_alpha                      90.000000
_cell_angle_beta                       90.000000
_cell_angle_gamma                      60.001015
_cell_volume                           402.781880
_space_group_name_H-M_alt              'P 1'
_space_group_IT_number                 1

loop_
_space_group_symop_operation_xyz
   'x, y, z'

loop_
   _atom_site_label
   _atom_site_occupancy
   _atom_site_fract_x
   _atom_site_fract_y
   _atom_site_fract_z
   _atom_site_adp_type
   _atom_site_B_iso_or_equiv
   _atom_site_type_symbol
   TE001       1.0     0.333333     0.333331     0.248176    Biso  1.000000 Te
   TE002       1.0     0.333333     0.333330     0.336158    Biso  1.000000 Te
   MO003       1.0    -0.333326    -0.333340     0.292210    Biso  1.000000 Mo
   TE004       1.0     0.333333     0.333330     0.430302    Biso  1.000000 Te
   TE005       1.0     0.333333     0.333330     0.518284    Biso  1.000000 Te
   MO006       1.0    -0.333326    -0.333341     0.474250    Biso  1.000000 Mo
```

106. $MoTe_2/MoTe_2$-AB

```
_cell_length_a                         5.909113
_cell_length_b                         3.411398
_cell_length_c                         40.000000
_cell_angle_alpha                      90.000000
_cell_angle_beta                       90.000000
_cell_angle_gamma                      30.006592
_cell_volume                           403.247049
_space_group_name_H-M_alt              'P 1'
_space_group_IT_number                 1

loop_
_space_group_symop_operation_xyz
   'x, y, z'

loop_
   _atom_site_label
   _atom_site_occupancy
   _atom_site_fract_x
   _atom_site_fract_y
   _atom_site_fract_z
   _atom_site_adp_type
   _atom_site_B_iso_or_equiv
   _atom_site_type_symbol
   TE001       1.0    -0.333156    -0.000270     0.258619    Biso  1.000000 Te
   TE002       1.0    -0.333125    -0.000316     0.346535    Biso  1.000000 Te
   MO003       1.0     0.333441    -0.000165     0.302634    Biso  1.000000 Mo
   TE004       1.0     0.333112     0.000331     0.419923    Biso  1.000000 Te
   TE005       1.0     0.333138     0.000292     0.507843    Biso  1.000000 Te
   MO006       1.0    -0.333460     0.000188     0.463826    Biso  1.000000 Mo
```

107. WS_2/WS_2-AA

```
_cell_length_a                        8.189524
_cell_length_b                        5.362077
_cell_length_c                        40.000000
_cell_angle_alpha                     90.000000
_cell_angle_beta                      90.000000
_cell_angle_gamma                     10.890669
_cell_volume                          331.867950
_space_group_name_H-M_alt             'P 1'
_space_group_IT_number                1

loop_
_space_group_symop_operation_xyz
   'x, y, z'

loop_
   _atom_site_label
   _atom_site_occupancy
   _atom_site_fract_x
   _atom_site_fract_y
   _atom_site_fract_z
   _atom_site_adp_type
   _atom_site_B_iso_or_equiv
   _atom_site_type_symbol
   W001        1.0     0.000066    -0.333423     0.293372    Biso  1.000000 W
   S002        1.0     0.000015     0.333320     0.332074    Biso  1.000000 S
   S003        1.0    -0.000072     0.333434     0.254447    Biso  1.000000 S
   W004        1.0     0.000066    -0.333424     0.451142    Biso  1.000000 W
   S005        1.0    -0.000071     0.333433     0.490066    Biso  1.000000 S
   S006        1.0     0.000015     0.333320     0.412439    Biso  1.000000 S
```

108. WS_2/WS_2-AB

```
_cell_length_a                          5.375331
_cell_length_b                          3.103524
_cell_length_c                          40.000000
_cell_angle_alpha                       90.000000
_cell_angle_beta                        90.000000
_cell_angle_gamma                       30.000853
_cell_volume                            333.657967
_space_group_name_H-M_alt               'P 1'
_space_group_IT_number                  1

loop_
_space_group_symop_operation_xyz
   'x, y, z'

loop_
   _atom_site_label
   _atom_site_occupancy
   _atom_site_fract_x
   _atom_site_fract_y
   _atom_site_fract_z
   _atom_site_adp_type
   _atom_site_B_iso_or_equiv
   _atom_site_type_symbol
   W001        1.0    -0.333594     0.000719     0.294562    Biso  1.000000 W
   S002        1.0     0.333038     0.000778     0.333220    Biso  1.000000 S
   S003        1.0     0.333041     0.000796     0.255720    Biso  1.000000 S
   W004        1.0     0.333594    -0.000713     0.437451    Biso  1.000000 W
   S005        1.0    -0.333041    -0.000789     0.476293    Biso  1.000000 S
   S006        1.0    -0.333037    -0.000771     0.398794    Biso  1.000000 S
```

109. WSe_2/WSe_2-AA

```
_cell_length_a                         3.195837
_cell_length_b                         3.195900
_cell_length_c                         40.000000
_cell_angle_alpha                      90.000000
_cell_angle_beta                       90.000000
_cell_angle_gamma                      120.000732
_cell_volume                           353.806020
_space_group_name_H-M_alt              'P 1'
_space_group_IT_number                 1

loop_
_space_group_symop_operation_xyz
   'x, y, z'

loop_
   _atom_site_label
   _atom_site_occupancy
   _atom_site_fract_x
   _atom_site_fract_y
   _atom_site_fract_z
   _atom_site_adp_type
   _atom_site_B_iso_or_equiv
   _atom_site_type_symbol
   W001        1.0     0.333676    -0.333431     0.294263    Biso  1.000000 W
   SE002       1.0    -0.333000     0.333241     0.252683    Biso  1.000000 Se
   SE003       1.0    -0.332985     0.333237     0.335682    Biso  1.000000 Se
   W004        1.0     0.332989    -0.333240     0.460417    Biso  1.000000 W
   SE005       1.0    -0.333675     0.333430     0.418999    Biso  1.000000 Se
   SE006       1.0    -0.333665     0.333423     0.501997    Biso  1.000000 Se
```

110. WSe_2/WSe_2-AB

```
_cell_length_a                        5.535478
_cell_length_b                        3.195963
_cell_length_c                        40.000000
_cell_angle_alpha                     90.000000
_cell_angle_beta                      90.000000
_cell_angle_gamma                     150.000092
_cell_volume                          353.822675
_space_group_name_H-M_alt             'P 1'
_space_group_IT_number                1

loop_
_space_group_symop_operation_xyz
   'x, y, z'

loop_
   _atom_site_label
   _atom_site_occupancy
   _atom_site_fract_x
   _atom_site_fract_y
   _atom_site_fract_z
   _atom_site_adp_type
   _atom_site_B_iso_or_equiv
   _atom_site_type_symbol
   W001        1.0    -0.333243     0.000143     0.295201    Biso  1.000000 W
   SE002       1.0     0.333444     0.000176     0.253639    Biso  1.000000 Se
   SE003       1.0     0.333451     0.000188     0.336587    Biso  1.000000 Se
   W004        1.0     0.333236    -0.000157     0.446979    Biso  1.000000 W
   SE005       1.0    -0.333456    -0.000199     0.405594    Biso  1.000000 Se
   SE006       1.0    -0.333453    -0.000192     0.488540    Biso  1.000000 Se
```

111. WTe_2/WTe_2-AA

```
_cell_length_a                        3.396668
_cell_length_b                        8.982443
_cell_length_c                        40.000000
_cell_angle_alpha                     90.000000
_cell_angle_beta                      90.000000
_cell_angle_gamma                     19.138248
_cell_volume                          400.111406
_space_group_name_H-M_alt             'P 1'
_space_group_IT_number                1

loop_
_space_group_symop_operation_xyz
   'x, y, z'

loop_
   _atom_site_label
   _atom_site_occupancy
   _atom_site_fract_x
   _atom_site_fract_y
   _atom_site_fract_z
   _atom_site_adp_type
   _atom_site_B_iso_or_equiv
   _atom_site_type_symbol
   TE001       1.0     0.333280    -0.333279     0.248249    Biso  1.000000 Te
   TE002       1.0     0.333275    -0.333276     0.337025    Biso  1.000000 Te
   W003        1.0    -0.333224     0.333224     0.292691    Biso  1.000000 W
   TE004       1.0     0.333275    -0.333275     0.430828    Biso  1.000000 Te
   TE005       1.0     0.333279    -0.333279     0.519604    Biso  1.000000 Te
   W006        1.0    -0.333225     0.333225     0.475163    Biso  1.000000 W
```

112. WTe_2/WTe_2-AB

```
_cell_length_a                        5.884704
_cell_length_b                        9.010520
_cell_length_c                        40.000000
_cell_angle_alpha                     90.000000
_cell_angle_beta                      90.000000
_cell_angle_gamma                     10.940047
_cell_volume                          402.521338
_space_group_name_H-M_alt             'P 1'
_space_group_IT_number                1

loop_
_space_group_symop_operation_xyz
   'x, y, z'

loop_
   _atom_site_label
   _atom_site_occupancy
   _atom_site_fract_x
   _atom_site_fract_y
   _atom_site_fract_z
   _atom_site_adp_type
   _atom_site_B_iso_or_equiv
   _atom_site_type_symbol
   TE001       1.0     0.331271     0.001541     0.252168    Biso  1.000000 Te
   TE002       1.0     0.330347     0.002234     0.340768    Biso  1.000000 Te
   W003        1.0    -0.341172     0.005876     0.296486    Biso  1.000000 W
   TE004       1.0    -0.330346    -0.002237     0.414567    Biso  1.000000 Te
   TE005       1.0    -0.331277    -0.001539     0.503201    Biso  1.000000 Te
   W006        1.0     0.341168    -0.005875     0.458869    Biso  1.000000 W
```

113. 1T-MoS_2

```
_cell_length_a                     3.090769
_cell_length_b                     3.090439
_cell_length_c                     40.000000
_cell_angle_alpha                  90.000000
_cell_angle_beta                   90.000000
_cell_angle_gamma                  120.014450
_cell_volume                       330.837027
_space_group_name_H-M_alt          'P 1'
_space_group_IT_number             1

loop_
_space_group_symop_operation_xyz
   'x, y, z'

loop_
   _atom_site_label
   _atom_site_occupancy
   _atom_site_fract_x
   _atom_site_fract_y
   _atom_site_fract_z
   _atom_site_adp_type
   _atom_site_B_iso_or_equiv
   _atom_site_type_symbol
   MO001       1.0     0.333331    -0.333334     0.289866    Biso  1.000000 Mo
   S002        1.0    -0.000004    -0.000058     0.329218    Biso  1.000000 S
   S003        1.0    -0.333327     0.333391     0.250513    Biso  1.000000 S
```

114. 1H-MoS_2

```
_cell_length_a                        3.083198
_cell_length_b                        3.082781
_cell_length_c                        40.000000
_cell_angle_alpha                     90.000000
_cell_angle_beta                      90.000000
_cell_angle_gamma                     119.995399
_cell_volume                          329.271971
_space_group_name_H-M_alt             'P 1'
_space_group_IT_number                1

loop_
_space_group_symop_operation_xyz
   'x, y, z'

loop_
   _atom_site_label
   _atom_site_occupancy
   _atom_site_fract_x
   _atom_site_fract_y
   _atom_site_fract_z
   _atom_site_adp_type
   _atom_site_B_iso_or_equiv
   _atom_site_type_symbol
   MO001       1.0     0.333311    -0.333345     0.289434    Biso  1.000000 Mo
   S002        1.0    -0.333322     0.333339     0.250683    Biso  1.000000 S
   S003        1.0    -0.333322     0.333339     0.328185    Biso  1.000000 S
```

115. $MoSe_2$

```
_cell_length_a                      3.193878
_cell_length_b                      3.193675
_cell_length_c                      40.000000
_cell_angle_alpha                   90.000000
_cell_angle_beta                    90.000000
_cell_angle_gamma                   119.997841
_cell_volume                        353.353266
_space_group_name_H-M_alt           'P 1'
_space_group_IT_number              1

loop_
_space_group_symop_operation_xyz
   'x, y, z'

loop_
   _atom_site_label
   _atom_site_occupancy
   _atom_site_fract_x
   _atom_site_fract_y
   _atom_site_fract_z
   _atom_site_adp_type
   _atom_site_B_iso_or_equiv
   _atom_site_type_symbol
   MO001       1.0     0.333346    -0.333327     0.291992    Biso  1.000000 Mo
   SE002       1.0    -0.000006    -0.000003     0.333070    Biso  1.000000 Se
   SE003       1.0    -0.000006    -0.000003     0.250913    Biso  1.000000 Se
```

116. $MoTe_2$

```
_cell_length_a                          3.417764
_cell_length_b                          3.417667
_cell_length_c                          40.000000
_cell_angle_alpha                       90.000000
_cell_angle_beta                        90.000000
_cell_angle_gamma                       119.999069
_cell_volume                            404.637841
_space_group_name_H-M_alt               'P 1'
_space_group_IT_number                  1

loop_
_space_group_symop_operation_xyz
   'x, y, z'

loop_
   _atom_site_label
   _atom_site_occupancy
   _atom_site_fract_x
   _atom_site_fract_y
   _atom_site_fract_z
   _atom_site_adp_type
   _atom_site_B_iso_or_equiv
   _atom_site_type_symbol
   TE001       1.0    -0.333331     0.333334     0.251540    Biso  1.000000 Te
   TE002       1.0    -0.333331     0.333334     0.339469    Biso  1.000000 Te
   MO003       1.0     0.333329    -0.333335     0.295504    Biso  1.000000 Mo
```

117. WS_2

```
_cell_length_a                         3.097692
_cell_length_b                         3.097669
_cell_length_c                         40.000000
_cell_angle_alpha                      90.000000
_cell_angle_beta                       90.000000
_cell_angle_gamma                      119.999641
_cell_volume                           332.403373
_space_group_name_H-M_alt              'P 1'
_space_group_IT_number                 1

loop_
_space_group_symop_operation_xyz
   'x, y, z'

loop_
   _atom_site_label
   _atom_site_occupancy
   _atom_site_fract_x
   _atom_site_fract_y
   _atom_site_fract_z
   _atom_site_adp_type
   _atom_site_B_iso_or_equiv
   _atom_site_type_symbol
   W001       1.0    -0.333323     0.333339     0.289662    Biso  1.000000 W
   S002       1.0     0.333328    -0.333336     0.328494    Biso  1.000000 S
   S003       1.0     0.333328    -0.333336     0.250830    Biso  1.000000 S
```

118. WSe_2

```
_cell_length_a                         3.199063
_cell_length_b                         3.199093
_cell_length_c                         40.000000
_cell_angle_alpha                      90.000000
_cell_angle_beta                       90.000000
_cell_angle_gamma                      120.000221
_cell_volume                           354.518853
_space_group_name_H-M_alt              'P 1'
_space_group_IT_number                 1

loop_
_space_group_symop_operation_xyz
   'x, y, z'

loop_
   _atom_site_label
   _atom_site_occupancy
   _atom_site_fract_x
   _atom_site_fract_y
   _atom_site_fract_z
   _atom_site_adp_type
   _atom_site_B_iso_or_equiv
   _atom_site_type_symbol
   W001        1.0    -0.333330     0.333335     0.292051    Biso  1.000000 W
   SE002       1.0     0.333338    -0.333335     0.250531    Biso  1.000000 Se
   SE003       1.0     0.333325    -0.333333     0.333571    Biso  1.000000 Se
```

119. WTe_2

```
_cell_length_a                         3.404187
_cell_length_b                         3.404191
_cell_length_c                         40.000000
_cell_angle_alpha                      90.000000
_cell_angle_beta                       90.000000
_cell_angle_gamma                      120.000031
_cell_volume                           401.437385
_space_group_name_H-M_alt              'P 1'
_space_group_IT_number                 1

loop_
_space_group_symop_operation_xyz
   'x, y, z'

loop_
   _atom_site_label
   _atom_site_occupancy
   _atom_site_fract_x
   _atom_site_fract_y
   _atom_site_fract_z
   _atom_site_adp_type
   _atom_site_B_iso_or_equiv
   _atom_site_type_symbol
   TE001       1.0     0.333334    -0.333333     0.251381    Biso  1.000000 Te
   TE002       1.0     0.333334    -0.333333     0.340165    Biso  1.000000 Te
   W003        1.0    -0.333335     0.333333     0.295773    Biso  1.000000 W
```
